# Epistatic evidence for gender-dependant slow neurotransmission signalling in substance use disorders: *PPP1R12B versus PPP1R1B*

**DOI:** 10.1016/j.ebiom.2020.103066

**Published:** 2020-10-21

**Authors:** Kefu Liu, Juan Zhao, Chunnuan Chen, Jie Xu, Richard L. Bell, Frank S. Hall, George F. Koob, Nora D. Volkow, Hong Qing, Zhicheng Lin

**Affiliations:** a School of Life Science, Beijing Institute of Technology, 100081 Beijing, China; b Laboratory of Psychiatric Neurogenomics, McLean Hospital, Belmont, MA 02478, United States of America; c Department of Neurology, the Second Affiliated Hospital of Fujian Medical University, Quanzhou, Fujian Province, P. R. China; d Department of Computer Information Systems, Bentley University, Waltham, MA, 02452, United States of America; e Department of Psychiatry, Institute of Psychiatric Research, Indiana University School of Medicine, Indianapolis, Indiana 46202, United States of America; f Department of Pharmacology and Experimental Therapeutics, College of Pharmacy and Pharmaceutical Sciences, University of Toledo, Toledo, Ohio 43614, United States of America; g National Institute on Drug Abuse and National Institute of Alcohol Abuse and Alcoholism, Bethesda, Maryland, 20892 United States of America

**Keywords:** Adolescence, Cell type-specific, Environmental risk, Missing heritability, Polysubstance abuse, Slow neurotransmission

## Abstract

**Background:**

Slow neurotransmission including DARPP-32 signalling is implicated in substance use disorders (SUDs) by experimental systems but not yet in the human aetiology. *PPP1R12B*, encoding another protein in the DARPP-32 family, hasn't been studied in the brain.

**Methods:**

Brain-regional gene activity was assessed in three different animal models of SUDs for mRNA level alterations. Genetic associations were assessed by meta-analysis of pre-existing dbGaP GWAS datasets for main effects and epistasis with known genetic risks, followed by cell type-specific pathway delineation. Parkinson's disease (PD) was included as a dopamine-related disease control for SUDs.

**Findings:**

In animal models of SUDs, environmentally-altered *PPP1R12B* expression sex-dependently involves motivation-related brain regions. In humans with polysubstance abuse, meta-analysis of pre-existing datasets revealed that *PPP1R12B* and *PPP1R1B*, although expressed in dopamine *vs.* dopamine-recipient neurons, exerted similar interactions with known genetic risks such as *ACTR1B* and *DRD2* in men but with *ADH1B, HGFAC* and *DRD3* in women. These interactions reached genome-wide significances (*P*_meta_<10^−20^) for SUDs but not for PD (disease selectivity: *P* = 4.8 × 10^−142^, OR = 6.7 for *PPP1R12B; P* = 8.0 × 10^−8^, OR = 2.1 for *PPP1R1B*). *CADM2* was the common risk in the molecular signalling regardless of gender and cell type.

**Interpretation:**

Gender-dependant slow neurotransmission may convey both genetic and environmental vulnerabilities selectively to SUDs.

**Funding:**

Grants from National Institute on Drug Abuse (NIDA) and National Institute on Alcohol Abuse and Alcoholism (NIAAA) of U.S.A. and National Natural Science Foundation of China (NSFC).

**Research in Context**

**Evidence before this study**

Aetiology of substance use disorders (SUDs) is incompletely understood. The dopamine- and cAMP-regulated neuronal phosphoprotein (DARPP-32, encoded by *PPP1R1B*) is a prototype mediator of slow neurotransmission and has been implicated in SUDs via animal models but evidence for humans is missing.

**Added value of this study**

The Protein Phosphatase 1 Regulatory Subunit 12B gene (*PPP1R12B*), another member of the DARPP-32 family, showed risk environment- and sex-dependant expression in three animal models of SUDs (alcohol and nicotine). Consistently in humans with polysubstance abuse, *PPP1R12B* and *PPP1R1B*, although expressed in different brain cell types, exerted similar interactions with known genetic risks in a gender-dependant and SUDs-selective manner.

**Implications of all the available evidence**

*PPP1R12B* and *PPP1R1B* cell type-specifically influence a selected vulnerability to develop SUDs both gender-dependently. Epistasis may uncover missing heritability alternatively sought for complex disorders such as SUDs. More interestingly, genetic and environmental risks may in fact utilize the same neural signalling as a systems disease mechanism in humans, supporting the development of precision medicine for SUDs.

Alt-text: Unlabelled box

## 1 Introduction

Substance abuse is the second leading cause of chronic diseases (behind hypertension) worldwide [1,2] but most of the substance use disorders (SUDs), including alcohol use disorder (AUD), still lack effective medications [3], warranting a better understanding of the disease mechanisms. It is well established that SUDs are attributable to both polygenic and environmental risks, including early life exposures to alcohol and smoke, but how the two category risks act together as a disease mechanism in humans remains elusive [4].

The dopamine- and cAMP-regulated neuronal phosphoprotein (DARPP-32, which is encoded by *PPP1R1B*) is a prototype mediator of slow neurotransmission implicated in SUDs by multiple experimental systems [5,6] (all capital letters reflect human nomenclature, whereas rodent nomenclature uses an italic font for gene and a plain font denotes the protein name). Previous studies already looked at Ppp1r1b (Darpp-32) expression in animal models and different brain regions [7], [8], [9]. During the last two decades, many studies have uncovered genetic risks for SUDs, such as *ADH1B/ADH1C, KLB, HGFAC, RABGAP1L, CADM2, ACTR1B, HIVEP2 and PPP1R1B* [10], [11], [12], [13] but never implicated any genes for the DARPP-32 signalling family with genome-wide significance. Little is known about how these signalling molecules contribute to environment- and/or sex/gender-related aetiologies of SUDs.

**Fig. 1:**
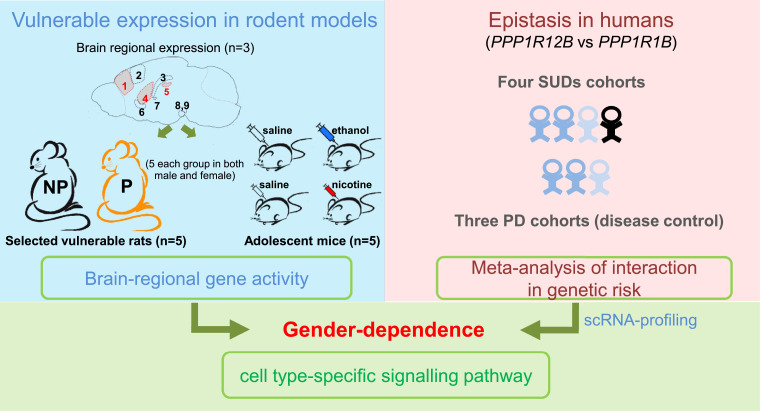
Study design. *Left*, brain regional expression (protein by IHF and mRNA by qPCR) in nine regions (1, mPFC; 2, M/PtA cortex; 3, Hippocampus; 4, CPU; 5, LHb; 6, NAc,; 7, CeA; 8, SNc and 9, VTA; red, three focused regions in the animal models) and altered gene activity (mRNA) in three regions (red) of three animal models, including P rats with SUDs, adolescent exposures to alcohol or nicotine (*n* = 5 males or females per group); *right*, genetics of signalling network with cell type-specific pathway in humans. scRNA, single cell RNA.

The Protein Phosphatase 1 Regulatory Subunit 12B gene (*PPP1R12B*, also called *MYPT2*), another member of DARPP-32 family, has never been the focus of any study on SUD, although a few genetic findings did mention *PPP1R12B* variants and its transcription activity in the development of SUDs [14,15]. Hence, we decided to test the hypothesis that this gene might provide insight into the slow transmission-related signalling mechanisms in SUDs, using three animal models plus a human genetic association approach. In the present study, as outlined in Fig. 1, we chose three common animal models to evaluate *PPP1R12B* activity in SUDs: drug-naïve rat alcohol model alcohol-preferring P/ alcohol-nonpreferring NP [16]; chronic exposures to alcohol and nicotine as environmental risks in adolescent mice [17], [18], [19], [20]_,_ in order not only to better understand the disease mechanisms but also to fully explore the singling mechanism. The animal work was paralleled by clinical validation of its genetic contribution to SUDs through secondary and meta-analyses of pre-existing, unrelated genome-wide association study (GWAS) datasets. Subsequently, distinct expression pattern between *Ppp1r12b* and *Ppp1r1b* based on published single-cell sequencing data help to clarify the genetic associations and imply pathway-based disease mechanisms.

The findings consistently suggest that both *PPP1R12B* and *PPP1R1B* cell type-specifically influence a selected vulnerability to develop SUDs in a gender-dependant manner and that epistatic mechanisms may uncover missing heritability alternatively sought for complex disorders [21]. More interestingly, this study also suggests that genetic and environmental risks may in fact utilize the same neural signalling as a disease mechanism in humans, facilitating the development of precision medicine for SUDs [22].

## 2 Methods

### 2.1 Ethics

All experimental procedures complied with animal use guidelines and ethical care as approved by the Institutional Animal Care and Use Committees (IACUC) of McLean Hospital for brain regional expression in Sprague Dawley (SD) rats (RGD_70,508), Beijing Institute of Technology (Animal Ethics Committee) (SYXK-BIT-2017-M15) for adolescent modelling in C57/BL6 (MGI:3,028,467) mice and brain regional expression in SD rats as well as IACUC of the Indiana University Schools of Dentistry and Medicine (Indianapolis, IN) for P (RGD_634,380) and NP (RGD_634,381) rats. Experimental animals were killed by cervical dislocation (for mouse) or cardiac perfusion (for rat) after finishing treatment for tissue collection.

### 2.2 Animals

All animals were housed under constant temperature- (21 °C) and humidity- (50%) on a 12 hrs/12 hrs light-dark cycle (light 7:00–19:00) with food and water available *ad libitum*. Animal models, brain regions examined, and sample size information are indicated in left panel of Fig. 1.

### 2.3 Evaluation of anti-PPP1R12B antibody specificity by Western blotting in brain tissue

Two-month old SD rat brains and sub-brain regions (caudate putamen (CPU), hippocampus and midbrain) were collected. After homologized in tissue lysis buffer, cells were disrupted by sonication, followed by centrifugation at 12,000 *g* in 4 °C to collect proteins in supernatant. Bradford Protein Assay (#5,000,201, Bio-Rad, Hercules, CA, USA) was used for protein quantification. Fifty μg protein was loaded onto a 10% Criterion TGX Precast Midi Protein Gel (#5,671,034, Bio-Rad). After resolved by electrophoresis, proteins were then transferred to PVDF membrane and stained with anti-PPP1R12B antibodies (H-71, 1:1000 dilution, sc-292,988, Santa Cruz Biotechnology Inc., CA, USA or AB_2,168,445, 1:5000 dilution, Proteintech Group., IL, USA), followed by incubation with HRP conjugated anti-rabbit antibody (AB_772,206, GE Healthcare, Chicago, IL, USA) and the staining was visualized with Pierce ECL substrate (#32,134, Thermo Fisher Scientific, Rockford, IL, USA). Images were captured by Chemi Doc XRS Molecular Imager (Bio-Rad), as described before [23].

### 2.4 Evaluation of Ppp1r12b distribution in brain by immunohistofluorescent (IHF) staining

Three 2-month old SD male rat brains were collected and fixed with 4% paraformaldehyde (PFA) for 24 h. After dehydration by 25% sucrose for cryoprotection, the brain was flash frozen at −80 °C and cut into 30 μm sections by freezing microtome and processed for IHF staining of Ppp1r12b, tyrosine hydroxylase (TH), and NeuN immunoreactivity according to previously described methods [23]. Brain sections were incubated first in blocking buffer (Life Technologies, CA, USA), and then with mixed antibodies, the rabbit polyclonal antibody (H-71 as anti-C for C-terminus, sc-292,988, Santa Cruz Biotechnology Inc. or AB_2,168,445 as anti-N for N-terminus, Proteintech Group, both at 1:500 dilution) and the mouse antibody (monoclonal anti-TH, AB_2,201,528, Millipore, Temecula, CA, USA or monoclonal anti-NeuN antibody, AB_2,298,772, Millipore, both diluted at 1:500), for incubation at 4 °C overnight. On the following day, sections were washed in Tris-buffered saline (TBS) and incubated with mixed fluorescent secondary antibodies (a 488 nm labelled goat anti-mouse (AB_141,838) and a 568 nm labelled goat anti-rabbit (AB_10,563,566) or a 555 nm labelled donkey anti-mouse (AB_2,536,180) and a 488 nm labelled donkey anti-rabbit (AB_2,535,792), both at 1:500 dilution and from Thermo Fisher Scientific) for 2 h. Sections/coverslips were washed and then covered with a drop of mounting buffer containing DAPI (AB_2,336,788, VECTOR LABORATORIES, Burlingame, CA, USA). Images were captured with a confocal microscope, Leica TCS SP8 (Leica Microsystems Inc. IL, USA). The staining intensities were quantitated by densitometry analysis using the NIH Image J program (NIH, Bethesda, MD, USA), followed by one-way ANOVA analysis using GraphPad Prism software. Each pair of regions was compared by Tukey *post hoc* tests.

### 2.5 Extraction of regions by brain dissection

To evaluate native *Ppp1r12b* expression pattern in adult brain, three 2 month-old C57BL/6 female mouse brains were each separated into nine regions, regions related to both dopamine neural circuitry and SUDs neurocircuitry [24,25], in an adult mouse brain slicer matrix [26]. These nine regions were medial prefrontal cortex (mPFC), CPU, nucleus accumbens (NAc), medial parietal association area (M/PtA) cortex, central nucleus of the amygdala (CeA), hippocampus, lateral habenular nucleus (LHb), substantia nigra (SNc), and ventral tegmental area (VTA), and collected for RNA isolation. In experiments with three animal models: early chronic alcohol exposure mice or chronic nicotine exposure mice with controls, and two month-old naïve P/NP rats, three regions of mPFC, CPU and LHb were selected for mRNA level assessment because these regions play key roles in SUDs [27], [28], [29], [30].

### 2.6 Measurement of mRNA levels in brain tissue by quantitative reverse transcription polymerase chain reaction (qRT-PCR)

qRT-PCR was conducted according to previously reported procedures [23]. The tissue RNA extraction protocol followed a procedure described in the TRIZOL reagent User Guide (#15,596, Invitrogen, CA, USA). For each sample, a mixture of 2 µg RNA with 1 µL of oligo dT15 was diluted with 17 µL diethyl pyrocarbonate (DEPC)-treated water and incubated at 70 °C for 10 min before adding the M-MLV Reverse Transcriptase (M1701, Promega, WI, USA) reaction mixture to synthesize cDNA. Each qRT-PCR reaction was prepared by SsoAdvanced universal SYBR® Green supermix (#172–5270, Bio-Rad) for rat tissue or by SYBR® Premix Ex Taq™ II (RR820A, Takara Bio USA Inc., Mountain View, CA, USA) for mouse tissue, with 200 nM of primer mixture and 1 µL of cDNA. Primers were ordered from Integrated DNA Technologies (USA) for rat genes and from Shanghai Sangon Biotech (China) for mouse genes. For each gene, two pairs of intron-spanning primers were designed and one of them was selected based on the observation of a single melting curve peak and an amplification coefficient of 2.0; the selected primers are listed in Table 1. The PCR program ran for 45 cycles, with an annealing temperature of 56 °C, on a Bio-Rad CFX C1000 Real-Time PCR Detection System (Bio-Rad) according to the manufacturer's protocol. The efficiency (an average coefficient of 2.0) was calculated using a series dilution method and Bio-Rad CFX Manager software. Each coefficient was used in fold-change calculations for each primer pair. The mRNA expression level used GAPDH as an input control.

**Table 1:** qRT-PCR primers information* in this study (r, rat; m, mouse).

Primer Name	Primer Sequence
rPpp1r12b-F	5′-CTTCCTGTCCACCTCACTT-3′
rPpp1r12b-R	5′-CCAGACCTGACCTCGTCTA-3′
rGapdh-F	5′-ATGACTCTACCCACGGCAAG-3′
rGapdh-R	5′-TACTCAGCACCAGCATCACC-3′
mPpp1r12b-F	5′-CCTTAGGGATCGAGGTTCTT-3′
mPpp1r12b-R	5′-AACAGCTGACTCTCTGTTCT-3′
mGapdh-F	5′-CTCGTCCCGTAGACAAAATG-3′
mGapdh-R	5′-GATGGCAACAATCTCCACTT-3′

⁎ see Supplementary Fig. 1 for sequence specificity.

### 2.7 Treatment in SUDs animal models

For evaluation of Ppp1r12b change in animal models, estimation of sample size, based on a reported method [31] and on our previous experiences with minimum significant fold change in gene expression (1.32-fold and its standard deviation of 0.17) [13,23], resulted in 4.92 (*n* = 5) per group for a level of significance at 5%, power at 80% and attrition rate at 10%. We thus chose five animals for each treatment group in all modelling experiments. A total of 40 mice and 20 rats in experiments of SUD animal models (5 control and 5 treatment female mice or rats in three female SUD models; 5 control and 5 treatment male mice or rats in three male SUD models) were used. Five mice or rats in each group were housed individually in home cages. We used a pseudo randomization method to allocate the mice. No animals were excluded in the experiments. Each animal was marked with a random number and processed for experimental analysis. Data were collected individually and then grouped based on marker in data analysis.

The adolescent period for a mouse is considered as four to eight week old [20]. To evaluate *Ppp1r12b* mRNA levels after chronic (4-week) exposure to alcohol or nicotine during adolescence, four-week old C57BL/6 mice were used.

For the chronic alcohol exposure experiment, mice (5 males each group or 5 females each group) were injected intraperitoneally (i.p.) with 25% ethanol (u1012772, Sinopharm Chemical Reagent Beijing, China) in saline (v/v) or equal-volume saline (control group) daily for six consecutive days at a dose of 15 µL/g body weight. This dose regimen has been previously used to identify changes in cellular function and gene transcription [32,33]. For the chronic nicotine exposure experiment, five male mice were injected daily with 2.5 mg/kg/day nicotine (#N3876, Sigma, St. Louis, MO, USA) i.p. or equal-volume saline (5 mice too in control group), for 28 consecutive days [34,35]. Female subjects were also treated the same way as the males except the dosage was 5 mg/kg/day (male mice were more sensitive to nicotine, the dosage of 5 mg/kg/day nicotine was lethal for males but not for female mice in our preliminary experiment, data not shown).

### 2.8 Secondary analysis of dbGaP GWAS genotype

*Genetic analyses* used three cleaned GWAS datasets containing four cohorts for SUDs, and another three independent datasets for Parkinson's disease (PD) (as a dopamine-related disease control). The SUD datasets covered polysubstance abuse, including alcohol, cigarette and cocaine use disorders, which are phs000125.v1.p1 by Collaborative Study on the Genetics of Alcoholism (COGA), phs000092.v1.p1 by Study of Addiction: Genetics and Environment (SAGE), and phs000181.v1.p1 by the Australian twin-family study of alcohol use disorder (OZALC). The COGA dataset was split into two ethnic datasets, European Americans and African Americans, so that three datasets became four SUDs cohorts. Basic information, including mean age of about 40 years, of the datasets has been published before [36,37]. For PD, the three independent case-control studies from dbGaP were phg000126.v1.p1 by the Centre for Inherited Disease Research (CIDR), phs000089.v3.p2 by the National Institute of Neurological Disorders and Stroke (NINDS) and phs000196.v2.p1 by the NeuroGenetics Research Consortium (NGRC). A data clean method was as reported before [13,23,38]. Briefly, standard quality control procedures were used to extract the unrelated individuals [39]. Quality control filters for SNPs included a minor allele frequency >5% and a missing genotype rate of <5%. After genomic quality control, more than 6500 unrelated subjects were used: 6596 unrelated with 53.6% females for SUD datasets; 6572 unrelated with 48.6% females for PD datasets. Imputation was carried for each of these four cleaned datasets as described before [40], in order to extend genotype coverage. Data manipulation, allelic association, and meta-analysis were carried out using PLINK [41]; case-control logistic regression analysis of inter-SNP interactions was performed in the CASSI 2.50 software and *P* values were used to evaluate the presence or strength of interactions [42].

*Interaction results* were displayed via R programming (R 3.5.1, www.r-project.org), which was implemented using a reported circlize package (https://cran.r-project.org/web/packages/circlize/index.html) [43]. *P* values and odds ratios (ORs) of selectivity at gender, disease or gene levels were calculated by Chi-square tests.

### 2.9 Single-cell RNA sequencing data extraction and analysis

To evaluate cellular Ppp1r12b *vs*. Ppp1r1b expression in the brain, single-cell RNA (scRNA) sequencing data from mouse TH+ neurons (GSE108020) [44], mouse Drd1+ or Drd2+ cells (GSE112177) in dorsal striatum [45] and human cortex neurons (GSE67835) [46] were downloaded from GEO datasets website. Mouse Drd2+ cells translational profiling (GSE141463) used a BAC transgenic Translating Ribosome Affinity Purification (BacTRAP) strategy, allowing cell type-specific profiling of complex tissue [47]. Gene activity was normalized with GAPDH in each cell (cells without GAPDH counts were excluded).

Pathway analysis was carried out using MetaCore database, as previously described [48], [49], [50] to identify biologically relevant pathways.

### 2.10 Statistics analysis

All data are presented as mean ± *s*.e.m. (standard error of the mean). Each animal was considered as one experimental unit. One- or three-way ANOVAs followed by Tukey *post hoc* comparisons were used in brain regional expression analysis and for assessing interactions with sex. Two-tailed t-tests (setting α=0.05 as statistically significant) were used for pair-wise expression analysis. These analyses were conducted using GraphPad Prism _7_ software as mentioned above. *P*_meta_ values from GWAS data meta-analysis were subjected to multiple testing by Bonferroni method and original *P*_meta_ values only surviving the testing are shown.

## 3 Results

### 3.1 Heterogeneous expression of Ppp1r12b in the brain

**Fig. 2:**
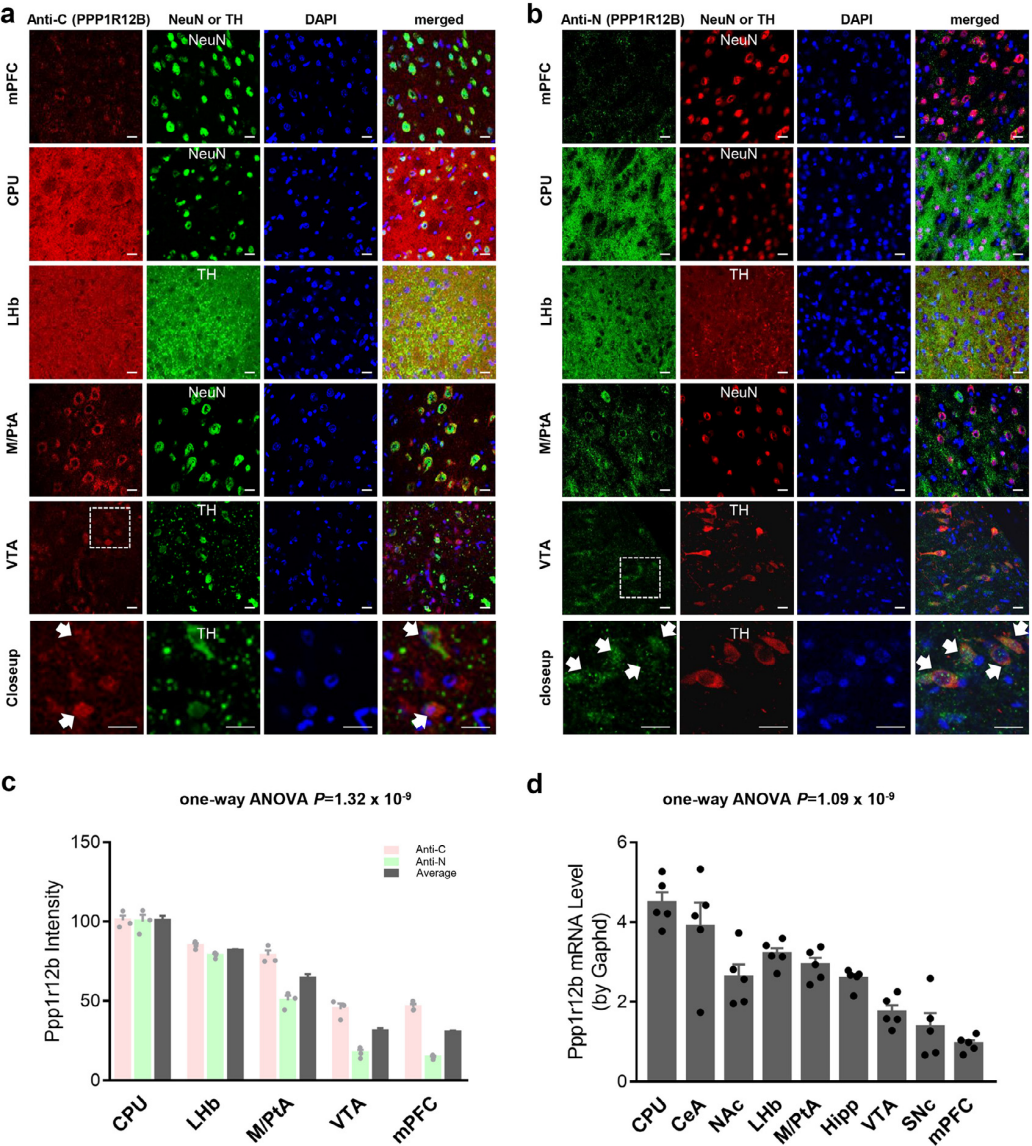
Ppp1r12b expression in different brain regions. (**a-b**) Immunoreactivities in five representative rat brain regions (mPFC, CPU, LHb, M/PtA and VTA) are shown here. Left column, PPP1R12B antibody (Anti-C, in red) recognized C-terminus in **a**; PPP1R12B antibody (Anti-N, in green) recognized N-terminus in **b**; second and third columns, anti-NeuN or anti-TH (green or red), and DAPI (blue); right column shows the merged staining. Clear neuronal staining was observed in VTA, mPFC and M/PtA; diffuse staining was observed in the LHb and CPU, consistently. In the bottom rows as a closeup of VTA, arrows indicate co-localization of Ppp1r12b and TH in dopamine neurons. Scale bars, 50 µm (other regions examined but not shown here: SNc was similar to VTA with a distinct cellular pattern; CeA and NAc were similar to diffuse CPU; hippocampus had a pattern in between mPFC and LHb). (**c**) Densitometry analysis of Ppp1r12b immunoreactivities observed in **a** (in light red) and **b** (in light green), and the average (in dark grey) of immunoreactivities, showing regional expression which was verified by one-way ANOVA results (*n* = 3). Information for additional regions was collected but less consistent so not shown. (**d**) Mouse brain regional expression by mRNA levels. Differential expression was verified by one-way ANOVA analysis. VTA and mPFC had no significant difference by Tukey's multiple comparisons (*P* = 0.29) (*n* = 5/group).

Regional expression of Ppp1r12b immunoreactivity in the brain has not been previously characterized and there is no complete gene knockout model available. Western blot data thus failed to show any antibody specificity clearly although one (Anti-N) of the antibodies seemed able to detect a denatured brain protein on the gels (data not shown). To cross-verify specific Ppp1r12b expression, two different PPP1r12B antibodies were used, one (Anti-C, Fig. 2a) was raised by using the C-terminal amino acids 861–931 as the peptide antigen; another (Anti-N, Fig. 2b) was raised by using the N-terminal amino acids 1–386 as the peptide antigen. Fig. 2a-b show the regional expression patterns on Ppp1r12b in mPFC, CPU, LHb, VTA and M/PtA cortex alongside staining for NeuN (mark for neurons), TH (mark for dopamine neurons) or DAPI (mark for nucleus) for the merged images obtained from 2 month-old male SD rats. Distinct cellular expression of Ppp1r12b was found in M/PtA, VTA, and mPFC neurons, while diffuse expression was found in CPU and LHb (SNc was similar to VTA with a distinct cellular pattern; CeA and NAc were similar to diffuse CPU; hippocampus had a pattern in between mPFC and LHb, data not shown). The cellular staining in M/PtA, VTA and mPFC was localized to the cell bodies and substantially overlapped with the neuronal marker NeuN and partially with the nuclear marker DAPI. The CPU and LHb have large amounts of dopaminergic terminal regions, the diffuse staining for Ppp1r12b overlapped substantially with TH staining in the LHb. These data showed substantial expression of Ppp1r12b in dopaminergic cell bodies (see the bottom or “closeup” rows in Fig. 2a, b) and terminal regions, indicating that it is likely to play a role in dopaminergic function such as incentive salience. Consistent staining results between the two PPP1R12B antibodies suggested that the observed immunoreactivities represented the real Ppp1r12b expression pattern in the brain. Densitometry analysis of Ppp1r12b immunoreactivity (Fig. 2c) revealed that Ppp1r12b protein expression differed significantly amongst brain sub-regions (*P*<0.0001 by one-way ANOVA). The highest Ppp1r12b protein expression levels amongst these five sub-regions were in the CPU and the lowest in the mPFC and VTA. Ppp1r12b and Ppp1r12a are both expressed in the brain, sequence similarity (48.2% identity and 73.4% homology in rat) between these two proteins could cause cross-reactivity in antibody-based experiments [51]. PPP1R12B antibody H-71 was raised against amino acids 861–931 of PPP1R12B, a peptide sequence with 94% identity to rat Ppp1r12b but only 43% identity to rat Ppp1r12a (Supplementary Fig. 1a) suggesting that cross-reactivity to Ppp1r12a was unlikely.

To further evaluate this regional expression pattern, *Ppp1r12b* expression was examined independently at the mRNA level in mouse brain, using *Ppp1r12b*-specific PCR primers (Supplementary Fig. 1c). Nine sub-regions were examined which differed significantly in mRNA levels (*P*<0.0001, one-way ANOVA) (Fig. 2d). The highest tissue density of *Ppp1r12b* mRNA was found in the CPU, with the CeA and LHb having the next highest levels, which were consistent with some protein measurements using IHF staining. The lowest density was found in the mPFC, also consistent with the IHF observations.

Overall, these mRNA data paralleled the IHF data on protein density. Based on this pattern of expression, the following modelling experiments focused on three selected brain regions, CPU, LHb and mPFC, as these regions play key roles in SUDs [27], [28], [29], [30], for gene transcriptional activity in three rodent models relevant to SUDs: (a) P *vs.* NP naïve rat model, (b) adolescent chronic ethanol exposure in mice, and (c) adolescent chronic nicotine exposure, in both male and female mice.

### 3.2 Brain region- and sex-dependant alterations in Ppp1r12b mRNA levels in the P/NP rat model

**Fig. 3:**
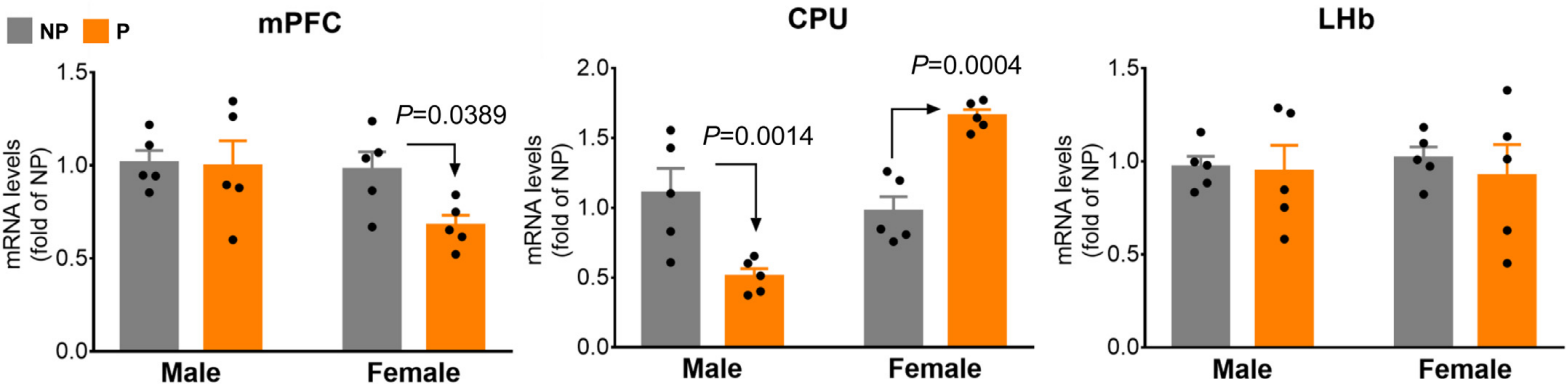
Sex- and brain region-dependant levels of *Ppp1r12b* mRNA in NP *vs.* P rats: *left*, mPFC; *middle*, CPU; *right*, LHb. The *t*-test-based exact *P* values of <0.05 only are showed in graphs; 3-way ANOVA implied significant model interaction with sex (*P* = 0.0161) (*n* = 5/group).

The P *vs.* NP rat model is a bidirectionally selectively bred model for high *vs.* low ethanol-drinking phenotypes, which has been widely used in preclinical studies of AUD [16]. We compared *Ppp1r12b* mRNA levels between P and NP rats separately for males and females (Fig. 3). In the mPFC, female P rats had lower levels (by 29.9%, *P* = 0.0389, unpaired two-tailed t-tests) than NP females but there were no differences in males (*P* = 0.895, unpaired two-tailed t-tests); in the CPU, levels were lower in male P rats (by 50.9%, *P* = 0.0014, unpaired two-tailed t-tests), and higher in female P rats (by 68.3%, *P* = 0.0004, unpaired two-tailed t-tests) compared with their NP counterparts; in the LHb, there were no significant differences between P and NP rats of either sex (*P* = 0.88 for males and *P* = 0.57 for females: unpaired two-tailed t-tests). These findings showed that in the rodent brain *Ppp1r12b* gene expression was sex and region dependant and that it differed in a rat model selected for their alcohol preferences over water (P *vs* NP).

### 3.3 Brain region- and substance-dependant regulation of Ppp1r12b mRNA levels by chronic ethanol or chronic nicotine administration in adolescent male mice

**Fig. 4:**
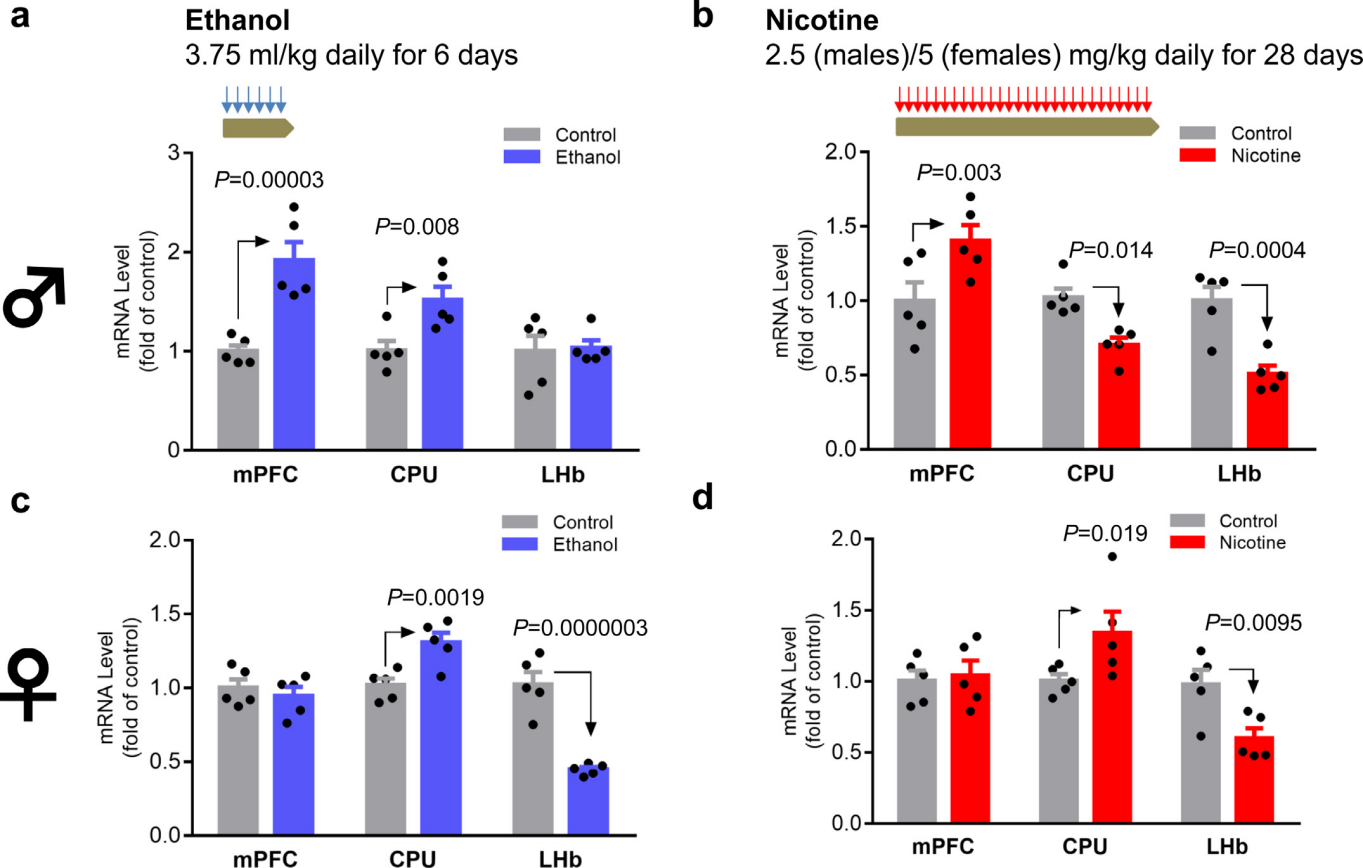
Tissue-dependant regulation of *Ppp1r12b* mRNA by chronic ethanol (a,c; blue) or nicotine (b,d; red) exposure in male (a,b) or female (c,d) mice. Procedures for chronic treatments are indicated on top of whole figure, the symbol♂ for male and ♀ for female are showed on left of whole figure. Note that males and females had different nicotine doses; the *t*-tests-based exact *P* values of <0.05 are showed in graphs; 3-way ANOVA implied significant ethanol interaction with sex (*P*<0.0001) (*n* = 5/group).

Chronic exposure to ethanol in adolescent male mice increased *Ppp1r12b* expression by 91.7% in the mPFC (*P*<0.0001, unpaired two-tailed t-tests) and by 51.9% in the CPU (*P* = 0.008, unpaired two-tailed t-tests) but had no effect on expression in the LHb (*P* = 0.841, unpaired two-tailed t-tests) (Fig. 4a). To explore whether *Ppp1r12b* was affected by other drugs-of-abuse, we also assessed the effects of chronic nicotine exposure on *Ppp1r12b* expression. Chronic nicotine increased *Ppp1r12b* expression in the mPFC, by 40.6% (*P* = 0.003, unpaired two-tailed t-tests), but it decreased expression in the CPU by 32.0% (*P* = 0.014, unpaired two-tailed t-tests) and in the LHb by 49.1% (*P* = 0.0004, unpaired two-tailed t-tests) (Fig. 4b). These findings showed that, *Ppp1r12b* gene expression was regulated by both ethanol and nicotine, in a partially substance- and region-dependant manner; whereas both drugs-of-abuse increased expression in the mPFC, they had opposite effects in the CPU and only nicotine affected expression in the LHb.

### 3.4 Brain region- and substance-dependant regulation of Ppp1r12b mRNA levels by chronic ethanol or nicotine in adolescent female mice

**Fig. 5:**
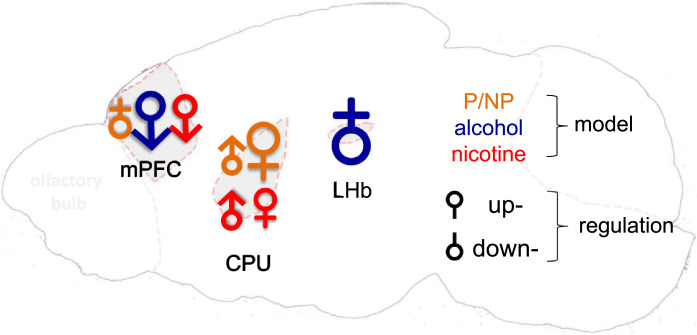
Summary of sex-dependences in model- and region-related *Ppp1r12b* mRNA levels. Symbol:♂ for male and ♀ for female; orientation of symbols, for up or down-regulation; size of symbol, extent of regulation (not to scale), in three regions (mPFC, CPU and LHb); different colours, different rodent models as indicated.

In adolescent female mice, chronic exposures to ethanol or nicotine increased *Ppp1r12b* expression by approximately 30% in the CPU (*P* = 0.0019 for ethanol and *P* = 0.019 for nicotine: unpaired two-tailed t-tests) (Fig. 4c and d). In contrast, chronic exposure to ethanol or nicotine decreased *Ppp1r12b* gene expression in the LHb, by 57.6% (*P*<0.0001, unpaired two-tailed t-tests) and 37.9% (*P* = 0.0095, unpaired two-tailed t-tests), respectively, but neither drug affected expression in the mPFC (*P* = 0.53 for ethanol and *P* = 0.77 for nicotine: unpaired two-tailed t-tests). These sex-dependence data from three rodent models are merged in Fig. 5, showing also model- and brain region-dependence of this gene's activity.

#### 3.4.1 Gender-dependant interactions with known genetic risks for developing SUDs

##### 3.4.1.1 PPP1R12B

Next, we investigated in humans whether *PPP1R12B* confers any genetic risk for SUDs. After meta-analysis of four cohorts (SAGE, OZALC and two cohorts in the COGA dataset: European Americans and African Americans), few main effects were found: *P*_meta_=0.032 for rs11587179 (OR_meta_=0.82) and *P*_meta_=0.038 for rs10494832 (OR_meta_=0.83) in males only (they were 1423 bp apart located in a middle intron and both results were supported by all three cohorts); no significant signals (*P*_meta_>0.06) were found in females or when the genders were combined (data not shown).

**Fig. 6:**
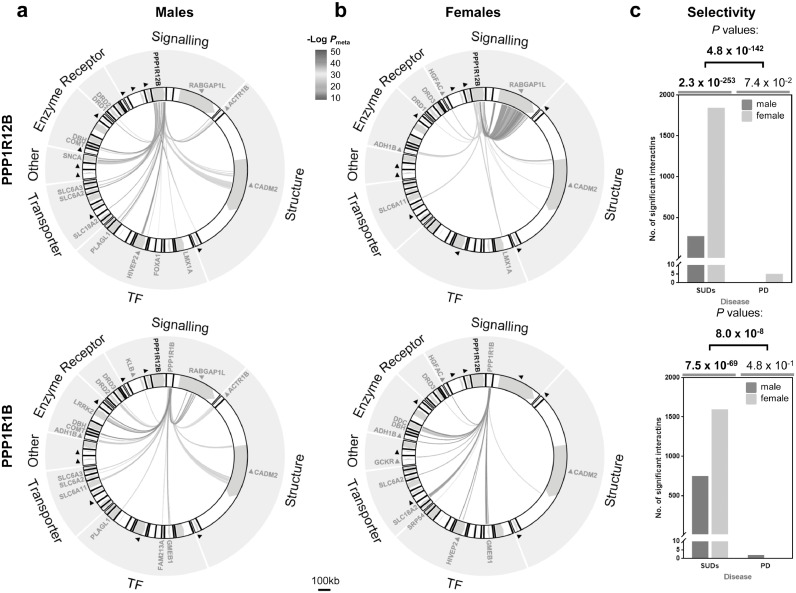
Gender-dependant *PPP1R12B* (top) or *PPP1R1B* (bottom) interactions with other genes in SUDs: (**a**) males; (**b**) females. 46 genes, organized in a wheel here in seven categories (enzyme, receptors, signalling, structure, TF, transporter and other; also listed in Supplementary Table 1), were included in this epistasis analysis. TF, transcription factors (*e.g., HIVEP2* and unpublished *PLAGL1*); interacting genes are labelled in red; black triangle, known risk for SUDs, per reported meta-analyses (no interaction found for PD in this 46 genes-network (see Supplementary Fig. 2; also for the full labelling of genes). All interactions shown here reached statistical significance after Bonferroni correction and most of them reached absolute genome-wide significance (*P*_meta_<10^−20^), referring to the thermometer bar; *FOXA1* SNPs might represent next gene *TTC6* and *ADH1B* SNPs might represent next gene *ADH1C* too; scale bar, 100 kb. (**c**) Selective contribution of *PPP1R12B* (top) or *PPP1R1B* (bottom) to SUDs (top *P* value, from gender combined data), especially in females (*P* value grey-underlined, OR=6.7 (*PPP1R12B*) and 2.1 (*PPP1R1B*)) comparing to PD, based on number of statistically significant interactions; gender-specific selectivity for SUDs over PD: *P* = 1.6 × 10^−28^ in males and 4.1 × 10^−178^ (OR=164) in females in *PPP1R12B* and *P* = 5.9 × 10^−23^ (OR=50.8) in males and 4.7 × 10^−49^ in females in *PPP1R1B*.

However, meta-analysis of logistic regressions for case-control association in the four SUD cohorts revealed extensive and significant interactions of *PPP1R12B* with some known risk genes for SUDs. For this interaction analysis, we composed a 46 gene-network for potential PPP1R12B signalling, including plausible dopaminergic genes for receptors, transporters, enzymes and transcription factors (TFs), as well as reported genetic risks [10], [11], [12], [13] for SUDs (see Supplementary Table 1 for details). Totally, 1,353,065 male unique variants, 1,342,114 female variants, and 1,391,155 mixed variants were analysed; <10% of them were eligible for meta-analysis. The interacting variants between two genes were independent of each other, according to their distance farther than 500 kb [41]. More interestingly, such interactions were gender-dependant. In males, *PPP1R12B* interacted 274-times (statistically significant) with 15 genes, including four reported risks *CADM2, ACTR1B, RABGAP1L* and *HIVEP2*, along with three TFs *LMX1A, FOXA1/TTC6 (*unknown function*)* and *PLAGL1*, three transporters *SLC6A3, SLC6A2* and *SLC18A2*, two dopamine receptors *DRD1* and *DRD2*, two dopamine catabolism enzymes *DBH* and *COMT*, and also *SNCA* (Fig. 6a *upper panel*). In females, the interactions showed a different pattern: it interacted 1844-times (statistically significant) with eight genes, including four reported risks *RABGAP1L, CADM2, HGFAC and ADH1C/ADH1B* which were next to each other, along with also the TF *LMX1A*, one transporter gene *SLC6A11* (which encodes GAT-3 [52]), and two dopamine receptor genes *DRD1* and *DRD3* (Fig. 6b *upper panel*). In both genders, *PPP1R12B* interacted with *CADM2, LMX1A* and *DRD1* but in two different sets of single nucleotide polymorphisms (SNPs), as indicated by the different patterns between two genders and also detailed in Supplementary Table 2. Note that the following interactions reached absolute genome-wide (GW) significance (*P*_meta_<10^−20^): ten (*LMX1A, RABGAP1L, ACTR1B, CADM2, SNCA, PLAGL1, DBH, DRD2, SLC6A2 and COMT*) in males, eight or nine (*LMX1A, RABGAP1L, SLC6A11, CADM2, DRD1, DRD3, HGFAC, and ADH1C/ADH1B*) in females (underline, three shared with males), and nine genes (*RABGAP1L, CADM2, DRD3, SNCA, SLC18A1, HIVEP2, DBH, LRRK2, COMT and KLB/RPL9/LIAS*) when both genders were combined (*LIAS* was for Lipoic Acid Synthetase; *RPL9*, for Ribosomal Protein L9). All significant details are provided in Supplementary Table 2. A notable gender difference (chi-square=1156; *P* = 2.3 × 10^−253^) was the larger interactions of *PPP1R12B* with risk genes in females (1844 times) *versus* males (274 times) as well as the high density of interactions with RABGAP1L in females.

As a control, dopamine-related Parkinson's disease (PD) was included in these case-control association analyses and 1.9~2.2 million unique variants were analysed. *PPP1R12B* interacted twice with *SLC6A11* (GW significant, *P*_meta_=2.62 × 10^−21^ for rs4520471-rs1807318 and *P*_meta_=7.05 × 10^−21^ for rs1968583-rs1807318), once with *LMX1A* (*P*_meta_=2.72 × 10^−22^ for rs4619029-rs142166300), *KLB* (*P*_meta_=1.12 × 10^−19^ for rs6427957-rs111408859) or *DBH* (*P*_meta_=6.15 × 10^−15^ for rs73087530-rs3025383) in females only; no significant interactions were found for males or when the genders combined (Supplementary Fig. 2). There was no gender difference (chi-square=3.2; *P* = 0.07) in PD. Therefore, *PPP1R12B* displays a selective and significant contribution to a risk for developing an SUD (chi-square=810; *P* = 4.1 × 10^−178^, Fig. 6c *upper panel*).

##### 3.4.1.2 PPP1R1B

Meta-analysis of main effects did not find any positive signals for this gene, regardless of gender and disease. However, the meta-analysis of case-control epistasis also revealed gender-dependant interactions with some known risk genes for SUDs. In males, *PPP1R1B* interacted with 16 genes, including *CADM2, ACTR1B, RABGAP1L* and *DRD2* (Fig. 6a *lower panel*). In females, the interactions showed also a different pattern: it interacted with 12 genes, including four reported risks *CADM2, HGFAC, HIVEP2 and ADH1C/ADH1B* (Fig. 6b *lower panel*). As for PD, *PPP1R1B* displayed little interaction in this network, and thus a selective contribution to a risk for developing a SUD as well (Fig. 6c *lower panel*). *CADM2* was present in all human interacting networks revealed in this study.

### 3.5 Cell type-specific

**Fig. 7:**
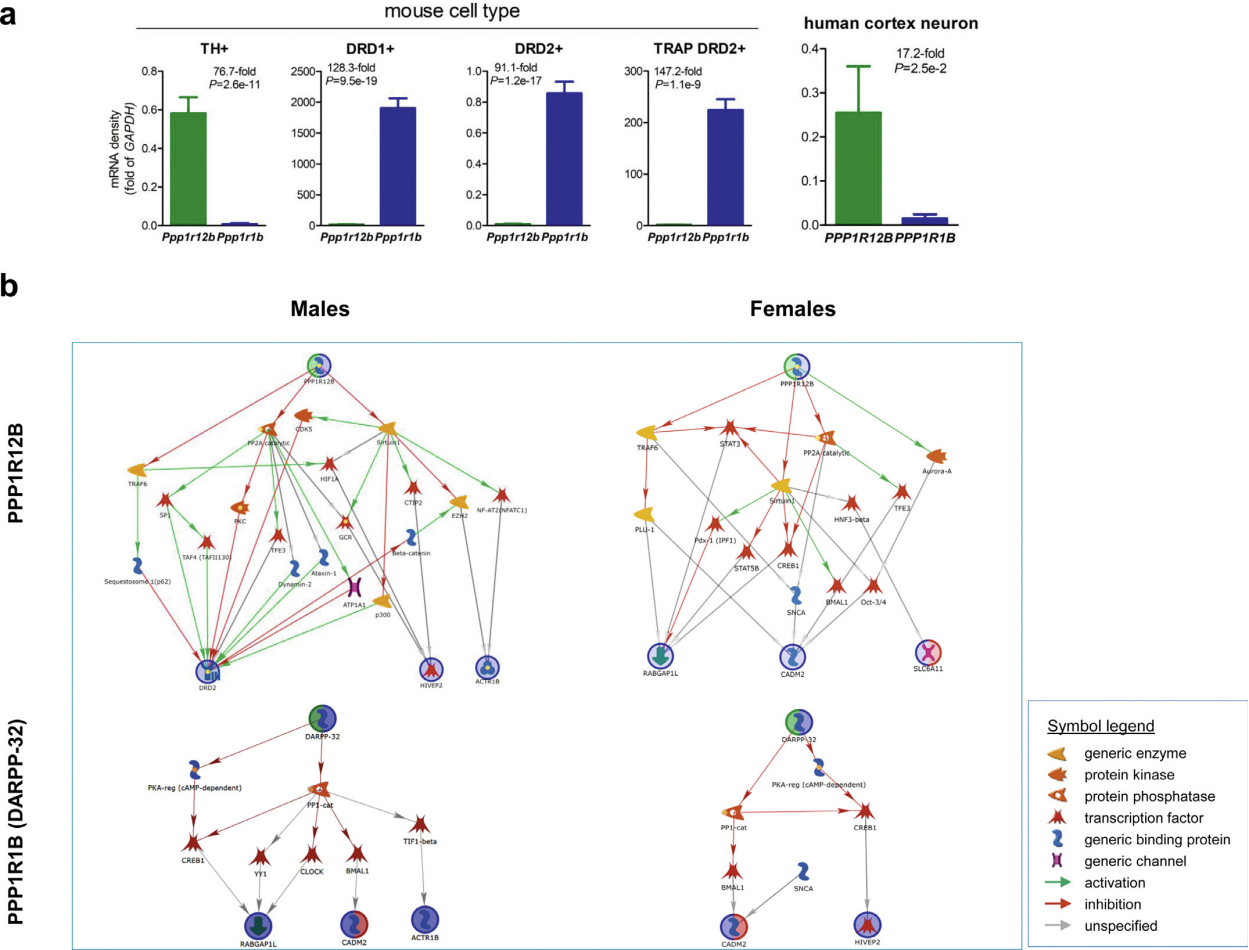
Cell type-specific pathways for PPP1R12B or PPP1R1B (DARPP-32) to interact with other genetic risks in a gender-dependant manner, based on epistasis in Fig. 6. (**a**) Cell type specific expression of Ppp1r12b and Ppp1r1b (cell number used: *n* = 417 for TH+, *n* = 40 for DRD1+, *n* = 40 for DRD2+, *n* = 10 for TRAP DRD2+ of mouse origin, and *n* = 129 for human cortex neurons), based on single cell RNA (scRNA) profiling except BacTRAP strategy sequencing for TRAP DRD2+. GAPDH was used to normalize for relative density of mRNA here in each cell and had very low density comparing to beta-actin in DRD1+ and TRAP DRD2+ cells. *P* values were from two-tailed t-tests. (**b**) Pathways: *upper panels*, for PPP1R12B in TH+ cells and lower panels, for PPP1R1B (DARPP-32) in dorsal striatal DRD1+ cells; left for males and right for females; all activity in pathway is expressed in the indicated cell type, based on the scRNA profiling.

Finally, public single-cell sequencing data were used to clarify whether *PPP1R12B, PPP1R1B* and interacting risk factors co-express in the same cells. As the result, PPP1R12B was expressed mainly in dopamine neurons and *PPP1R1B*, mainly in dopamine-receptive neurons (Fig. 7a). Accordingly, different pathways are revealed related to *PPP1R12B vs. PPP1R1B* and gender (Fig. 7b).

## 4 Discussion

Although the incentive of this work was weak, surprisingly the findings identify significant environmentally sensitive genetic risks in slow neurotransmission which may involve the aetiology of SUDs. This information may have an implication for developing personalized treatment, besides medication, for these complex disorders. At a molecular signalling level, the phosphoproteins of the DARPP-32 family function as signalling molecules for slow neurotransmission interacting with other proteins to impact physiological outcomes. Such multicomponent interacting pathway mode of operation could explain the lack of genetic evidence on their own *via* main effects or allelic associations while studies from other fields have presented pathway-specific genetic risks for other diseases [53], [54], [55].

Epistasis represents an alternative aspect of genetic aetiology for complex diseases [56], [57], [58], [59] but interpretation of the results with different types of interactions [60], [61], [62], [63], [64] may be more challenging than for main effects. In biology however, proteins or genes function dependently on many other activities in most cases. If a protein activates another, gain-of-function allele of the former may activates loss-of-function variant of the later, resulting in no change in overall activation. This example explains the biological significance of considering epistasis. Our case-control epistasis analysis exploits the consideration of signalling pathways, which require exactly inter-activity dependence, in clarifying their selective genetic contribution to SUDs. Consistently both *PPP1R12B* and *PPP1R1B* showed significant epistasis evidence. Moreover, *PPP1R1B* also showed selective contribution in females but with less gender dependence than *PPP1R12B* (OR 2.1 *vs.* 6.7; chi-square 308 *vs.* 1156; *P* = 7.5 × 10^−69^ *vs.* 2.3 × 10^−253^). For PD, none of *PPP1R1B*’s interactions reach genome-wide significances, with or without gender stratification.

Results from animal models help clarify the aetiology in humans. The present experiments examined *Ppp1r12b* gene expression, showing that it is expressed in several brain regions relevant to the effects of ethanol and nicotine [65,66], and that the level of expression was altered in models relevant to SUDs. In fact, the P rat is vulnerable for excessive self-administration of a number of drugs-of-abuse including alcohol, nicotine and cocaine [16,[67], [68], [69], [70]], so that the sex-dependant gene activity observed in all of the rodent models mirrors the gender-dependant association findings from the cohorts with alcohol, cigarette and cocaine use disorders (Fig. 6a-c). Importantly, different models show the same direction of regulation by known risks for the same sex. For example, in CPU, *Ppp1r12b* is down-regulated in males but up-regulated in females by both P/NP and nicotine exposure; in mPFC, it is up-regulated in males by both alcohol and nicotine exposures (Fig. 5, a summary of rodent data). These results consistently support the gender-dependant *PPP1R12B* association findings in humans, verify a common role of slow neurotransmission in the pathophysiology, and perhaps indeed help uncover missing heritability of SUDs.

Delineation of pathways may enable understanding disease mechanisms [71,72] so that uncovering related pathways seems critical in terms of slow transmission. Based on the brain regional expression, we first examined in databases whether these genes are expressed in different types of cells in order to 1) clarify the epistasis information and 2) identify biologically relevant pathways. As Fig. 7a shows, *PPP1R12B* and *PPP1R1B* are not co-expressed in the same cells, which may explain the lack of any interaction between these two genes. The former is expressed in dopamine neurons and the later, in DRD1- and DRD2-expressing cells in the dorsal striatum. Accordingly, four pathways are revealed, reflecting the epistasis, gender dependence and cell type (Fig. 7b). Three main features are noticed here. First, dopaminergic pathways are more involved than the non-dopaminergic pathways, which is consistent with an established view that altered dopamine signalling contributes to SUDs. Second, almost a half of the interacting members are TFs, supporting the genetic role of molecular signalling in the aetiology. Third, many of the interactions are not specified yet (grey arrows), providing opportunities for hypothesis testing. For example, how the small protein SNCA regulates the matrix protein CADM2 and how the known genetic risks are transcriptionally regulated. Testing of pathway-generated hypotheses will help understand epistasis as well, empowering an “envirgenetic” prediction of complex phenotypes.

Limitations of this study include lack of rodent data on activity in other risk genes such as *Rabgap1L, Cadm2* and *Actr1b*; as well as lack of functional genetic evidence for the epistasis. Future study is warranted to delineate the biological activity of the epistasis in experimental systems.

**Fig. 8:**
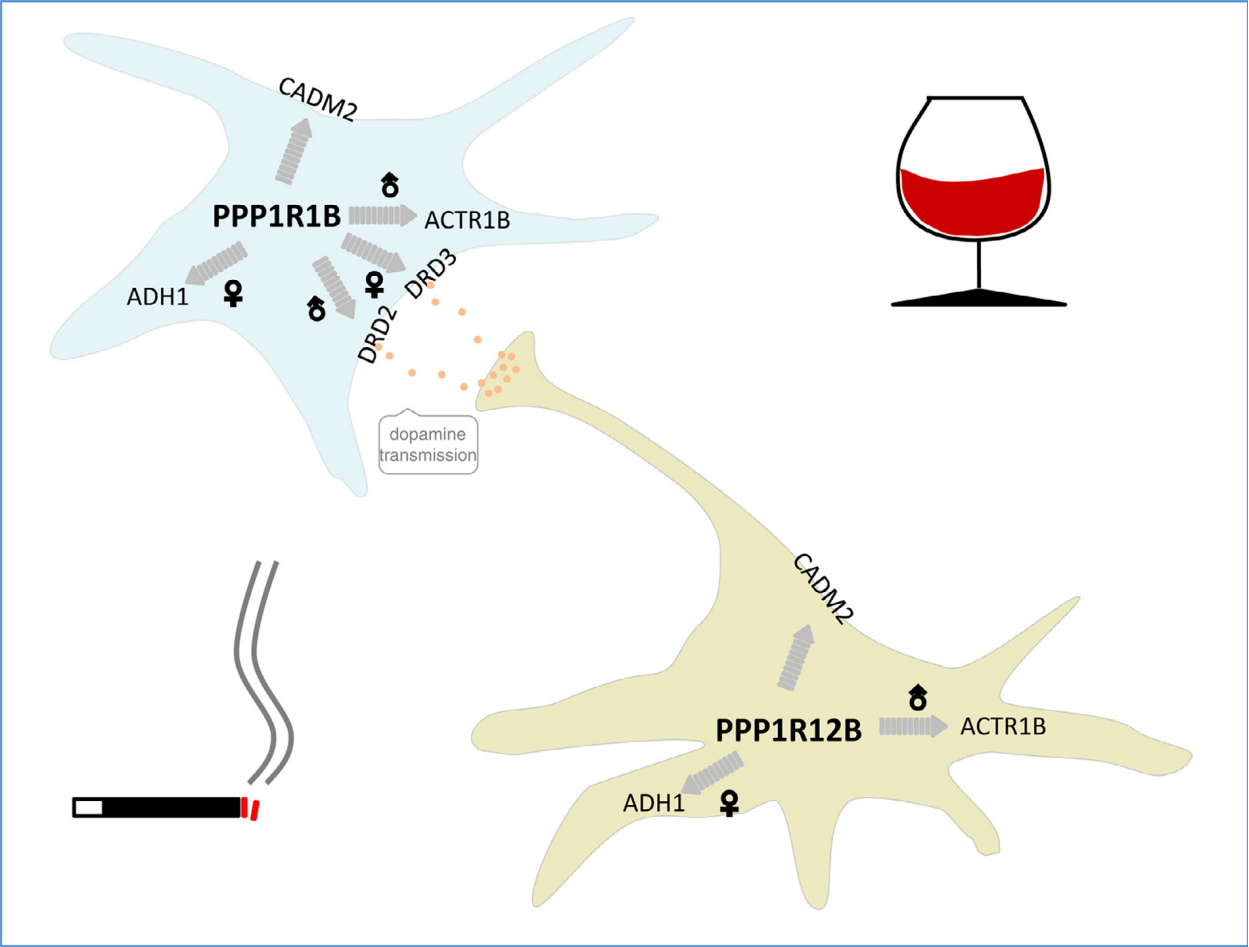
Summary of translational findings.

In conclusion, the present results suggest that slow dopamine neurotransmission-related signalling molecules compose a common, environmentally-responsive, cell type- and gender-dependant pathway associated with the genetic aetiology of addiction (Fig. 8). Early life experiences may modulate this vulnerability pathway.

## 5 Data sharing statement

GWAS data and RNA sequencing data used were both downloaded from public repository.

The GWAS data that support the findings of this study are available in dbGaP via the following access numbers. SUDs: phs000125.v1.p1 for Collaborative Study on the Genetics of Alcoholism (COGA), phs000092.v1.p1 for Study of Addiction: Genetics and Environment (SAGE), and phs000181.v1.p1 for the Australian twin-family study of alcohol use disorder (OZALC); PD: phg000126.v1.p1 for the Centre for Inherited Disease Research (CIDR), phs000089.v3.p2 for National Institute of Neurological Disorders and Stroke (NINDS) and phs000196.v2.p1 for the NeuroGenetics Research Consortium (NGRC).

The RNA sequencing data that support the findings of this study are available in GEO datasets: GSE108020, GSE112177, GSE67835, and GSE141463.

The other experiment data or codes for circular visualization that support the findings of this study are available from the corresponding authors upon request.

## Declaration of Competing Interest

The authors declare no competing interest.

## Appendix Supplementary materials

Image, application 1
