# Supplementary material for: Epistatic evidence for gender-dependant slow neurotransmission signalling in substance use disorders: PPP1R12B versus PPP1R1B
Source: eBioMedicine. 2020 Oct 21;61:103066. doi: 10.1016/j.ebiom.2020.103066 (PMC7581882; doi:10.1016/j.ebiom.2020.103066)
Supplement: Supplementary file 1 [file mmc1.pdf]

a

```
Rat Ppp1r12b    856 EAREARLASLTSRVEEDSSRDYKKLYESALTENQKLKTKLQEAQLELADIKAKLEKMAQQKQEKTSDRSSV 926
Human PPP1R12B  861 EAREARLATLTSRVEEDSNRDYKKLYESALTENQKLKTKLQEAQLELADIKSKLEKVAQQKQEKTSDRSSV 931
Rat Ppp1r12a    912 YSYLEERKPYGSRLEKDDSTDFKKLYEQILAENEKLKAQLHDTNMELTDLKLQLEKATQR-QERFADRSLL 982
```

b

```
Rat Ppp1r12b    1 MAELEHLGGKRAESARARRAEQLRRWRGSLTEQEPAERQGAGRQLQTRRGSPRVRFEDGAVFLAACSSGDTDEVKKLLAR
Human PPP1R12B  1 MAELEHLGGKRAESARMRRAEQLRRWRGSLTEQEPAERRGAGRQPLTRRGSPRVRFEDGAVFLAACSSGDTDEVRKLLAR
Rat Ppp1r12a    6 --------------AKQKRNEQLKRWIGSETDLEP---------PVVKRQKTKVKFDDGAVFLAACSSGDTDEVLKLLHR

Rat Ppp1r12b      GADINTVNVDGLTALHQACIDENLDMVKFLVENRANINQQDNEGWTPLHAAASCGYLNIAEYFISHGASVGIVNSEGEVP
Human PPP1R12B    GADINTVNVDGLTALHQACIDENLDMVKFLVENRANVNQQDNEGWTPLHAAASCGYLNIAEYFINHGASVGIVNSEGEVP
Rat Ppp1r12a      GADINYANVDGLTALHQACIDDNVDMVKFLVENGANINQPDNEGWIPLHAAASCGYLDIAEFLIGQGAHVGAVNSEGDTP

Rat Ppp1r12b      SDLAEEPAMKDLLLEQVKKQGVDLEQSRKEEEQQMLQDARQWLNSGKIEDVRQARSGATALHVAAAKGYSEVLRLLIQAG
Human PPP1R12B    SDLAEEPAMKDLLLEQVKKQGVDLEQSRKEEEQQMLQDARQWLNSGKIEDVRQARSGATALHVAAAKGYSEVLRLLIQAG
Rat Ppp1r12a      LDIAEEEAMEELLQNEVNRQGVDIEAARKEEERIMLRDARQWLNSGHISDVRHAKSGGTALHVAAAKGYTEVLKLLIQAG

Rat Ppp1r12b      YELNVQDHDGWTPLHAAAHWGVKEACSILAEALCDMDIRNKLGQTPFDVADEGLVEHLEMLQKKQDVLRSEKE-TRNKLI
Human PPP1R12B    YELNVQDYDGWTPLHAAAHWGVKEACSILAEALCDMDIRNKLGQTPFDVADEGLVEHLELLQKKQNVLRSEKE-TRNKLI
Rat Ppp1r12a      YDVNIKDYDGWTPLHAAAHWGKEEACRILVDNLCDMETVNKVGQTAFDVADEDILGYLEELQKKQNLLHSEKRDKKSPLI

Rat Ppp1r12b      ESDLN--SKFQSGLFKNKEKMLYEEEIPKSQETEEENKESSSSSSEEEEGEDEVSESETEKEADKKPEV 386
Human PPP1R12B    ESDLN--SKIQSGFFKNKEKMLYEEETPKSQEMEEENKESSSSSSEEEEGEDEASESETEKEAVLFWPF 386
Rat Ppp1r12a      ESTANMENNQPQKTFKNKETLIIEPE--KNASRIESL-E-QEKADEEEEGKKDESSCSSE-EDEEDDSE 366
```

c

```
                      forward primer                              reverse primer

Rat Ppp1r12b     3154 CTTCCTGTCCACCTCACTT 3172          3294 CCAGACCTGACCTCGTCTA  3276
                            |   |                                   |   ||    |||
Rat Ppp1r12a     2890 TAGAAAGAAGAATATCTGA 2908          3038 TTGTGTGTTTTCTGTCCTA  3020


Mouse Ppp1r12b   1535 CCTTAGGGATCGAGGTTCTT 1554         1709 AACAGCTGACTCTCTGTTCT 1690
                            | |      | |                     |   |  |||||||||||||
Mouse Ppp1r12a   1679 GAAGGAGAAAGACACTGCAG 1698         1865 ACTTGAAGACTCTCTGTTCT 1846
```

Supplementary Figure 1. Lack of PPP1R12B antigen homology to PPP1R12A. (**a-b**) Anti-C antibody's antigen peptide (corresponding to 71 amino acids of human PPP1R12B) (**a**) or Anti-N's antigen peptide (corresponding to 386 amino acids of human PPP1R12B) (**b**) has great identity (94%) to rat PPP1R12B (upper line) but low homolog to rat Ppp1r12a (35% identity in C-terminus; 59% identity in N-terminus) (bottle line). Numbers are corresponding amino acid positions in the proteins. (**c**) cDNA sequence alignments show the lack of primer sequence homology between *Ppp1r12b* and *Ppp1r12a*, for both rat (upper panel, 19-mer) and mouse (low panel, 20-mer). Shown are four *Ppp1r12b* sequences used by this study (also see Table 1): left for forward primer and right, reverse primer; numbers are corresponding base positions in the cDNA.

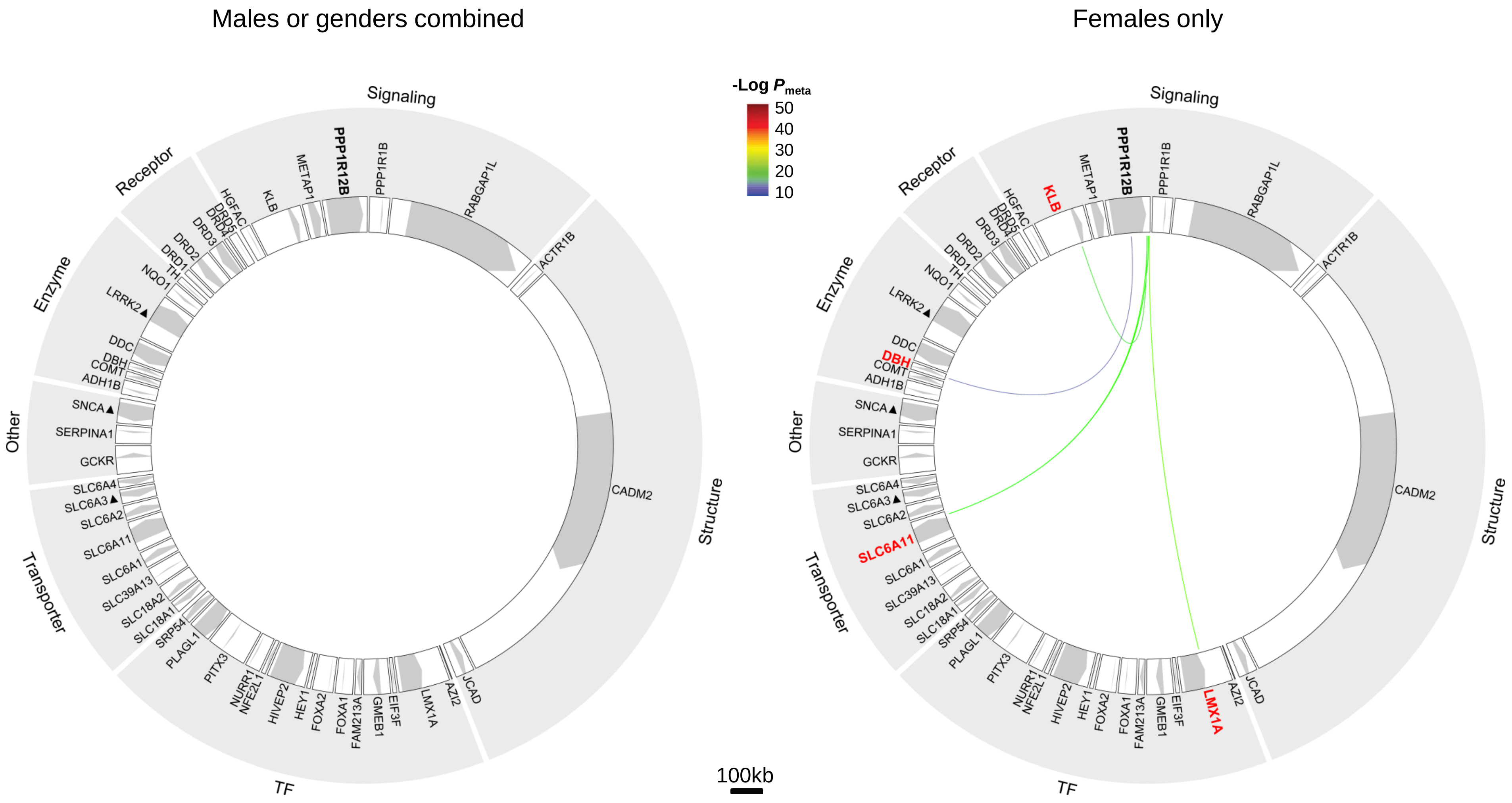

Supplementary Figure 2. Rare *PPP1R12B* (bold at top) interactions with other genes in PD, for females only *(right),* based on the 46 gene-network (see Figure 6 and Supplementary Table 1). Black triangle, known risk for PD. Interaction with LMX1A reached absolute GW significance after Bonferroni correction, referring to the thermometer bar on the upper middle; scale bar, 100 kb.

Supplementary Table 1. 46 genes in the *PPP1R12B* network wheel.

Gene name	System	order	from#	to#	Chr	Chr direction
ADH1B	Enzyme	1	100,200,153	100,292,187	4	minus strand
COMT	Enzyme	2	19,915,326	19,959,955	22	forward strand
DBH	Enzyme	3	136,482,068	136,528,721	9	forward strand
DDC	Enzyme	4	50,508,325	50,656,334	7	minus strand
LRRK2	Enzyme	5	40,500,195	40,799,277	12	forward strand
NQO1	Enzyme	6	69,700,201	69,798,954	16	minus strand
TH	Enzyme	7	2,171,601	2,213,694	11	minus strand
DRD1	Receptor	8	174,860,699	174,896,699	5	minus strand
DRD2	Receptor	9	113,249,725	113,399,491	11	minus strand
DRD3	Receptor	10	113,825,192	113,931,521	3	minus strand
DRD4	Receptor	11	619,671	641,191	11	forward strand
DRD5	Receptor	12	9,752,592	9,808,709	4	forward strand
HGFAC	Signaling	13	3,404,271	3,473,450	4	forward strand
KLB	Signaling	14	39,158,118	39,493,248	4	forward strand
METAP1	Signaling	15	99,883,133	99,999,848	4	forward strand
PPP1R12B	Signaling	16	202,293,916	202,585,582	1	forward strand
PPP1R1B	Signaling	17	37,700,199	37,832,386	17	forward strand
RABGAP1L	Signaling	18	174,002,465	174,997,750	1	forward strand
ACTR1B	Structure	19	98,242,427	98,318,644	2	minus strand
CADM2	Structure	20	84,007,790	86,992,225	3	forward strand
JCAD	Structure	21	30,273,174	30,396,395	10	minus strand
AZI2	TF	22	28,358,334	28,363,241	3	minus strand
EIF3F	TF	23	7,999,184	8,023,737	11	forward strand
FAM213A	TF	24	82,159,539	82,203,534	10	forward strand
FOXA1	TF	25	37,993,604	38,107,646	14	minus strand
FOXA2	TF	26	22,534,036	22,668,157	20	minus strand
GMEB1	TF	27	28,931,625	29,106,052	1	forward strand
HEY1	TF	28	80,660,497	80,683,321	8	minus strand
HIVEP2	TF	29	143,034,282	143,286,922	6	minus strand
LMX1A	TF	30	165,014,266	165,349,087	1	minus strand
NFE2L1	TF	31	46,120,325	46,147,807	17	forward strand
NURR1	TF	32	157,111,093	157,210,045	2	minus strand
PITX3	TF	33	103,837,118	104,098,949	10	minus strand
PLAGL1	TF	34	144,261,480	144,409,241	6	minus strand
SRP54	TF	35	35,434,291	35,501,800	14	forward strand
SLC18A1	Transporter	36	19,985,382	20,060,856	8	minus strand
SLC18A2	Transporter	37	118,981,071	119,091,767	10	forward strand
SLC39A13	Transporter	38	47,374,911	47,496,827	11	forward strand
SLC6A1	Transporter	39	11,004,257	11,107,643	3	forward strand
SLC6A11	Transporter	40	10,801,929	10,999,494	3	forward strand
SLC6A2	Transporter	41	55,670,106	55,765,906	16	forward strand
SLC6A3	Transporter	42	1,370,122	1,461,568	5	minus strand
SLC6A4	Transporter	43	28,515,188	28,574,177	17	minus strand
GCKR	Other	44	27,611,154	27,796,526	2	forward strand
SERPINA1	Other	45	94,770,681	94,889,702	14	minus strand
SNCA	Other	46	90,624,011	90,784,866	4	minus strand

# indicates the coordinates in chr, and define relative gene size in the wheel.

Supplementary Table 2. Significant SNP-SNP interactions in SUDs.

Males: 1-274; Females: 276-2119; see Table S1 for genes with Chr and BP information

No.	Chr1	SNP1	BP	Chr2	SNP2	BP2	*P* -value
1	1	rs35873832	202300127	3	rs188286851	86980758	4.74E-42
2	1	rs35873832	202300127	14	rs1956430	38088472	9.57E-32
3	1	rs17438379	202356935	14	rs1956430	38088472	2.02E-31
4	1	rs3767423	202417563	14	rs1956430	38088472	2.11E-31
5	1	rs17438212	202331510	14	rs1956430	38088472	2.66E-31
6	1	rs11583656	202370997	14	rs1956430	38088472	2.66E-31
7	1	rs4950779	202542202	3	rs34584686	85345197	3.17E-31
8	1	rs4950778	202431464	3	rs34584686	85345197	9.30E-31
9	1	rs12404552	202566780	6	rs597493	143060990	3.20E-28
10	1	rs4950778	202431464	6	rs6926546	144407790	5.53E-28
11	1	rs3767392	202547101	9	rs1611118	136504923	2.15E-27
12	1	rs4950779	202542202	6	rs6926546	144407790	2.29E-27
13	1	rs697459	202547922	2	rs6745653	98289997	3.24E-27
14	1	rs1046269	202559753	4	rs3775434	90702781	4.88E-27
15	1	rs17438212	202331510	3	rs188286851	86980758	5.74E-27
16	1	rs11583656	202370997	3	rs188286851	86980758	5.74E-27
17	1	rs697459	202547922	2	rs73959752	98242427	5.78E-27
18	1	rs697459	202547922	2	rs79616802	98277090	5.78E-27
19	1	rs697459	202547922	2	rs11886025	98279076	5.78E-27
20	1	rs697459	202547922	2	rs11886239	98279574	5.78E-27
21	1	rs697459	202547922	2	rs73959769	98288549	5.78E-27
22	1	rs17438379	202356935	3	rs188286851	86980758	6.21E-27
23	1	rs4950778	202431464	6	rs62427148	144400664	6.69E-27
24	1	rs697459	202547922	2	rs17022045	98261688	7.92E-27
25	1	rs12404552	202566780	3	rs11715683	85372128	1.21E-26
26	1	rs4950779	202542202	6	rs62427148	144400664	3.03E-26
27	1	rs1046269	202559753	4	rs3822089	90704011	3.23E-26
28	1	rs1046269	202559753	4	rs3822090	90704876	3.23E-26
29	1	rs4950778	202431464	6	rs55928616	144399821	4.02E-26
30	1	rs4950778	202431464	6	rs56282161	144398956	4.04E-26
31	1	rs4950778	202431464	6	rs62427144	144395736	4.61E-26
32	1	rs4950778	202431464	6	rs55847642	144392105	4.64E-26
33	1	rs1046269	202559753	4	rs6822088	90764310	5.73E-26
34	1	rs12033091	202563966	6	rs79924684	144315102	7.42E-26
35	1	rs12033827	202564040	6	rs79924684	144315102	7.42E-26
36	1	rs12033091	202563966	6	rs74748302	144315419	8.23E-26
37	1	rs12033827	202564040	6	rs74748302	144315419	8.23E-26
38	1	rs4950779	202542202	6	rs55928616	144399821	1.56E-25
39	1	rs4950779	202542202	6	rs56282161	144398956	1.56E-25
40	1	rs4950779	202542202	6	rs62427144	144395736	1.79E-25
41	1	rs4950779	202542202	6	rs55847642	144392105	1.80E-25
42	1	rs585658	202573179	11	rs4436578	113306765	2.17E-25
43	1	rs585658	202573179	11	rs4620755	113309619	2.74E-25
44	1	rs4950778	202431464	6	rs113618940	144403097	3.51E-25
45	1	rs1046269	202559753	4	rs3775439	90709741	5.05E-25
46	1	rs1046269	202559753	4	rs6830166	90744993	1.15E-24
47	1	rs4950779	202542202	6	rs113618940	144403097	1.32E-24

48	1	rs35873832	202300127	16	rs1566652	55731575	2.18E-24
49	1	rs17490864	202302232	3	rs1375559	85559006	2.60E-24
50	1	rs3767392	202547101	9	rs1611119	136505127	3.71E-24
51	1	rs17490864	202302232	3	rs67174538	85532862	4.04E-24
52	1	rs17490864	202302232	3	rs7626594	85533571	4.04E-24
53	1	rs17490864	202302232	3	rs67874563	85538693	4.04E-24
54	1	rs697459	202547922	2	rs11904110	98261802	4.60E-24
55	1	rs3767392	202547101	9	rs3739885	136509696	4.66E-24
56	1	rs3767392	202547101	9	rs3739886	136509679	4.70E-24
57	1	rs17490864	202302232	3	rs62252504	85581770	5.26E-24
58	1	rs17490864	202302232	3	rs62250717	85521990	5.33E-24
59	1	rs10800842	202570776	3	rs11715683	85372128	5.60E-24
60	1	rs17490864	202302232	3	rs9713905	85586748	6.66E-24
61	1	rs17490864	202302232	3	rs960986	85519305	6.72E-24
62	1	rs3767409	202479192	4	rs10018362	90703753	7.39E-24
63	1	rs17490864	202302232	3	rs66963835	85567382	7.98E-24
64	1	rs17490864	202302232	3	rs62250759	85569026	7.98E-24
65	1	rs17490864	202302232	3	rs2167048	85570393	7.98E-24
66	1	rs17490864	202302232	3	rs7624108	85527273	8.65E-24
67	1	rs3767409	202479192	4	rs10014396	90712629	8.90E-24
68	1	rs35873832	202300127	3	rs4435654	85682637	9.05E-24
69	1	rs17490864	202302232	3	rs68102233	85558538	9.76E-24
70	1	rs17490864	202302232	3	rs7637879	85533359	9.95E-24
71	1	rs17490864	202302232	3	rs17022915	85577613	1.05E-23
72	1	rs12404552	202566780	6	rs197492	143061160	1.28E-23
73	1	rs4950778	202431464	6	rs112340804	144403167	1.35E-23
74	1	rs17490864	202302232	3	rs62253963	85593642	1.37E-23
75	1	rs17490864	202302232	3	rs62250471	85612226	1.51E-23
76	1	rs17490864	202302232	3	rs62250755	85563061	1.53E-23
77	1	rs17490864	202302232	3	rs72615725	85599378	1.54E-23
78	1	rs17490864	202302232	3	rs4426693	85599384	1.54E-23
79	1	rs4950778	202431464	6	rs59906266	144405462	1.55E-23
80	1	rs17490864	202302232	3	rs17022886	85539308	1.60E-23
81	1	rs17490864	202302232	3	rs62252460	85569776	1.70E-23
82	1	rs17490864	202302232	3	rs4355295	85631163	1.99E-23
83	1	rs17490864	202302232	3	rs11920184	85555237	2.08E-23
84	1	rs17490864	202302232	3	rs1972992	85631329	2.19E-23
85	1	rs17490864	202302232	3	rs58834564	85550862	2.21E-23
86	1	rs17490864	202302232	3	rs10511084	85570561	2.24E-23
87	1	rs17490864	202302232	3	rs1449388	85577284	2.24E-23
88	1	rs17490864	202302232	3	rs1375567	85636711	2.24E-23
89	1	rs17490864	202302232	3	rs62250504	85634758	2.41E-23
90	1	rs17490864	202302232	3	rs55677942	85639341	2.57E-23
91	1	rs3767423	202417563	4	rs11944331	90690329	2.76E-23
92	1	rs12033091	202563966	6	rs3819812	144280799	3.38E-23
93	1	rs12033091	202563966	6	rs2328536	144281394	3.38E-23
94	1	rs12033827	202564040	6	rs3819812	144280799	3.38E-23
95	1	rs12033827	202564040	6	rs2328536	144281394	3.38E-23
96	1	rs17490864	202302232	3	rs62250724	85545232	3.39E-23
97	1	rs17490864	202302232	3	rs12636904	85546130	3.39E-23
98	1	rs17490864	202302232	3	rs17023016	85614074	3.39E-23
99	1	rs17490864	202302232	3	rs10511082	85615903	3.39E-23

100	1	rs35873832	202300127	3	rs10511074	85653433	3.67E-23
101	1	rs17490864	202302232	3	rs12632140	85546745	3.72E-23
102	1	rs3767423	202417563	4	rs6818319	90642868	3.74E-23
103	1	rs17490864	202302232	3	rs73137895	85622703	3.89E-23
104	1	rs4950779	202542202	6	rs112340804	144403167	4.43E-23
105	1	rs12033091	202563966	6	rs58195565	144280430	4.49E-23
106	1	rs12033827	202564040	6	rs58195565	144280430	4.49E-23
107	1	rs4950779	202542202	6	rs59906266	144405462	4.65E-23
108	1	rs3767423	202417563	4	rs6826785	90682474	4.70E-23
109	1	rs3767423	202417563	4	rs3857061	90686742	4.70E-23
110	1	rs17490864	202302232	3	rs12632674	85625019	5.11E-23
111	1	rs17490864	202302232	3	rs4508797	85634460	5.11E-23
112	1	rs12404552	202566780	3	rs9872078	85376492	5.15E-23
113	1	rs4950779	202542202	22	rs1109436	19953882	5.48E-23
114	1	rs12033091	202563966	6	rs9321952	144279640	5.63E-23
115	1	rs12033827	202564040	6	rs9321952	144279640	5.63E-23
116	1	rs2174696	165283470	1	rs10800842	202570776	5.65E-23
117	1	rs12404552	202566780	3	rs9831374	85370873	5.95E-23
118	1	rs3767423	202417563	4	rs7681312	90638527	7.72E-23
119	1	rs17490864	202302232	3	rs11927145	85602225	8.13E-23
120	1	rs12404552	202566780	3	rs11719276	85371838	8.19E-23
121	1	rs3767423	202417563	4	rs7661330	90663670	9.03E-23
122	1	rs3767423	202417563	4	rs3822086	90664794	9.03E-23
123	1	rs3767423	202417563	4	rs4088093	90671287	9.03E-23
124	1	rs3767423	202417563	4	rs3756054	90674451	9.03E-23
125	1	rs3767423	202417563	4	rs3857059	90675238	9.03E-23
126	1	rs12033091	202563966	6	rs2268447	144278095	9.18E-23
127	1	rs12033827	202564040	6	rs2268447	144278095	9.18E-23
128	1	rs3767423	202417563	4	rs7681815	90638614	9.97E-23
129	1	rs3767423	202417563	4	rs10516844	90639847	9.97E-23
130	1	rs3767423	202417563	4	rs61032876	90641403	9.97E-23
131	1	rs3767423	202417563	4	rs7655792	90642025	9.97E-23
132	1	rs3767423	202417563	4	rs10003708	90643051	9.97E-23
133	1	rs3767423	202417563	4	rs11945223	90643325	9.97E-23
134	1	rs3767423	202417563	4	rs3857051	90643697	9.97E-23
135	1	rs3767423	202417563	4	rs3857052	90643857	9.97E-23
136	1	rs3767423	202417563	4	rs7436973	90643921	9.97E-23
137	1	rs3767423	202417563	4	rs17016071	90644281	9.97E-23
138	1	rs3767423	202417563	4	rs8180209	90644454	9.97E-23
139	1	rs3767423	202417563	4	rs8180214	90644508	9.97E-23
140	1	rs3767423	202417563	4	rs7675290	90645003	9.97E-23
141	1	rs3767423	202417563	4	rs1045722	90645671	9.97E-23
142	1	rs3767423	202417563	4	rs3857053	90645674	9.97E-23
143	1	rs3767423	202417563	4	rs10033209	90653704	9.97E-23
144	1	rs3767423	202417563	4	rs3775422	90654664	9.97E-23
145	1	rs4950779	202542202	9	rs2283124	136515330	1.04E-22
146	1	rs3767423	202417563	4	rs3775423	90657491	1.07E-22
147	1	rs17490864	202302232	3	rs17022974	85597454	1.12E-22
148	1	rs17438379	202356935	3	rs4435654	85682637	1.60E-22
149	1	rs697459	202547922	4	rs11734036	90624787	1.62E-22
150	1	rs12404552	202566780	3	rs13078384	85264266	1.66E-22
151	1	rs3767409	202479192	16	rs1566652	55731575	1.85E-22

152	1	rs3767423	202417563	4	rs11931074	90639515	1.89E-22
153	1	rs12033091	202563966	3	rs11715683	85372128	1.91E-22
154	1	rs12033827	202564040	3	rs11715683	85372128	1.91E-22
155	1	rs17438212	202331510	3	rs4435654	85682637	2.46E-22
156	1	rs4950778	202431464	6	rs62427139	144390067	2.58E-22
157	1	rs12404552	202566780	3	rs13062591	85315738	4.06E-22
158	1	rs12404552	202566780	3	rs13087094	85353328	4.96E-22
159	1	rs4950778	202431464	22	rs1109436	19953882	5.70E-22
160	1	rs17438379	202356935	3	rs10511074	85653433	6.54E-22
161	1	rs4950778	202431464	9	rs2283124	136515330	6.64E-22
162	1	rs4950779	202542202	6	rs62427139	144390067	8.19E-22
163	1	rs17438212	202331510	3	rs10511074	85653433	1.02E-21
164	1	rs11583656	202370997	3	rs10511074	85653433	1.02E-21
165	1	rs12033091	202563966	6	rs198681	143197975	3.13E-21
166	1	rs2741853	202405671	5	rs2963260	1377021	3.97E-21
167	1	rs2741853	202405671	5	rs2937630	1376326	4.81E-21
168	1	rs697456	202514092	5	rs2963260	1377021	5.47E-21
169	1	rs705750	202531646	5	rs2963260	1377021	5.47E-21
170	1	rs147044345	174961477	1	rs17438212	202331510	5.89E-21
171	1	rs147044345	174961477	1	rs11583656	202370997	5.89E-21
172	1	rs147044345	174961477	1	rs35873832	202300127	6.38E-21
173	1	rs17438212	202331510	4	rs11944331	90690329	6.65E-21
174	1	rs11583656	202370997	4	rs11944331	90690329	6.65E-21
175	1	rs17438212	202331510	4	rs6826785	90682474	1.06E-20
176	1	rs17438212	202331510	4	rs3857061	90686742	1.06E-20
177	1	rs11583656	202370997	4	rs6826785	90682474	1.06E-20
178	1	rs11583656	202370997	4	rs3857061	90686742	1.06E-20
179	1	rs147044345	174961477	1	rs17438379	202356935	1.20E-20
180	1	rs1046269	202559753	6	rs12201410	143213127	1.36E-20
181	1	rs17438212	202331510	4	rs7681312	90638527	1.69E-20
182	1	rs11583656	202370997	4	rs7681312	90638527	1.69E-20
183	1	rs6427950	202472066	5	rs2292023	1461389	1.76E-20
184	1	rs17438212	202331510	4	rs7661330	90663670	1.94E-20
185	1	rs17438212	202331510	4	rs3822086	90664794	1.94E-20
186	1	rs17438212	202331510	4	rs4088093	90671287	1.94E-20
187	1	rs17438212	202331510	4	rs3756054	90674451	1.94E-20
188	1	rs17438212	202331510	4	rs3857059	90675238	1.94E-20
189	1	rs11583656	202370997	4	rs7661330	90663670	1.94E-20
190	1	rs11583656	202370997	4	rs3822086	90664794	1.94E-20
191	1	rs11583656	202370997	4	rs4088093	90671287	1.94E-20
192	1	rs11583656	202370997	4	rs3756054	90674451	1.94E-20
193	1	rs11583656	202370997	4	rs3857059	90675238	1.94E-20
194	1	rs35873832	202300127	16	rs5568	55730124	2.09E-20
195	1	rs17438212	202331510	4	rs7681815	90638614	2.11E-20
196	1	rs17438212	202331510	4	rs10516844	90639847	2.11E-20
197	1	rs17438212	202331510	4	rs61032876	90641403	2.11E-20
198	1	rs17438212	202331510	4	rs7655792	90642025	2.11E-20
199	1	rs17438212	202331510	4	rs10003708	90643051	2.11E-20
200	1	rs17438212	202331510	4	rs11945223	90643325	2.11E-20
201	1	rs17438212	202331510	4	rs3857051	90643697	2.11E-20
202	1	rs17438212	202331510	4	rs3857052	90643857	2.11E-20
203	1	rs17438212	202331510	4	rs7436973	90643921	2.11E-20

204	1	rs17438212	202331510	4	rs17016071	90644281	2.11E-20
205	1	rs17438212	202331510	4	rs8180209	90644454	2.11E-20
206	1	rs17438212	202331510	4	rs8180214	90644508	2.11E-20
207	1	rs17438212	202331510	4	rs7675290	90645003	2.11E-20
208	1	rs17438212	202331510	4	rs1045722	90645671	2.11E-20
209	1	rs17438212	202331510	4	rs3857053	90645674	2.11E-20
210	1	rs17438212	202331510	4	rs10033209	90653704	2.11E-20
211	1	rs17438212	202331510	4	rs3775422	90654664	2.11E-20
212	1	rs11583656	202370997	4	rs7681815	90638614	2.11E-20
213	1	rs11583656	202370997	4	rs10516844	90639847	2.11E-20
214	1	rs11583656	202370997	4	rs61032876	90641403	2.11E-20
215	1	rs11583656	202370997	4	rs7655792	90642025	2.11E-20
216	1	rs11583656	202370997	4	rs10003708	90643051	2.11E-20
217	1	rs11583656	202370997	4	rs11945223	90643325	2.11E-20
218	1	rs11583656	202370997	4	rs3857051	90643697	2.11E-20
219	1	rs11583656	202370997	4	rs3857052	90643857	2.11E-20
220	1	rs11583656	202370997	4	rs7436973	90643921	2.11E-20
221	1	rs11583656	202370997	4	rs17016071	90644281	2.11E-20
222	1	rs11583656	202370997	4	rs8180209	90644454	2.11E-20
223	1	rs11583656	202370997	4	rs8180214	90644508	2.11E-20
224	1	rs11583656	202370997	4	rs7675290	90645003	2.11E-20
225	1	rs11583656	202370997	4	rs1045722	90645671	2.11E-20
226	1	rs11583656	202370997	4	rs3857053	90645674	2.11E-20
227	1	rs11583656	202370997	4	rs10033209	90653704	2.11E-20
228	1	rs11583656	202370997	4	rs3775422	90654664	2.11E-20
229	1	rs930734	202318316	5	rs2292023	1461389	2.32E-20
230	1	rs10920392	202321123	5	rs2292023	1461389	2.32E-20
231	1	rs6658708	202322113	5	rs2292023	1461389	2.32E-20
232	1	rs2361453	202336314	5	rs2292023	1461389	2.32E-20
233	1	rs17438212	202331510	4	rs3775423	90657491	2.37E-20
234	1	rs11583656	202370997	4	rs3775423	90657491	2.37E-20
235	1	rs697456	202514092	5	rs2963259	1377172	2.54E-20
236	1	rs705750	202531646	5	rs2963259	1377172	2.54E-20
237	1	rs12033091	202563966	6	rs198654	143170004	2.83E-20
238	1	rs12033091	202563966	6	rs185588	143173998	2.83E-20
239	1	rs697456	202514092	5	rs2927670	1377075	2.87E-20
240	1	rs705750	202531646	5	rs2927670	1377075	2.87E-20
241	1	rs697456	202514092	5	rs2937630	1376326	3.24E-20
242	1	rs705750	202531646	5	rs2937630	1376326	3.24E-20
243	1	rs17438379	202356935	4	rs11944331	90690329	3.73E-20
244	1	rs17438212	202331510	4	rs11931074	90639515	3.82E-20
245	1	rs11583656	202370997	4	rs11931074	90639515	3.82E-20
246	1	rs17438379	202356935	4	rs6826785	90682474	5.89E-20
247	1	rs17438379	202356935	4	rs3857061	90686742	5.89E-20
248	1	rs3767392	202547101	5	rs265978	174863905	2.10E-19
249	1	rs10465591	202308344	5	rs2292023	1461389	7.19E-19
250	1	rs3767409	202479192	4	rs6830166	90744993	7.21E-18
251	1	rs17438212	202331510	16	rs1566652	55731575	8.22E-18
252	1	rs11583656	202370997	16	rs1566652	55731575	8.22E-18
253	1	rs17438379	202356935	16	rs1566652	55731575	6.04E-17
254	1	rs3767409	202479192	4	rs3775439	90709741	6.77E-17
255	1	rs4950778	202431464	9	rs61149283	136519258	6.91E-17

256	1	rs3767409	202479192	4	rs6822088	90764310	9.10E-17
257	1	rs2741849	202373394	9	rs6271	136522274	1.41E-16
258	1	rs3767392	202547101	5	rs835616	174863690	1.80E-16
259	1	rs697459	202547922	2	rs113537081	98302136	1.89E-16
260	1	rs697459	202547922	2	rs6543021	98318644	1.89E-16
261	1	rs3767409	202479192	4	rs3822089	90704011	2.52E-16
262	1	rs3767409	202479192	4	rs3822090	90704876	2.52E-16
263	1	rs4950779	202542202	9	rs61149283	136519258	7.86E-16
264	1	rs4950778	202431464	9	rs59164034	136519639	8.44E-16
265	1	rs1046269	202559753	6	rs9496470	143184472	1.64E-15
266	1	rs697459	202547922	10	rs2803804	119091401	2.14E-15
267	1	rs35873832	202300127	16	rs47958	55726462	2.96E-15
268	1	rs12404552	202566780	6	rs9399469	144318529	3.15E-15
269	1	rs3767392	202547101	9	rs1611126	136509448	3.22E-15
270	1	rs1046269	202559753	6	rs13211716	143203528	3.88E-15
271	1	rs4950779	202542202	9	rs59164034	136519639	7.17E-15
272	1	rs1046269	202559753	6	rs9496471	143184677	1.22E-14
273	1	rs12033091	202563966	6	rs9399469	144318529	2.83E-14
274	1	rs12033827	202564040	6	rs9399469	144318529	2.83E-14
	**Females**						
276	1	rs75461537	174869593	1	rs17492329	202461103	5.794E-41
277	1	rs77551912	174873951	1	rs17492329	202461103	1.282E-40
278	1	rs6700434	174868328	1	rs17492329	202461103	3.513E-40
279	1	rs76881580	174855306	1	rs17492329	202461103	6.279E-40
280	1	rs3753562	174923567	1	rs17492329	202461103	2.682E-39
281	1	rs76828045	174847442	1	rs17492329	202461103	4.196E-39
282	1	rs147728966	174848270	1	rs17492329	202461103	4.196E-39
283	1	rs80134637	174749551	1	rs17492329	202461103	1.308E-38
284	1	rs59436654	174746513	1	rs17492329	202461103	1.741E-38
285	1	rs58762254	174780210	1	rs17492329	202461103	2.547E-38
286	1	rs61028529	174787899	1	rs17492329	202461103	2.547E-38
287	1	rs79203634	174793526	1	rs17492329	202461103	2.547E-38
288	1	rs74524401	174795281	1	rs17492329	202461103	2.547E-38
289	1	rs57053120	174830490	1	rs17492329	202461103	2.552E-38
290	1	rs77528209	174881910	1	rs17492329	202461103	2.672E-38
291	1	rs74649124	174760589	1	rs17492329	202461103	3.422E-38
292	1	rs79258314	174760696	1	rs17492329	202461103	3.422E-38
293	1	rs76616345	174766778	1	rs17492329	202461103	1.673E-37
294	1	rs41498747	174914122	1	rs17492329	202461103	1.494E-36
295	1	rs80324733	174776328	1	rs17492329	202461103	2.949E-36
296	1	rs4565668	174915927	1	rs17492329	202461103	4.123E-36
297	1	rs12137988	202317696	3	rs111466137	113841877	9.946E-31
298	1	rs141729655	174432399	1	rs2292489	202536848	5.854E-30
299	1	rs79077452	174435344	1	rs2292489	202536848	5.854E-30
300	1	rs141729655	174432399	1	rs930734	202318316	9.729E-30
301	1	rs79077452	174435344	1	rs930734	202318316	9.729E-30
302	1	rs141729655	174432399	1	rs2361453	202336314	1.084E-29
303	1	rs79077452	174435344	1	rs2361453	202336314	1.084E-29
304	1	rs141729655	174432399	1	rs6427950	202472066	1.173E-29
305	1	rs141729655	174432399	1	rs3753908	202511295	1.173E-29
306	1	rs141729655	174432399	1	rs2249811	202531741	1.173E-29
307	1	rs141729655	174432399	1	rs925532	202533880	1.173E-29

308	1	rs141729655	174432399	1	rs925533	202534108	1.173E-29
309	1	rs141729655	174432399	1	rs3767397	202534389	1.173E-29
310	1	rs141729655	174432399	1	rs3767395	202536140	1.173E-29
311	1	rs79077452	174435344	1	rs6427950	202472066	1.173E-29
312	1	rs79077452	174435344	1	rs3753908	202511295	1.173E-29
313	1	rs79077452	174435344	1	rs2249811	202531741	1.173E-29
314	1	rs79077452	174435344	1	rs925532	202533880	1.173E-29
315	1	rs79077452	174435344	1	rs925533	202534108	1.173E-29
316	1	rs79077452	174435344	1	rs3767397	202534389	1.173E-29
317	1	rs79077452	174435344	1	rs3767395	202536140	1.173E-29
318	1	rs10489261	174440683	1	rs2292489	202536848	1.403E-29
319	1	rs141729655	174432399	1	rs1890875	202531366	1.459E-29
320	1	rs79077452	174435344	1	rs1890875	202531366	1.459E-29
321	1	rs141729655	174432399	1	rs10800836	202410429	1.533E-29
322	1	rs141729655	174432399	1	rs3767425	202413840	1.533E-29
323	1	rs141729655	174432399	1	rs3767421	202432160	1.533E-29
324	1	rs79077452	174435344	1	rs10800836	202410429	1.533E-29
325	1	rs79077452	174435344	1	rs3767425	202413840	1.533E-29
326	1	rs79077452	174435344	1	rs3767421	202432160	1.533E-29
327	1	rs141729655	174432399	1	rs10920392	202321123	1.65E-29
328	1	rs141729655	174432399	1	rs6658708	202322113	1.65E-29
329	1	rs79077452	174435344	1	rs10920392	202321123	1.65E-29
330	1	rs79077452	174435344	1	rs6658708	202322113	1.65E-29
331	1	rs141729655	174432399	1	rs10159280	202394287	2.051E-29
332	1	rs79077452	174435344	1	rs10159280	202394287	2.051E-29
333	1	rs10489261	174440683	1	rs930734	202318316	2.326E-29
334	1	rs10489261	174440683	1	rs2361453	202336314	2.586E-29
335	1	rs58762254	174780210	1	rs2292489	202536848	2.656E-29
336	1	rs61028529	174787899	1	rs2292489	202536848	2.656E-29
337	1	rs79203634	174793526	1	rs2292489	202536848	2.656E-29
338	1	rs74524401	174795281	1	rs2292489	202536848	2.656E-29
339	1	rs10489261	174440683	1	rs6427950	202472066	2.8E-29
340	1	rs10489261	174440683	1	rs3753908	202511295	2.8E-29
341	1	rs10489261	174440683	1	rs2249811	202531741	2.8E-29
342	1	rs10489261	174440683	1	rs925532	202533880	2.8E-29
343	1	rs10489261	174440683	1	rs925533	202534108	2.8E-29
344	1	rs10489261	174440683	1	rs3767397	202534389	2.8E-29
345	1	rs10489261	174440683	1	rs3767395	202536140	2.8E-29
346	1	rs59436654	174746513	1	rs2292489	202536848	3.14E-29
347	1	rs80134637	174749551	1	rs2292489	202536848	3.302E-29
348	1	rs10489261	174440683	1	rs1890875	202531366	3.481E-29
349	1	rs10489261	174440683	1	rs10800836	202410429	3.657E-29
350	1	rs10489261	174440683	1	rs3767425	202413840	3.657E-29
351	1	rs10489261	174440683	1	rs3767421	202432160	3.657E-29
352	1	rs10489261	174440683	1	rs10920392	202321123	3.929E-29
353	1	rs10489261	174440683	1	rs6658708	202322113	3.929E-29
354	1	rs57053120	174830490	1	rs2292489	202536848	3.954E-29
355	1	rs58762254	174780210	1	rs930734	202318316	4.208E-29
356	1	rs61028529	174787899	1	rs930734	202318316	4.208E-29
357	1	rs79203634	174793526	1	rs930734	202318316	4.208E-29
358	1	rs74524401	174795281	1	rs930734	202318316	4.208E-29
359	1	rs58762254	174780210	1	rs2361453	202336314	4.654E-29

360	1	rs61028529	174787899	1	rs2361453	202336314	4.654E-29
361	1	rs79203634	174793526	1	rs2361453	202336314	4.654E-29
362	1	rs74524401	174795281	1	rs2361453	202336314	4.654E-29
363	1	rs10489261	174440683	1	rs10159280	202394287	4.879E-29
364	1	rs59436654	174746513	1	rs930734	202318316	4.994E-29
365	1	rs58762254	174780210	1	rs6427950	202472066	5.052E-29
366	1	rs58762254	174780210	1	rs3753908	202511295	5.052E-29
367	1	rs58762254	174780210	1	rs2249811	202531741	5.052E-29
368	1	rs58762254	174780210	1	rs925532	202533880	5.052E-29
369	1	rs58762254	174780210	1	rs925533	202534108	5.052E-29
370	1	rs58762254	174780210	1	rs3767397	202534389	5.052E-29
371	1	rs58762254	174780210	1	rs3767395	202536140	5.052E-29
372	1	rs61028529	174787899	1	rs6427950	202472066	5.052E-29
373	1	rs61028529	174787899	1	rs3753908	202511295	5.052E-29
374	1	rs61028529	174787899	1	rs2249811	202531741	5.052E-29
375	1	rs61028529	174787899	1	rs925532	202533880	5.052E-29
376	1	rs61028529	174787899	1	rs925533	202534108	5.052E-29
377	1	rs61028529	174787899	1	rs3767397	202534389	5.052E-29
378	1	rs61028529	174787899	1	rs3767395	202536140	5.052E-29
379	1	rs79203634	174793526	1	rs6427950	202472066	5.052E-29
380	1	rs79203634	174793526	1	rs3753908	202511295	5.052E-29
381	1	rs79203634	174793526	1	rs2249811	202531741	5.052E-29
382	1	rs79203634	174793526	1	rs925532	202533880	5.052E-29
383	1	rs79203634	174793526	1	rs925533	202534108	5.052E-29
384	1	rs79203634	174793526	1	rs3767397	202534389	5.052E-29
385	1	rs79203634	174793526	1	rs3767395	202536140	5.052E-29
386	1	rs74524401	174795281	1	rs6427950	202472066	5.052E-29
387	1	rs74524401	174795281	1	rs3753908	202511295	5.052E-29
388	1	rs74524401	174795281	1	rs2249811	202531741	5.052E-29
389	1	rs74524401	174795281	1	rs925532	202533880	5.052E-29
390	1	rs74524401	174795281	1	rs925533	202534108	5.052E-29
391	1	rs74524401	174795281	1	rs3767397	202534389	5.052E-29
392	1	rs74524401	174795281	1	rs3767395	202536140	5.052E-29
393	1	rs80134637	174749551	1	rs930734	202318316	5.239E-29
394	1	rs141729655	174432399	1	rs10465591	202308344	5.274E-29
395	1	rs79077452	174435344	1	rs10465591	202308344	5.274E-29
396	1	rs59436654	174746513	1	rs2361453	202336314	5.504E-29
397	1	rs80134637	174749551	1	rs2361453	202336314	5.786E-29
398	1	rs74649124	174760589	1	rs2292489	202536848	5.887E-29
399	1	rs79258314	174760696	1	rs2292489	202536848	5.887E-29
400	1	rs59436654	174746513	1	rs6427950	202472066	5.975E-29
401	1	rs59436654	174746513	1	rs3753908	202511295	5.975E-29
402	1	rs59436654	174746513	1	rs2249811	202531741	5.975E-29
403	1	rs59436654	174746513	1	rs925532	202533880	5.975E-29
404	1	rs59436654	174746513	1	rs925533	202534108	5.975E-29
405	1	rs59436654	174746513	1	rs3767397	202534389	5.975E-29
406	1	rs59436654	174746513	1	rs3767395	202536140	5.975E-29
407	1	rs58762254	174780210	1	rs1890875	202531366	6.179E-29
408	1	rs61028529	174787899	1	rs1890875	202531366	6.179E-29
409	1	rs79203634	174793526	1	rs1890875	202531366	6.179E-29
410	1	rs74524401	174795281	1	rs1890875	202531366	6.179E-29
411	1	rs57053120	174830490	1	rs930734	202318316	6.253E-29

412	1	rs80134637	174749551	1	rs6427950	202472066	6.277E-29
413	1	rs80134637	174749551	1	rs3753908	202511295	6.277E-29
414	1	rs80134637	174749551	1	rs2249811	202531741	6.277E-29
415	1	rs80134637	174749551	1	rs925532	202533880	6.277E-29
416	1	rs80134637	174749551	1	rs925533	202534108	6.277E-29
417	1	rs80134637	174749551	1	rs3767397	202534389	6.277E-29
418	1	rs80134637	174749551	1	rs3767395	202536140	6.277E-29
419	1	rs58762254	174780210	1	rs10800836	202410429	6.458E-29
420	1	rs58762254	174780210	1	rs3767425	202413840	6.458E-29
421	1	rs58762254	174780210	1	rs3767421	202432160	6.458E-29
422	1	rs61028529	174787899	1	rs10800836	202410429	6.458E-29
423	1	rs61028529	174787899	1	rs3767425	202413840	6.458E-29
424	1	rs61028529	174787899	1	rs3767421	202432160	6.458E-29
425	1	rs79203634	174793526	1	rs10800836	202410429	6.458E-29
426	1	rs79203634	174793526	1	rs3767425	202413840	6.458E-29
427	1	rs79203634	174793526	1	rs3767421	202432160	6.458E-29
428	1	rs74524401	174795281	1	rs10800836	202410429	6.458E-29
429	1	rs74524401	174795281	1	rs3767425	202413840	6.458E-29
430	1	rs74524401	174795281	1	rs3767421	202432160	6.458E-29
431	1	rs58762254	174780210	1	rs10920392	202321123	6.879E-29
432	1	rs58762254	174780210	1	rs6658708	202322113	6.879E-29
433	1	rs61028529	174787899	1	rs10920392	202321123	6.879E-29
434	1	rs61028529	174787899	1	rs6658708	202322113	6.879E-29
435	1	rs79203634	174793526	1	rs10920392	202321123	6.879E-29
436	1	rs79203634	174793526	1	rs6658708	202322113	6.879E-29
437	1	rs74524401	174795281	1	rs10920392	202321123	6.879E-29
438	1	rs74524401	174795281	1	rs6658708	202322113	6.879E-29
439	1	rs57053120	174830490	1	rs2361453	202336314	6.904E-29
440	1	rs6692753	174737457	1	rs2292489	202536848	7.275E-29
441	1	rs59436654	174746513	1	rs1890875	202531366	7.308E-29
442	1	rs1040010	202560412	5	rs265973	174860699	7.485E-29
443	1	rs57053120	174830490	1	rs6427950	202472066	7.506E-29
444	1	rs57053120	174830490	1	rs3753908	202511295	7.506E-29
445	1	rs57053120	174830490	1	rs2249811	202531741	7.506E-29
446	1	rs57053120	174830490	1	rs925532	202533880	7.506E-29
447	1	rs57053120	174830490	1	rs925533	202534108	7.506E-29
448	1	rs57053120	174830490	1	rs3767397	202534389	7.506E-29
449	1	rs57053120	174830490	1	rs3767395	202536140	7.506E-29
450	1	rs59436654	174746513	1	rs10800836	202410429	7.639E-29
451	1	rs59436654	174746513	1	rs3767425	202413840	7.639E-29
452	1	rs59436654	174746513	1	rs3767421	202432160	7.64E-29
453	1	rs80134637	174749551	1	rs1890875	202531366	7.677E-29
454	1	rs80134637	174749551	1	rs10800836	202410429	8.025E-29
455	1	rs80134637	174749551	1	rs3767425	202413840	8.025E-29
456	1	rs80134637	174749551	1	rs3767421	202432160	8.026E-29
457	1	rs59436654	174746513	1	rs10920392	202321123	8.134E-29
458	1	rs59436654	174746513	1	rs6658708	202322113	8.134E-29
459	1	rs58762254	174780210	1	rs10159280	202394287	8.406E-29
460	1	rs61028529	174787899	1	rs10159280	202394287	8.406E-29
461	1	rs79203634	174793526	1	rs10159280	202394287	8.406E-29
462	1	rs74524401	174795281	1	rs10159280	202394287	8.406E-29
463	1	rs80134637	174749551	1	rs10920392	202321123	8.547E-29

464	1	rs80134637	174749551	1	rs6658708	202322113	8.547E-29
465	1	rs57053120	174830490	1	rs1890875	202531366	9.183E-29
466	1	rs74649124	174760589	1	rs930734	202318316	9.286E-29
467	1	rs79258314	174760696	1	rs930734	202318316	9.286E-29
468	1	rs57053120	174830490	1	rs10800836	202410429	9.583E-29
469	1	rs57053120	174830490	1	rs3767425	202413840	9.583E-29
470	1	rs57053120	174830490	1	rs3767421	202432160	9.583E-29
471	1	rs59436654	174746513	1	rs10159280	202394287	9.942E-29
472	1	rs57053120	174830490	1	rs10920392	202321123	1.02E-28
473	1	rs57053120	174830490	1	rs6658708	202322113	1.02E-28
474	1	rs74649124	174760589	1	rs2361453	202336314	1.026E-28
475	1	rs79258314	174760696	1	rs2361453	202336314	1.026E-28
476	1	rs80134637	174749551	1	rs10159280	202394287	1.045E-28
477	1	rs74649124	174760589	1	rs6427950	202472066	1.12E-28
478	1	rs74649124	174760589	1	rs3753908	202511295	1.12E-28
479	1	rs74649124	174760589	1	rs2249811	202531741	1.12E-28
480	1	rs74649124	174760589	1	rs925532	202533880	1.12E-28
481	1	rs74649124	174760589	1	rs925533	202534108	1.12E-28
482	1	rs74649124	174760589	1	rs3767397	202534389	1.12E-28
483	1	rs74649124	174760589	1	rs3767395	202536140	1.12E-28
484	1	rs79258314	174760696	1	rs6427950	202472066	1.12E-28
485	1	rs79258314	174760696	1	rs3753908	202511295	1.12E-28
486	1	rs79258314	174760696	1	rs2249811	202531741	1.12E-28
487	1	rs79258314	174760696	1	rs925532	202533880	1.12E-28
488	1	rs79258314	174760696	1	rs925533	202534108	1.12E-28
489	1	rs79258314	174760696	1	rs3767397	202534389	1.12E-28
490	1	rs79258314	174760696	1	rs3767395	202536140	1.12E-28
491	1	rs6692753	174737457	1	rs930734	202318316	1.187E-28
492	1	rs57053120	174830490	1	rs10159280	202394287	1.247E-28
493	1	rs10489261	174440683	1	rs10465591	202308344	1.249E-28
494	1	rs76828045	174847442	1	rs2292489	202536848	1.261E-28
495	1	rs147728966	174848270	1	rs2292489	202536848	1.261E-28
496	1	rs6692753	174737457	1	rs2361453	202336314	1.317E-28
497	1	rs74649124	174760589	1	rs1890875	202531366	1.368E-28
498	1	rs79258314	174760696	1	rs1890875	202531366	1.368E-28
499	1	rs74649124	174760589	1	rs10800836	202410429	1.429E-28
500	1	rs74649124	174760589	1	rs3767425	202413840	1.429E-28
501	1	rs74649124	174760589	1	rs3767421	202432160	1.429E-28
502	1	rs79258314	174760696	1	rs10800836	202410429	1.429E-28
503	1	rs79258314	174760696	1	rs3767425	202413840	1.429E-28
504	1	rs79258314	174760696	1	rs3767421	202432160	1.429E-28
505	1	rs6692753	174737457	1	rs6427950	202472066	1.436E-28
506	1	rs6692753	174737457	1	rs3753908	202511295	1.436E-28
507	1	rs6692753	174737457	1	rs2249811	202531741	1.436E-28
508	1	rs6692753	174737457	1	rs925532	202533880	1.436E-28
509	1	rs6692753	174737457	1	rs925533	202534108	1.436E-28
510	1	rs6692753	174737457	1	rs3767397	202534389	1.436E-28
511	1	rs6692753	174737457	1	rs3767395	202536140	1.436E-28
512	1	rs4950858	202552100	5	rs265973	174860699	1.456E-28
513	1	rs74649124	174760589	1	rs10920392	202321123	1.516E-28
514	1	rs74649124	174760589	1	rs6658708	202322113	1.516E-28
515	1	rs79258314	174760696	1	rs10920392	202321123	1.516E-28

516	1	rs79258314	174760696	1	rs6658708	202322113	1.516E-28
517	1	rs747640	202551071	4	rs6853599	3445610	1.575E-28
518	1	rs6692753	174737457	1	rs1890875	202531366	1.774E-28
519	1	rs74649124	174760589	1	rs10159280	202394287	1.85E-28
520	1	rs79258314	174760696	1	rs10159280	202394287	1.85E-28
521	1	rs6692753	174737457	1	rs10800836	202410429	1.861E-28
522	1	rs6692753	174737457	1	rs3767425	202413840	1.861E-28
523	1	rs6692753	174737457	1	rs3767421	202432160	1.861E-28
524	1	rs76828045	174847442	1	rs930734	202318316	1.981E-28
525	1	rs147728966	174848270	1	rs930734	202318316	1.981E-28
526	1	rs6692753	174737457	1	rs10920392	202321123	1.987E-28
527	1	rs6692753	174737457	1	rs6658708	202322113	1.987E-28
528	1	rs58762254	174780210	1	rs10465591	202308344	2.012E-28
529	1	rs61028529	174787899	1	rs10465591	202308344	2.012E-28
530	1	rs79203634	174793526	1	rs10465591	202308344	2.012E-28
531	1	rs74524401	174795281	1	rs10465591	202308344	2.012E-28
532	1	rs76828045	174847442	1	rs2361453	202336314	2.192E-28
533	1	rs147728966	174848270	1	rs2361453	202336314	2.192E-28
534	1	rs76828045	174847442	1	rs6427950	202472066	2.38E-28
535	1	rs76828045	174847442	1	rs3753908	202511295	2.38E-28
536	1	rs76828045	174847442	1	rs2249811	202531741	2.38E-28
537	1	rs76828045	174847442	1	rs925532	202533880	2.38E-28
538	1	rs76828045	174847442	1	rs925533	202534108	2.38E-28
539	1	rs76828045	174847442	1	rs3767397	202534389	2.38E-28
540	1	rs76828045	174847442	1	rs3767395	202536140	2.38E-28
541	1	rs147728966	174848270	1	rs6427950	202472066	2.38E-28
542	1	rs147728966	174848270	1	rs3753908	202511295	2.38E-28
543	1	rs147728966	174848270	1	rs2249811	202531741	2.38E-28
544	1	rs147728966	174848270	1	rs925532	202533880	2.38E-28
545	1	rs147728966	174848270	1	rs925533	202534108	2.38E-28
546	1	rs147728966	174848270	1	rs3767397	202534389	2.38E-28
547	1	rs147728966	174848270	1	rs3767395	202536140	2.38E-28
548	1	rs59436654	174746513	1	rs10465591	202308344	2.381E-28
549	1	rs6692753	174737457	1	rs10159280	202394287	2.453E-28
550	1	rs80134637	174749551	1	rs10465591	202308344	2.499E-28
551	1	rs60885521	174591547	1	rs2292489	202536848	2.652E-28
552	1	rs76828045	174847442	1	rs1890875	202531366	2.907E-28
553	1	rs147728966	174848270	1	rs1890875	202531366	2.907E-28
554	1	rs57053120	174830490	1	rs10465591	202308344	2.975E-28
555	1	rs76828045	174847442	1	rs10800836	202410429	3.034E-28
556	1	rs76828045	174847442	1	rs3767425	202413840	3.034E-28
557	1	rs76828045	174847442	1	rs3767421	202432160	3.034E-28
558	1	rs147728966	174848270	1	rs10800836	202410429	3.034E-28
559	1	rs147728966	174848270	1	rs3767425	202413840	3.034E-28
560	1	rs147728966	174848270	1	rs3767421	202432160	3.034E-28
561	1	rs76828045	174847442	1	rs10920392	202321123	3.233E-28
562	1	rs76828045	174847442	1	rs6658708	202322113	3.233E-28
563	1	rs147728966	174848270	1	rs10920392	202321123	3.233E-28
564	1	rs147728966	174848270	1	rs6658708	202322113	3.233E-28
565	1	rs76828045	174847442	1	rs10159280	202394287	3.947E-28
566	1	rs147728966	174848270	1	rs10159280	202394287	3.947E-28
567	1	rs60885521	174591547	1	rs930734	202318316	4.31E-28

568	1	rs74649124	174760589	1	rs10465591	202308344	4.419E-28
569	1	rs79258314	174760696	1	rs10465591	202308344	4.419E-28
570	1	rs60885521	174591547	1	rs2361453	202336314	4.773E-28
571	1	rs60885521	174591547	1	rs6427950	202472066	5.169E-28
572	1	rs60885521	174591547	1	rs3753908	202511295	5.169E-28
573	1	rs60885521	174591547	1	rs2249811	202531741	5.169E-28
574	1	rs60885521	174591547	1	rs925532	202533880	5.169E-28
575	1	rs60885521	174591547	1	rs925533	202534108	5.169E-28
576	1	rs60885521	174591547	1	rs3767397	202534389	5.169E-28
577	1	rs60885521	174591547	1	rs3767395	202536140	5.169E-28
578	1	rs60723087	174655617	1	rs2292489	202536848	5.267E-28
579	1	rs75062924	174664341	1	rs2292489	202536848	5.267E-28
580	1	rs74739388	174666752	1	rs2292489	202536848	5.267E-28
581	1	rs74225917	174686241	1	rs2292489	202536848	5.267E-28
582	1	rs59354288	174687003	1	rs2292489	202536848	5.267E-28
583	1	rs60665754	174687287	1	rs2292489	202536848	5.267E-28
584	1	rs76014701	174690404	1	rs2292489	202536848	5.267E-28
585	1	rs74365829	174690558	1	rs2292489	202536848	5.267E-28
586	1	rs6692753	174737457	1	rs10465591	202308344	6.166E-28
587	1	rs78199276	174706357	1	rs2292489	202536848	6.223E-28
588	1	rs6695956	174711947	1	rs2292489	202536848	6.223E-28
589	1	rs77727672	174718575	1	rs2292489	202536848	6.223E-28
590	1	rs79388999	174724332	1	rs2292489	202536848	6.223E-28
591	1	rs61329817	174724485	1	rs2292489	202536848	6.223E-28
592	1	rs60885521	174591547	1	rs1890875	202531366	6.375E-28
593	1	rs60885521	174591547	1	rs10800836	202410429	6.684E-28
594	1	rs60885521	174591547	1	rs3767425	202413840	6.684E-28
595	1	rs60885521	174591547	1	rs3767421	202432160	6.684E-28
596	1	rs60885521	174591547	1	rs10920392	202321123	7.154E-28
597	1	rs60885521	174591547	1	rs6658708	202322113	7.154E-28
598	1	rs4950859	202552839	4	rs6853599	3445610	7.424E-28
599	1	rs76616345	174766778	1	rs2292489	202536848	7.492E-28
600	1	rs7543502	174653701	1	rs2292489	202536848	7.745E-28
601	1	rs60723087	174655617	1	rs930734	202318316	8.574E-28
602	1	rs75062924	174664341	1	rs930734	202318316	8.574E-28
603	1	rs74739388	174666752	1	rs930734	202318316	8.574E-28
604	1	rs74225917	174686241	1	rs930734	202318316	8.574E-28
605	1	rs59354288	174687003	1	rs930734	202318316	8.574E-28
606	1	rs60665754	174687287	1	rs930734	202318316	8.574E-28
607	1	rs76014701	174690404	1	rs930734	202318316	8.574E-28
608	1	rs74365829	174690558	1	rs930734	202318316	8.574E-28
609	1	rs60885521	174591547	1	rs10159280	202394287	8.814E-28
610	1	rs80324733	174776328	1	rs2292489	202536848	8.867E-28
611	1	rs76828045	174847442	1	rs10465591	202308344	9.345E-28
612	1	rs147728966	174848270	1	rs10465591	202308344	9.345E-28
613	1	rs60723087	174655617	1	rs2361453	202336314	9.477E-28
614	1	rs75062924	174664341	1	rs2361453	202336314	9.477E-28
615	1	rs74739388	174666752	1	rs2361453	202336314	9.477E-28
616	1	rs74225917	174686241	1	rs2361453	202336314	9.477E-28
617	1	rs59354288	174687003	1	rs2361453	202336314	9.477E-28
618	1	rs60665754	174687287	1	rs2361453	202336314	9.477E-28
619	1	rs76014701	174690404	1	rs2361453	202336314	9.477E-28

620	1	rs74365829	174690558	1	rs2361453	202336314	9.477E-28
621	1	rs78199276	174706357	1	rs930734	202318316	1.011E-27
622	1	rs6695956	174711947	1	rs930734	202318316	1.011E-27
623	1	rs77727672	174718575	1	rs930734	202318316	1.011E-27
624	1	rs79388999	174724332	1	rs930734	202318316	1.011E-27
625	1	rs61329817	174724485	1	rs930734	202318316	1.011E-27
626	1	rs60723087	174655617	1	rs6427950	202472066	1.029E-27
627	1	rs60723087	174655617	1	rs3753908	202511295	1.029E-27
628	1	rs60723087	174655617	1	rs2249811	202531741	1.029E-27
629	1	rs60723087	174655617	1	rs925532	202533880	1.029E-27
630	1	rs60723087	174655617	1	rs925533	202534108	1.029E-27
631	1	rs60723087	174655617	1	rs3767397	202534389	1.029E-27
632	1	rs60723087	174655617	1	rs3767395	202536140	1.029E-27
633	1	rs75062924	174664341	1	rs6427950	202472066	1.029E-27
634	1	rs75062924	174664341	1	rs3753908	202511295	1.029E-27
635	1	rs75062924	174664341	1	rs2249811	202531741	1.029E-27
636	1	rs75062924	174664341	1	rs925532	202533880	1.029E-27
637	1	rs75062924	174664341	1	rs925533	202534108	1.029E-27
638	1	rs75062924	174664341	1	rs3767397	202534389	1.029E-27
639	1	rs75062924	174664341	1	rs3767395	202536140	1.029E-27
640	1	rs74739388	174666752	1	rs6427950	202472066	1.029E-27
641	1	rs74739388	174666752	1	rs3753908	202511295	1.029E-27
642	1	rs74739388	174666752	1	rs2249811	202531741	1.029E-27
643	1	rs74739388	174666752	1	rs925532	202533880	1.029E-27
644	1	rs74739388	174666752	1	rs925533	202534108	1.029E-27
645	1	rs74739388	174666752	1	rs3767397	202534389	1.029E-27
646	1	rs74739388	174666752	1	rs3767395	202536140	1.029E-27
647	1	rs74225917	174686241	1	rs6427950	202472066	1.029E-27
648	1	rs74225917	174686241	1	rs3753908	202511295	1.029E-27
649	1	rs74225917	174686241	1	rs2249811	202531741	1.029E-27
650	1	rs74225917	174686241	1	rs925532	202533880	1.029E-27
651	1	rs74225917	174686241	1	rs925533	202534108	1.029E-27
652	1	rs74225917	174686241	1	rs3767397	202534389	1.029E-27
653	1	rs74225917	174686241	1	rs3767395	202536140	1.029E-27
654	1	rs59354288	174687003	1	rs6427950	202472066	1.029E-27
655	1	rs59354288	174687003	1	rs3753908	202511295	1.029E-27
656	1	rs59354288	174687003	1	rs2249811	202531741	1.029E-27
657	1	rs59354288	174687003	1	rs925532	202533880	1.029E-27
658	1	rs59354288	174687003	1	rs925533	202534108	1.029E-27
659	1	rs59354288	174687003	1	rs3767397	202534389	1.029E-27
660	1	rs59354288	174687003	1	rs3767395	202536140	1.029E-27
661	1	rs60665754	174687287	1	rs6427950	202472066	1.029E-27
662	1	rs60665754	174687287	1	rs3753908	202511295	1.029E-27
663	1	rs60665754	174687287	1	rs2249811	202531741	1.029E-27
664	1	rs60665754	174687287	1	rs925532	202533880	1.029E-27
665	1	rs60665754	174687287	1	rs925533	202534108	1.029E-27
666	1	rs60665754	174687287	1	rs3767397	202534389	1.029E-27
667	1	rs60665754	174687287	1	rs3767395	202536140	1.029E-27
668	1	rs76014701	174690404	1	rs6427950	202472066	1.029E-27
669	1	rs76014701	174690404	1	rs3753908	202511295	1.029E-27
670	1	rs76014701	174690404	1	rs2249811	202531741	1.029E-27
671	1	rs76014701	174690404	1	rs925532	202533880	1.029E-27

672	1	rs76014701	174690404	1	rs925533	202534108	1.029E-27
673	1	rs76014701	174690404	1	rs3767397	202534389	1.029E-27
674	1	rs76014701	174690404	1	rs3767395	202536140	1.029E-27
675	1	rs74365829	174690558	1	rs6427950	202472066	1.029E-27
676	1	rs74365829	174690558	1	rs3753908	202511295	1.029E-27
677	1	rs74365829	174690558	1	rs2249811	202531741	1.029E-27
678	1	rs74365829	174690558	1	rs925532	202533880	1.029E-27
679	1	rs74365829	174690558	1	rs925533	202534108	1.029E-27
680	1	rs74365829	174690558	1	rs3767397	202534389	1.029E-27
681	1	rs74365829	174690558	1	rs3767395	202536140	1.029E-27
682	1	rs56864802	174568929	1	rs2292489	202536848	1.072E-27
683	1	rs60240702	174569231	1	rs2292489	202536848	1.072E-27
684	1	rs77531515	174572000	1	rs2292489	202536848	1.072E-27
685	1	rs77025642	174572929	1	rs2292489	202536848	1.072E-27
686	1	rs79989938	174575187	1	rs2292489	202536848	1.072E-27
687	1	rs114289262	174587137	1	rs2292489	202536848	1.073E-27
688	1	rs78199276	174706357	1	rs2361453	202336314	1.117E-27
689	1	rs6695956	174711947	1	rs2361453	202336314	1.117E-27
690	1	rs77727672	174718575	1	rs2361453	202336314	1.117E-27
691	1	rs79388999	174724332	1	rs2361453	202336314	1.117E-27
692	1	rs61329817	174724485	1	rs2361453	202336314	1.117E-27
693	1	rs79763699	174729671	1	rs2292489	202536848	1.137E-27
694	1	rs139161617	174732199	1	rs2292489	202536848	1.137E-27
695	1	rs6673649	174733562	1	rs2292489	202536848	1.137E-27
696	1	rs75912125	174740527	1	rs2292489	202536848	1.137E-27
697	1	rs76616345	174766778	1	rs930734	202318316	1.184E-27
698	1	rs78199276	174706357	1	rs6427950	202472066	1.212E-27
699	1	rs78199276	174706357	1	rs3753908	202511295	1.212E-27
700	1	rs78199276	174706357	1	rs2249811	202531741	1.212E-27
701	1	rs78199276	174706357	1	rs925532	202533880	1.212E-27
702	1	rs78199276	174706357	1	rs925533	202534108	1.212E-27
703	1	rs78199276	174706357	1	rs3767397	202534389	1.212E-27
704	1	rs78199276	174706357	1	rs3767395	202536140	1.212E-27
705	1	rs6695956	174711947	1	rs6427950	202472066	1.212E-27
706	1	rs6695956	174711947	1	rs3753908	202511295	1.212E-27
707	1	rs6695956	174711947	1	rs2249811	202531741	1.212E-27
708	1	rs6695956	174711947	1	rs925532	202533880	1.212E-27
709	1	rs6695956	174711947	1	rs925533	202534108	1.212E-27
710	1	rs6695956	174711947	1	rs3767397	202534389	1.212E-27
711	1	rs6695956	174711947	1	rs3767395	202536140	1.212E-27
712	1	rs77727672	174718575	1	rs6427950	202472066	1.212E-27
713	1	rs77727672	174718575	1	rs3753908	202511295	1.212E-27
714	1	rs77727672	174718575	1	rs2249811	202531741	1.212E-27
715	1	rs77727672	174718575	1	rs925532	202533880	1.212E-27
716	1	rs77727672	174718575	1	rs925533	202534108	1.212E-27
717	1	rs77727672	174718575	1	rs3767397	202534389	1.212E-27
718	1	rs77727672	174718575	1	rs3767395	202536140	1.212E-27
719	1	rs79388999	174724332	1	rs6427950	202472066	1.212E-27
720	1	rs79388999	174724332	1	rs3753908	202511295	1.212E-27
721	1	rs79388999	174724332	1	rs2249811	202531741	1.212E-27
722	1	rs79388999	174724332	1	rs925532	202533880	1.212E-27
723	1	rs79388999	174724332	1	rs925533	202534108	1.212E-27

724	1	rs79388999	174724332	1	rs3767397	202534389	1.212E-27
725	1	rs79388999	174724332	1	rs3767395	202536140	1.212E-27
726	1	rs61329817	174724485	1	rs6427950	202472066	1.212E-27
727	1	rs61329817	174724485	1	rs3753908	202511295	1.212E-27
728	1	rs61329817	174724485	1	rs2249811	202531741	1.212E-27
729	1	rs61329817	174724485	1	rs925532	202533880	1.212E-27
730	1	rs61329817	174724485	1	rs925533	202534108	1.212E-27
731	1	rs61329817	174724485	1	rs3767397	202534389	1.212E-27
732	1	rs61329817	174724485	1	rs3767395	202536140	1.212E-27
733	1	rs7543502	174653701	1	rs930734	202318316	1.257E-27
734	1	rs60723087	174655617	1	rs1890875	202531366	1.268E-27
735	1	rs75062924	174664341	1	rs1890875	202531366	1.268E-27
736	1	rs74739388	174666752	1	rs1890875	202531366	1.268E-27
737	1	rs74225917	174686241	1	rs1890875	202531366	1.268E-27
738	1	rs59354288	174687003	1	rs1890875	202531366	1.268E-27
739	1	rs60665754	174687287	1	rs1890875	202531366	1.268E-27
740	1	rs76014701	174690404	1	rs1890875	202531366	1.268E-27
741	1	rs74365829	174690558	1	rs1890875	202531366	1.268E-27
742	1	rs76616345	174766778	1	rs2361453	202336314	1.287E-27
743	1	rs60723087	174655617	1	rs10800836	202410429	1.33E-27
744	1	rs60723087	174655617	1	rs3767425	202413840	1.33E-27
745	1	rs60723087	174655617	1	rs3767421	202432160	1.33E-27
746	1	rs75062924	174664341	1	rs10800836	202410429	1.33E-27
747	1	rs75062924	174664341	1	rs3767425	202413840	1.33E-27
748	1	rs75062924	174664341	1	rs3767421	202432160	1.33E-27
749	1	rs74739388	174666752	1	rs10800836	202410429	1.33E-27
750	1	rs74739388	174666752	1	rs3767425	202413840	1.33E-27
751	1	rs74739388	174666752	1	rs3767421	202432160	1.33E-27
752	1	rs74225917	174686241	1	rs10800836	202410429	1.33E-27
753	1	rs74225917	174686241	1	rs3767425	202413840	1.33E-27
754	1	rs74225917	174686241	1	rs3767421	202432160	1.33E-27
755	1	rs59354288	174687003	1	rs10800836	202410429	1.33E-27
756	1	rs59354288	174687003	1	rs3767425	202413840	1.33E-27
757	1	rs59354288	174687003	1	rs3767421	202432160	1.33E-27
758	1	rs60665754	174687287	1	rs10800836	202410429	1.33E-27
759	1	rs60665754	174687287	1	rs3767425	202413840	1.33E-27
760	1	rs60665754	174687287	1	rs3767421	202432160	1.33E-27
761	1	rs76014701	174690404	1	rs10800836	202410429	1.33E-27
762	1	rs76014701	174690404	1	rs3767425	202413840	1.33E-27
763	1	rs76014701	174690404	1	rs3767421	202432160	1.33E-27
764	1	rs74365829	174690558	1	rs10800836	202410429	1.33E-27
765	1	rs74365829	174690558	1	rs3767425	202413840	1.33E-27
766	1	rs74365829	174690558	1	rs3767421	202432160	1.33E-27
767	1	rs138190123	174555301	1	rs2292489	202536848	1.341E-27
768	1	rs80324733	174776328	1	rs930734	202318316	1.381E-27
769	1	rs7543502	174653701	1	rs2361453	202336314	1.388E-27
770	1	rs60723087	174655617	1	rs10920392	202321123	1.42E-27
771	1	rs60723087	174655617	1	rs6658708	202322113	1.42E-27
772	1	rs75062924	174664341	1	rs10920392	202321123	1.42E-27
773	1	rs75062924	174664341	1	rs6658708	202322113	1.42E-27
774	1	rs74739388	174666752	1	rs10920392	202321123	1.42E-27
775	1	rs74739388	174666752	1	rs6658708	202322113	1.42E-27

776	1	rs74225917	174686241	1	rs10920392	202321123	1.42E-27
777	1	rs74225917	174686241	1	rs6658708	202322113	1.42E-27
778	1	rs59354288	174687003	1	rs10920392	202321123	1.42E-27
779	1	rs59354288	174687003	1	rs6658708	202322113	1.42E-27
780	1	rs60665754	174687287	1	rs10920392	202321123	1.42E-27
781	1	rs60665754	174687287	1	rs6658708	202322113	1.42E-27
782	1	rs76014701	174690404	1	rs10920392	202321123	1.42E-27
783	1	rs76014701	174690404	1	rs6658708	202322113	1.42E-27
784	1	rs74365829	174690558	1	rs10920392	202321123	1.42E-27
785	1	rs74365829	174690558	1	rs6658708	202322113	1.42E-27
786	1	rs76616345	174766778	1	rs6427950	202472066	1.448E-27
787	1	rs76616345	174766778	1	rs3753908	202511295	1.448E-27
788	1	rs76616345	174766778	1	rs2249811	202531741	1.448E-27
789	1	rs76616345	174766778	1	rs925532	202533880	1.448E-27
790	1	rs76616345	174766778	1	rs925533	202534108	1.448E-27
791	1	rs76616345	174766778	1	rs3767397	202534389	1.448E-27
792	1	rs76616345	174766778	1	rs3767395	202536140	1.448E-27
793	1	rs78199276	174706357	1	rs1890875	202531366	1.491E-27
794	1	rs6695956	174711947	1	rs1890875	202531366	1.491E-27
795	1	rs77727672	174718575	1	rs1890875	202531366	1.491E-27
796	1	rs79388999	174724332	1	rs1890875	202531366	1.491E-27
797	1	rs61329817	174724485	1	rs1890875	202531366	1.491E-27
798	1	rs7543502	174653701	1	rs6427950	202472066	1.511E-27
799	1	rs7543502	174653701	1	rs3753908	202511295	1.511E-27
800	1	rs7543502	174653701	1	rs2249811	202531741	1.511E-27
801	1	rs7543502	174653701	1	rs925532	202533880	1.511E-27
802	1	rs7543502	174653701	1	rs925533	202534108	1.511E-27
803	1	rs7543502	174653701	1	rs3767397	202534389	1.511E-27
804	1	rs7543502	174653701	1	rs3767395	202536140	1.511E-27
805	1	rs80324733	174776328	1	rs2361453	202336314	1.516E-27
806	1	rs78199276	174706357	1	rs10800836	202410429	1.564E-27
807	1	rs78199276	174706357	1	rs3767425	202413840	1.564E-27
808	1	rs78199276	174706357	1	rs3767421	202432160	1.564E-27
809	1	rs6695956	174711947	1	rs10800836	202410429	1.564E-27
810	1	rs6695956	174711947	1	rs3767425	202413840	1.564E-27
811	1	rs6695956	174711947	1	rs3767421	202432160	1.564E-27
812	1	rs77727672	174718575	1	rs10800836	202410429	1.564E-27
813	1	rs77727672	174718575	1	rs3767425	202413840	1.564E-27
814	1	rs77727672	174718575	1	rs3767421	202432160	1.564E-27
815	1	rs79388999	174724332	1	rs10800836	202410429	1.564E-27
816	1	rs79388999	174724332	1	rs3767425	202413840	1.564E-27
817	1	rs79388999	174724332	1	rs3767421	202432160	1.564E-27
818	1	rs61329817	174724485	1	rs10800836	202410429	1.564E-27
819	1	rs61329817	174724485	1	rs3767425	202413840	1.564E-27
820	1	rs61329817	174724485	1	rs3767421	202432160	1.564E-27
821	1	rs80324733	174776328	1	rs6427950	202472066	1.649E-27
822	1	rs80324733	174776328	1	rs3753908	202511295	1.649E-27
823	1	rs80324733	174776328	1	rs2249811	202531741	1.649E-27
824	1	rs80324733	174776328	1	rs925532	202533880	1.649E-27
825	1	rs80324733	174776328	1	rs925533	202534108	1.649E-27
826	1	rs80324733	174776328	1	rs3767397	202534389	1.649E-27
827	1	rs80324733	174776328	1	rs3767395	202536140	1.649E-27

828	1	rs78199276	174706357	1	rs10920392	202321123	1.671E-27
829	1	rs78199276	174706357	1	rs6658708	202322113	1.671E-27
830	1	rs6695956	174711947	1	rs10920392	202321123	1.671E-27
831	1	rs6695956	174711947	1	rs6658708	202322113	1.671E-27
832	1	rs77727672	174718575	1	rs10920392	202321123	1.671E-27
833	1	rs77727672	174718575	1	rs6658708	202322113	1.671E-27
834	1	rs79388999	174724332	1	rs10920392	202321123	1.671E-27
835	1	rs79388999	174724332	1	rs6658708	202322113	1.671E-27
836	1	rs61329817	174724485	1	rs10920392	202321123	1.671E-27
837	1	rs61329817	174724485	1	rs6658708	202322113	1.671E-27
838	1	rs59190079	174594608	1	rs2292489	202536848	1.702E-27
839	1	rs56864802	174568929	1	rs930734	202318316	1.737E-27
840	1	rs60240702	174569231	1	rs930734	202318316	1.737E-27
841	1	rs77531515	174572000	1	rs930734	202318316	1.737E-27
842	1	rs77025642	174572929	1	rs930734	202318316	1.737E-27
843	1	rs79989938	174575187	1	rs930734	202318316	1.737E-27
844	1	rs114289262	174587137	1	rs930734	202318316	1.741E-27
845	1	rs60723087	174655617	1	rs10159280	202394287	1.749E-27
846	1	rs75062924	174664341	1	rs10159280	202394287	1.749E-27
847	1	rs74739388	174666752	1	rs10159280	202394287	1.749E-27
848	1	rs74225917	174686241	1	rs10159280	202394287	1.749E-27
849	1	rs59354288	174687003	1	rs10159280	202394287	1.749E-27
850	1	rs60665754	174687287	1	rs10159280	202394287	1.749E-27
851	1	rs76014701	174690404	1	rs10159280	202394287	1.749E-27
852	1	rs74365829	174690558	1	rs10159280	202394287	1.749E-27
853	1	rs60723087	174655617	1	rs705742	202461982	1.771E-27
854	1	rs75062924	174664341	1	rs705742	202461982	1.771E-27
855	1	rs74739388	174666752	1	rs705742	202461982	1.771E-27
856	1	rs74225917	174686241	1	rs705742	202461982	1.771E-27
857	1	rs59354288	174687003	1	rs705742	202461982	1.771E-27
858	1	rs60665754	174687287	1	rs705742	202461982	1.771E-27
859	1	rs76014701	174690404	1	rs705742	202461982	1.771E-27
860	1	rs74365829	174690558	1	rs705742	202461982	1.771E-27
861	1	rs76616345	174766778	1	rs1890875	202531366	1.777E-27
862	1	rs16847018	174419672	1	rs2292489	202536848	1.821E-27
863	1	rs16847025	174425858	1	rs2292489	202536848	1.821E-27
864	1	rs75368101	174447029	1	rs2292489	202536848	1.821E-27
865	1	rs74367921	174457505	1	rs2292489	202536848	1.821E-27
866	1	rs141587031	174458302	1	rs2292489	202536848	1.821E-27
867	1	rs75222047	174460751	1	rs2292489	202536848	1.821E-27
868	1	rs77684114	174461386	1	rs2292489	202536848	1.821E-27
869	1	rs79170014	174462952	1	rs2292489	202536848	1.821E-27
870	1	rs2901819	174467697	1	rs2292489	202536848	1.821E-27
871	1	rs58063264	174468442	1	rs2292489	202536848	1.821E-27
872	1	rs41397546	174476714	1	rs2292489	202536848	1.821E-27
873	1	rs16847121	174482433	1	rs2292489	202536848	1.821E-27
874	1	rs76886469	174488738	1	rs2292489	202536848	1.821E-27
875	1	rs57132183	174492721	1	rs2292489	202536848	1.821E-27
876	1	rs74651209	174501445	1	rs2292489	202536848	1.821E-27
877	1	rs59262554	174504233	1	rs2292489	202536848	1.821E-27
878	1	rs7547843	174507529	1	rs2292489	202536848	1.821E-27
879	1	rs142531097	174512268	1	rs2292489	202536848	1.821E-27

880	1	rs146786952	174514535	1	rs2292489	202536848	1.821E-27
881	1	rs41266052	174517443	1	rs2292489	202536848	1.821E-27
882	1	rs57898822	174531784	1	rs2292489	202536848	1.821E-27
883	1	rs75120198	174535022	1	rs2292489	202536848	1.821E-27
884	1	rs75134210	174536483	1	rs2292489	202536848	1.821E-27
885	1	rs2179109	174540259	1	rs2292489	202536848	1.821E-27
886	1	rs16847206	174541307	1	rs2292489	202536848	1.821E-27
887	1	rs148694367	174544318	1	rs2292489	202536848	1.821E-27
888	1	rs143179310	174545451	1	rs2292489	202536848	1.821E-27
889	1	rs79763699	174729671	1	rs930734	202318316	1.828E-27
890	1	rs139161617	174732199	1	rs930734	202318316	1.828E-27
891	1	rs6673649	174733562	1	rs930734	202318316	1.828E-27
892	1	rs75912125	174740527	1	rs930734	202318316	1.828E-27
893	1	rs76616345	174766778	1	rs10800836	202410429	1.85E-27
894	1	rs76616345	174766778	1	rs3767425	202413840	1.85E-27
895	1	rs76616345	174766778	1	rs3767421	202432160	1.85E-27
896	1	rs7543502	174653701	1	rs1890875	202531366	1.863E-27
897	1	rs56864802	174568929	1	rs2361453	202336314	1.92E-27
898	1	rs60240702	174569231	1	rs2361453	202336314	1.92E-27
899	1	rs77531515	174572000	1	rs2361453	202336314	1.92E-27
900	1	rs77025642	174572929	1	rs2361453	202336314	1.92E-27
901	1	rs79989938	174575187	1	rs2361453	202336314	1.92E-27
902	1	rs114289262	174587137	1	rs2361453	202336314	1.928E-27
903	1	rs76616345	174766778	1	rs10920392	202321123	1.929E-27
904	1	rs76616345	174766778	1	rs6658708	202322113	1.929E-27
905	1	rs7543502	174653701	1	rs10800836	202410429	1.951E-27
906	1	rs7543502	174653701	1	rs3767425	202413840	1.951E-27
907	1	rs7543502	174653701	1	rs3767421	202432160	1.951E-27
908	1	rs80324733	174776328	1	rs1890875	202531366	2.006E-27
909	1	rs79763699	174729671	1	rs2361453	202336314	2.022E-27
910	1	rs139161617	174732199	1	rs2361453	202336314	2.022E-27
911	1	rs6673649	174733562	1	rs2361453	202336314	2.022E-27
912	1	rs75912125	174740527	1	rs2361453	202336314	2.022E-27
913	1	rs78199276	174706357	1	rs10159280	202394287	2.054E-27
914	1	rs6695956	174711947	1	rs10159280	202394287	2.054E-27
915	1	rs77727672	174718575	1	rs10159280	202394287	2.054E-27
916	1	rs79388999	174724332	1	rs10159280	202394287	2.054E-27
917	1	rs61329817	174724485	1	rs10159280	202394287	2.054E-27
918	1	rs56864802	174568929	1	rs6427950	202472066	2.075E-27
919	1	rs56864802	174568929	1	rs3753908	202511295	2.075E-27
920	1	rs56864802	174568929	1	rs2249811	202531741	2.075E-27
921	1	rs56864802	174568929	1	rs925532	202533880	2.075E-27
922	1	rs56864802	174568929	1	rs925533	202534108	2.075E-27
923	1	rs56864802	174568929	1	rs3767397	202534389	2.075E-27
924	1	rs56864802	174568929	1	rs3767395	202536140	2.075E-27
925	1	rs60240702	174569231	1	rs6427950	202472066	2.075E-27
926	1	rs60240702	174569231	1	rs3753908	202511295	2.075E-27
927	1	rs60240702	174569231	1	rs2249811	202531741	2.075E-27
928	1	rs60240702	174569231	1	rs925532	202533880	2.075E-27
929	1	rs60240702	174569231	1	rs925533	202534108	2.075E-27
930	1	rs60240702	174569231	1	rs3767397	202534389	2.075E-27
931	1	rs60240702	174569231	1	rs3767395	202536140	2.075E-27

932	1	rs77531515	174572000	1	rs6427950	202472066	2.075E-27
933	1	rs77531515	174572000	1	rs3753908	202511295	2.075E-27
934	1	rs77531515	174572000	1	rs2249811	202531741	2.075E-27
935	1	rs77531515	174572000	1	rs925532	202533880	2.075E-27
936	1	rs77531515	174572000	1	rs925533	202534108	2.075E-27
937	1	rs77531515	174572000	1	rs3767397	202534389	2.075E-27
938	1	rs77531515	174572000	1	rs3767395	202536140	2.075E-27
939	1	rs77025642	174572929	1	rs6427950	202472066	2.075E-27
940	1	rs77025642	174572929	1	rs3753908	202511295	2.075E-27
941	1	rs77025642	174572929	1	rs2249811	202531741	2.075E-27
942	1	rs77025642	174572929	1	rs925532	202533880	2.075E-27
943	1	rs77025642	174572929	1	rs925533	202534108	2.075E-27
944	1	rs77025642	174572929	1	rs3767397	202534389	2.075E-27
945	1	rs77025642	174572929	1	rs3767395	202536140	2.075E-27
946	1	rs79989938	174575187	1	rs6427950	202472066	2.075E-27
947	1	rs79989938	174575187	1	rs3753908	202511295	2.075E-27
948	1	rs79989938	174575187	1	rs2249811	202531741	2.075E-27
949	1	rs79989938	174575187	1	rs925532	202533880	2.075E-27
950	1	rs79989938	174575187	1	rs925533	202534108	2.075E-27
951	1	rs79989938	174575187	1	rs3767397	202534389	2.075E-27
952	1	rs79989938	174575187	1	rs3767395	202536140	2.075E-27
953	1	rs114289262	174587137	1	rs6427950	202472066	2.078E-27
954	1	rs114289262	174587137	1	rs3753908	202511295	2.078E-27
955	1	rs114289262	174587137	1	rs2249811	202531741	2.078E-27
956	1	rs114289262	174587137	1	rs925532	202533880	2.078E-27
957	1	rs114289262	174587137	1	rs925533	202534108	2.078E-27
958	1	rs114289262	174587137	1	rs3767397	202534389	2.078E-27
959	1	rs114289262	174587137	1	rs3767395	202536140	2.078E-27
960	1	rs7543502	174653701	1	rs10920392	202321123	2.08E-27
961	1	rs7543502	174653701	1	rs6658708	202322113	2.08E-27
962	1	rs80324733	174776328	1	rs10800836	202410429	2.091E-27
963	1	rs80324733	174776328	1	rs3767425	202413840	2.091E-27
964	1	rs80324733	174776328	1	rs3767421	202432160	2.091E-27
965	1	rs138190123	174555301	1	rs930734	202318316	2.176E-27
966	1	rs60885521	174591547	1	rs10465591	202308344	2.183E-27
967	1	rs79763699	174729671	1	rs6427950	202472066	2.196E-27
968	1	rs79763699	174729671	1	rs3753908	202511295	2.196E-27
969	1	rs79763699	174729671	1	rs2249811	202531741	2.196E-27
970	1	rs79763699	174729671	1	rs925532	202533880	2.196E-27
971	1	rs79763699	174729671	1	rs925533	202534108	2.196E-27
972	1	rs79763699	174729671	1	rs3767397	202534389	2.196E-27
973	1	rs79763699	174729671	1	rs3767395	202536140	2.196E-27
974	1	rs139161617	174732199	1	rs6427950	202472066	2.196E-27
975	1	rs139161617	174732199	1	rs3753908	202511295	2.196E-27
976	1	rs139161617	174732199	1	rs2249811	202531741	2.196E-27
977	1	rs139161617	174732199	1	rs925532	202533880	2.196E-27
978	1	rs139161617	174732199	1	rs925533	202534108	2.196E-27
979	1	rs139161617	174732199	1	rs3767397	202534389	2.196E-27
980	1	rs139161617	174732199	1	rs3767395	202536140	2.196E-27
981	1	rs6673649	174733562	1	rs6427950	202472066	2.196E-27
982	1	rs6673649	174733562	1	rs3753908	202511295	2.196E-27
983	1	rs6673649	174733562	1	rs2249811	202531741	2.196E-27

984	1	rs6673649	174733562	1	rs925532	202533880	2.196E-27
985	1	rs6673649	174733562	1	rs925533	202534108	2.196E-27
986	1	rs6673649	174733562	1	rs3767397	202534389	2.196E-27
987	1	rs6673649	174733562	1	rs3767395	202536140	2.196E-27
988	1	rs75912125	174740527	1	rs6427950	202472066	2.196E-27
989	1	rs75912125	174740527	1	rs3753908	202511295	2.196E-27
990	1	rs75912125	174740527	1	rs2249811	202531741	2.196E-27
991	1	rs75912125	174740527	1	rs925532	202533880	2.196E-27
992	1	rs75912125	174740527	1	rs925533	202534108	2.196E-27
993	1	rs75912125	174740527	1	rs3767397	202534389	2.196E-27
994	1	rs75912125	174740527	1	rs3767395	202536140	2.196E-27
995	1	rs80324733	174776328	1	rs10920392	202321123	2.216E-27
996	1	rs80324733	174776328	1	rs6658708	202322113	2.216E-27
997	1	rs78583223	174766014	1	rs2292489	202536848	2.241E-27
998	1	rs76616345	174766778	1	rs10159280	202394287	2.365E-27
999	1	rs138190123	174555301	1	rs2361453	202336314	2.412E-27
1000	1	rs56864802	174568929	1	rs1890875	202531366	2.556E-27
1001	1	rs60240702	174569231	1	rs1890875	202531366	2.556E-27
1002	1	rs77531515	174572000	1	rs1890875	202531366	2.556E-27
1003	1	rs77025642	174572929	1	rs1890875	202531366	2.556E-27
1004	1	rs79989938	174575187	1	rs1890875	202531366	2.556E-27
1005	1	rs114289262	174587137	1	rs1890875	202531366	2.561E-27
1006	1	rs7543502	174653701	1	rs10159280	202394287	2.562E-27
1007	1	rs138190123	174555301	1	rs6427950	202472066	2.597E-27
1008	1	rs138190123	174555301	1	rs3753908	202511295	2.597E-27
1009	1	rs138190123	174555301	1	rs2249811	202531741	2.597E-27
1010	1	rs138190123	174555301	1	rs925532	202533880	2.597E-27
1011	1	rs138190123	174555301	1	rs925533	202534108	2.597E-27
1012	1	rs138190123	174555301	1	rs3767397	202534389	2.597E-27
1013	1	rs138190123	174555301	1	rs3767395	202536140	2.597E-27
1014	1	rs74225911	174519824	1	rs2292489	202536848	2.63E-27
1015	1	rs56864802	174568929	1	rs10800836	202410429	2.679E-27
1016	1	rs56864802	174568929	1	rs3767425	202413840	2.679E-27
1017	1	rs56864802	174568929	1	rs3767421	202432160	2.679E-27
1018	1	rs60240702	174569231	1	rs10800836	202410429	2.679E-27
1019	1	rs60240702	174569231	1	rs3767425	202413840	2.679E-27
1020	1	rs60240702	174569231	1	rs3767421	202432160	2.679E-27
1021	1	rs77531515	174572000	1	rs10800836	202410429	2.679E-27
1022	1	rs77531515	174572000	1	rs3767425	202413840	2.679E-27
1023	1	rs77531515	174572000	1	rs3767421	202432160	2.679E-27
1024	1	rs77025642	174572929	1	rs10800836	202410429	2.679E-27
1025	1	rs77025642	174572929	1	rs3767425	202413840	2.679E-27
1026	1	rs77025642	174572929	1	rs3767421	202432160	2.679E-27
1027	1	rs79989938	174575187	1	rs10800836	202410429	2.679E-27
1028	1	rs79989938	174575187	1	rs3767425	202413840	2.679E-27
1029	1	rs79989938	174575187	1	rs3767421	202432160	2.679E-27
1030	1	rs114289262	174587137	1	rs10800836	202410429	2.684E-27
1031	1	rs114289262	174587137	1	rs3767425	202413840	2.684E-27
1032	1	rs114289262	174587137	1	rs3767421	202432160	2.684E-27
1033	1	rs80324733	174776328	1	rs10159280	202394287	2.695E-27
1034	1	rs79763699	174729671	1	rs1890875	202531366	2.698E-27
1035	1	rs139161617	174732199	1	rs1890875	202531366	2.698E-27

1036	1	rs6673649	174733562	1	rs1890875	202531366	2.698E-27
1037	1	rs75912125	174740527	1	rs1890875	202531366	2.698E-27
1038	1	rs59190079	174594608	1	rs930734	202318316	2.747E-27
1039	1	rs79763699	174729671	1	rs10800836	202410429	2.824E-27
1040	1	rs79763699	174729671	1	rs3767425	202413840	2.824E-27
1041	1	rs79763699	174729671	1	rs3767421	202432160	2.824E-27
1042	1	rs139161617	174732199	1	rs10800836	202410429	2.824E-27
1043	1	rs139161617	174732199	1	rs3767425	202413840	2.824E-27
1044	1	rs139161617	174732199	1	rs3767421	202432160	2.824E-27
1045	1	rs6673649	174733562	1	rs10800836	202410429	2.824E-27
1046	1	rs6673649	174733562	1	rs3767425	202413840	2.824E-27
1047	1	rs6673649	174733562	1	rs3767421	202432160	2.824E-27
1048	1	rs75912125	174740527	1	rs10800836	202410429	2.824E-27
1049	1	rs75912125	174740527	1	rs3767425	202413840	2.824E-27
1050	1	rs75912125	174740527	1	rs3767421	202432160	2.824E-27
1051	1	rs56864802	174568929	1	rs10920392	202321123	2.867E-27
1052	1	rs56864802	174568929	1	rs6658708	202322113	2.867E-27
1053	1	rs60240702	174569231	1	rs10920392	202321123	2.867E-27
1054	1	rs60240702	174569231	1	rs6658708	202322113	2.867E-27
1055	1	rs77531515	174572000	1	rs10920392	202321123	2.867E-27
1056	1	rs77531515	174572000	1	rs6658708	202322113	2.867E-27
1057	1	rs77025642	174572929	1	rs10920392	202321123	2.867E-27
1058	1	rs77025642	174572929	1	rs6658708	202322113	2.867E-27
1059	1	rs79989938	174575187	1	rs10920392	202321123	2.867E-27
1060	1	rs79989938	174575187	1	rs6658708	202322113	2.867E-27
1061	1	rs114289262	174587137	1	rs10920392	202321123	2.88E-27
1062	1	rs114289262	174587137	1	rs6658708	202322113	2.88E-27
1063	1	rs16847018	174419672	1	rs930734	202318316	2.944E-27
1064	1	rs16847025	174425858	1	rs930734	202318316	2.944E-27
1065	1	rs75368101	174447029	1	rs930734	202318316	2.944E-27
1066	1	rs74367921	174457505	1	rs930734	202318316	2.944E-27
1067	1	rs141587031	174458302	1	rs930734	202318316	2.944E-27
1068	1	rs75222047	174460751	1	rs930734	202318316	2.944E-27
1069	1	rs77684114	174461386	1	rs930734	202318316	2.944E-27
1070	1	rs79170014	174462952	1	rs930734	202318316	2.944E-27
1071	1	rs2901819	174467697	1	rs930734	202318316	2.944E-27
1072	1	rs58063264	174468442	1	rs930734	202318316	2.944E-27
1073	1	rs41397546	174476714	1	rs930734	202318316	2.944E-27
1074	1	rs16847121	174482433	1	rs930734	202318316	2.944E-27
1075	1	rs76886469	174488738	1	rs930734	202318316	2.944E-27
1076	1	rs57132183	174492721	1	rs930734	202318316	2.944E-27
1077	1	rs74651209	174501445	1	rs930734	202318316	2.944E-27
1078	1	rs59262554	174504233	1	rs930734	202318316	2.944E-27
1079	1	rs7547843	174507529	1	rs930734	202318316	2.944E-27
1080	1	rs142531097	174512268	1	rs930734	202318316	2.944E-27
1081	1	rs146786952	174514535	1	rs930734	202318316	2.944E-27
1082	1	rs41266052	174517443	1	rs930734	202318316	2.944E-27
1083	1	rs57898822	174531784	1	rs930734	202318316	2.944E-27
1084	1	rs75120198	174535022	1	rs930734	202318316	2.944E-27
1085	1	rs75134210	174536483	1	rs930734	202318316	2.944E-27
1086	1	rs2179109	174540259	1	rs930734	202318316	2.944E-27
1087	1	rs16847206	174541307	1	rs930734	202318316	2.944E-27

1088	1	rs148694367	174544318	1	rs930734	202318316	2.944E-27
1089	1	rs143179310	174545451	1	rs930734	202318316	2.944E-27
1090	1	rs79763699	174729671	1	rs10920392	202321123	3.014E-27
1091	1	rs79763699	174729671	1	rs6658708	202322113	3.014E-27
1092	1	rs139161617	174732199	1	rs10920392	202321123	3.014E-27
1093	1	rs139161617	174732199	1	rs6658708	202322113	3.014E-27
1094	1	rs6673649	174733562	1	rs10920392	202321123	3.014E-27
1095	1	rs6673649	174733562	1	rs6658708	202322113	3.014E-27
1096	1	rs75912125	174740527	1	rs10920392	202321123	3.014E-27
1097	1	rs75912125	174740527	1	rs6658708	202322113	3.014E-27
1098	1	rs59190079	174594608	1	rs2361453	202336314	3.037E-27
1099	1	rs138190123	174555301	1	rs1890875	202531366	3.202E-27
1100	1	rs16847018	174419672	1	rs2361453	202336314	3.258E-27
1101	1	rs16847025	174425858	1	rs2361453	202336314	3.258E-27
1102	1	rs75368101	174447029	1	rs2361453	202336314	3.258E-27
1103	1	rs74367921	174457505	1	rs2361453	202336314	3.258E-27
1104	1	rs141587031	174458302	1	rs2361453	202336314	3.258E-27
1105	1	rs75222047	174460751	1	rs2361453	202336314	3.258E-27
1106	1	rs77684114	174461386	1	rs2361453	202336314	3.258E-27
1107	1	rs79170014	174462952	1	rs2361453	202336314	3.258E-27
1108	1	rs2901819	174467697	1	rs2361453	202336314	3.258E-27
1109	1	rs58063264	174468442	1	rs2361453	202336314	3.258E-27
1110	1	rs41397546	174476714	1	rs2361453	202336314	3.258E-27
1111	1	rs16847121	174482433	1	rs2361453	202336314	3.258E-27
1112	1	rs76886469	174488738	1	rs2361453	202336314	3.258E-27
1113	1	rs57132183	174492721	1	rs2361453	202336314	3.258E-27
1114	1	rs74651209	174501445	1	rs2361453	202336314	3.258E-27
1115	1	rs59262554	174504233	1	rs2361453	202336314	3.258E-27
1116	1	rs7547843	174507529	1	rs2361453	202336314	3.258E-27
1117	1	rs142531097	174512268	1	rs2361453	202336314	3.258E-27
1118	1	rs146786952	174514535	1	rs2361453	202336314	3.258E-27
1119	1	rs41266052	174517443	1	rs2361453	202336314	3.258E-27
1120	1	rs57898822	174531784	1	rs2361453	202336314	3.258E-27
1121	1	rs75120198	174535022	1	rs2361453	202336314	3.258E-27
1122	1	rs75134210	174536483	1	rs2361453	202336314	3.258E-27
1123	1	rs2179109	174540259	1	rs2361453	202336314	3.258E-27
1124	1	rs16847206	174541307	1	rs2361453	202336314	3.258E-27
1125	1	rs148694367	174544318	1	rs2361453	202336314	3.258E-27
1126	1	rs143179310	174545451	1	rs2361453	202336314	3.258E-27
1127	1	rs59190079	174594608	1	rs6427950	202472066	3.286E-27
1128	1	rs59190079	174594608	1	rs3753908	202511295	3.286E-27
1129	1	rs59190079	174594608	1	rs2249811	202531741	3.286E-27
1130	1	rs59190079	174594608	1	rs925532	202533880	3.286E-27
1131	1	rs59190079	174594608	1	rs925533	202534108	3.286E-27
1132	1	rs59190079	174594608	1	rs3767397	202534389	3.286E-27
1133	1	rs59190079	174594608	1	rs3767395	202536140	3.286E-27
1134	1	rs138190123	174555301	1	rs10800836	202410429	3.353E-27
1135	1	rs138190123	174555301	1	rs3767425	202413840	3.353E-27
1136	1	rs138190123	174555301	1	rs3767421	202432160	3.353E-27
1137	1	rs16847018	174419672	1	rs6427950	202472066	3.52E-27
1138	1	rs16847018	174419672	1	rs3753908	202511295	3.52E-27
1139	1	rs16847018	174419672	1	rs2249811	202531741	3.52E-27

1140	1	rs16847018	174419672	1	rs925532	202533880	3.52E-27
1141	1	rs16847018	174419672	1	rs925533	202534108	3.52E-27
1142	1	rs16847018	174419672	1	rs3767397	202534389	3.52E-27
1143	1	rs16847018	174419672	1	rs3767395	202536140	3.52E-27
1144	1	rs16847025	174425858	1	rs6427950	202472066	3.52E-27
1145	1	rs16847025	174425858	1	rs3753908	202511295	3.52E-27
1146	1	rs16847025	174425858	1	rs2249811	202531741	3.52E-27
1147	1	rs16847025	174425858	1	rs925532	202533880	3.52E-27
1148	1	rs16847025	174425858	1	rs925533	202534108	3.52E-27
1149	1	rs16847025	174425858	1	rs3767397	202534389	3.52E-27
1150	1	rs16847025	174425858	1	rs3767395	202536140	3.52E-27
1151	1	rs75368101	174447029	1	rs6427950	202472066	3.52E-27
1152	1	rs75368101	174447029	1	rs3753908	202511295	3.52E-27
1153	1	rs75368101	174447029	1	rs2249811	202531741	3.52E-27
1154	1	rs75368101	174447029	1	rs925532	202533880	3.52E-27
1155	1	rs75368101	174447029	1	rs925533	202534108	3.52E-27
1156	1	rs75368101	174447029	1	rs3767397	202534389	3.52E-27
1157	1	rs75368101	174447029	1	rs3767395	202536140	3.52E-27
1158	1	rs74367921	174457505	1	rs6427950	202472066	3.52E-27
1159	1	rs74367921	174457505	1	rs3753908	202511295	3.52E-27
1160	1	rs74367921	174457505	1	rs2249811	202531741	3.52E-27
1161	1	rs74367921	174457505	1	rs925532	202533880	3.52E-27
1162	1	rs74367921	174457505	1	rs925533	202534108	3.52E-27
1163	1	rs74367921	174457505	1	rs3767397	202534389	3.52E-27
1164	1	rs74367921	174457505	1	rs3767395	202536140	3.52E-27
1165	1	rs141587031	174458302	1	rs6427950	202472066	3.52E-27
1166	1	rs141587031	174458302	1	rs3753908	202511295	3.52E-27
1167	1	rs141587031	174458302	1	rs2249811	202531741	3.52E-27
1168	1	rs141587031	174458302	1	rs925532	202533880	3.52E-27
1169	1	rs141587031	174458302	1	rs925533	202534108	3.52E-27
1170	1	rs141587031	174458302	1	rs3767397	202534389	3.52E-27
1171	1	rs141587031	174458302	1	rs3767395	202536140	3.52E-27
1172	1	rs75222047	174460751	1	rs6427950	202472066	3.52E-27
1173	1	rs75222047	174460751	1	rs3753908	202511295	3.52E-27
1174	1	rs75222047	174460751	1	rs2249811	202531741	3.52E-27
1175	1	rs75222047	174460751	1	rs925532	202533880	3.52E-27
1176	1	rs75222047	174460751	1	rs925533	202534108	3.52E-27
1177	1	rs75222047	174460751	1	rs3767397	202534389	3.52E-27
1178	1	rs75222047	174460751	1	rs3767395	202536140	3.52E-27
1179	1	rs77684114	174461386	1	rs6427950	202472066	3.52E-27
1180	1	rs77684114	174461386	1	rs3753908	202511295	3.52E-27
1181	1	rs77684114	174461386	1	rs2249811	202531741	3.52E-27
1182	1	rs77684114	174461386	1	rs925532	202533880	3.52E-27
1183	1	rs77684114	174461386	1	rs925533	202534108	3.52E-27
1184	1	rs77684114	174461386	1	rs3767397	202534389	3.52E-27
1185	1	rs77684114	174461386	1	rs3767395	202536140	3.52E-27
1186	1	rs79170014	174462952	1	rs6427950	202472066	3.52E-27
1187	1	rs79170014	174462952	1	rs3753908	202511295	3.52E-27
1188	1	rs79170014	174462952	1	rs2249811	202531741	3.52E-27
1189	1	rs79170014	174462952	1	rs925532	202533880	3.52E-27
1190	1	rs79170014	174462952	1	rs925533	202534108	3.52E-27
1191	1	rs79170014	174462952	1	rs3767397	202534389	3.52E-27

1192	1	rs79170014	174462952	1	rs3767395	202536140	3.52E-27
1193	1	rs2901819	174467697	1	rs6427950	202472066	3.52E-27
1194	1	rs2901819	174467697	1	rs3753908	202511295	3.52E-27
1195	1	rs2901819	174467697	1	rs2249811	202531741	3.52E-27
1196	1	rs2901819	174467697	1	rs925532	202533880	3.52E-27
1197	1	rs2901819	174467697	1	rs925533	202534108	3.52E-27
1198	1	rs2901819	174467697	1	rs3767397	202534389	3.52E-27
1199	1	rs2901819	174467697	1	rs3767395	202536140	3.52E-27
1200	1	rs58063264	174468442	1	rs6427950	202472066	3.52E-27
1201	1	rs58063264	174468442	1	rs3753908	202511295	3.52E-27
1202	1	rs58063264	174468442	1	rs2249811	202531741	3.52E-27
1203	1	rs58063264	174468442	1	rs925532	202533880	3.52E-27
1204	1	rs58063264	174468442	1	rs925533	202534108	3.52E-27
1205	1	rs58063264	174468442	1	rs3767397	202534389	3.52E-27
1206	1	rs58063264	174468442	1	rs3767395	202536140	3.52E-27
1207	1	rs41397546	174476714	1	rs6427950	202472066	3.52E-27
1208	1	rs41397546	174476714	1	rs3753908	202511295	3.52E-27
1209	1	rs41397546	174476714	1	rs2249811	202531741	3.52E-27
1210	1	rs41397546	174476714	1	rs925532	202533880	3.52E-27
1211	1	rs41397546	174476714	1	rs925533	202534108	3.52E-27
1212	1	rs41397546	174476714	1	rs3767397	202534389	3.52E-27
1213	1	rs41397546	174476714	1	rs3767395	202536140	3.52E-27
1214	1	rs16847121	174482433	1	rs6427950	202472066	3.52E-27
1215	1	rs16847121	174482433	1	rs3753908	202511295	3.52E-27
1216	1	rs16847121	174482433	1	rs2249811	202531741	3.52E-27
1217	1	rs16847121	174482433	1	rs925532	202533880	3.52E-27
1218	1	rs16847121	174482433	1	rs925533	202534108	3.52E-27
1219	1	rs16847121	174482433	1	rs3767397	202534389	3.52E-27
1220	1	rs16847121	174482433	1	rs3767395	202536140	3.52E-27
1221	1	rs76886469	174488738	1	rs6427950	202472066	3.52E-27
1222	1	rs76886469	174488738	1	rs3753908	202511295	3.52E-27
1223	1	rs76886469	174488738	1	rs2249811	202531741	3.52E-27
1224	1	rs76886469	174488738	1	rs925532	202533880	3.52E-27
1225	1	rs76886469	174488738	1	rs925533	202534108	3.52E-27
1226	1	rs76886469	174488738	1	rs3767397	202534389	3.52E-27
1227	1	rs76886469	174488738	1	rs3767395	202536140	3.52E-27
1228	1	rs57132183	174492721	1	rs6427950	202472066	3.52E-27
1229	1	rs57132183	174492721	1	rs3753908	202511295	3.52E-27
1230	1	rs57132183	174492721	1	rs2249811	202531741	3.52E-27
1231	1	rs57132183	174492721	1	rs925532	202533880	3.52E-27
1232	1	rs57132183	174492721	1	rs925533	202534108	3.52E-27
1233	1	rs57132183	174492721	1	rs3767397	202534389	3.52E-27
1234	1	rs57132183	174492721	1	rs3767395	202536140	3.52E-27
1235	1	rs74651209	174501445	1	rs6427950	202472066	3.52E-27
1236	1	rs74651209	174501445	1	rs3753908	202511295	3.52E-27
1237	1	rs74651209	174501445	1	rs2249811	202531741	3.52E-27
1238	1	rs74651209	174501445	1	rs925532	202533880	3.52E-27
1239	1	rs74651209	174501445	1	rs925533	202534108	3.52E-27
1240	1	rs74651209	174501445	1	rs3767397	202534389	3.52E-27
1241	1	rs74651209	174501445	1	rs3767395	202536140	3.52E-27
1242	1	rs59262554	174504233	1	rs6427950	202472066	3.52E-27
1243	1	rs59262554	174504233	1	rs3753908	202511295	3.52E-27

1244	1	rs59262554	174504233	1	rs2249811	202531741	3.52E-27
1245	1	rs59262554	174504233	1	rs925532	202533880	3.52E-27
1246	1	rs59262554	174504233	1	rs925533	202534108	3.52E-27
1247	1	rs59262554	174504233	1	rs3767397	202534389	3.52E-27
1248	1	rs59262554	174504233	1	rs3767395	202536140	3.52E-27
1249	1	rs7547843	174507529	1	rs6427950	202472066	3.52E-27
1250	1	rs7547843	174507529	1	rs3753908	202511295	3.52E-27
1251	1	rs7547843	174507529	1	rs2249811	202531741	3.52E-27
1252	1	rs7547843	174507529	1	rs925532	202533880	3.52E-27
1253	1	rs7547843	174507529	1	rs925533	202534108	3.52E-27
1254	1	rs7547843	174507529	1	rs3767397	202534389	3.52E-27
1255	1	rs7547843	174507529	1	rs3767395	202536140	3.52E-27
1256	1	rs142531097	174512268	1	rs6427950	202472066	3.52E-27
1257	1	rs142531097	174512268	1	rs3753908	202511295	3.52E-27
1258	1	rs142531097	174512268	1	rs2249811	202531741	3.52E-27
1259	1	rs142531097	174512268	1	rs925532	202533880	3.52E-27
1260	1	rs142531097	174512268	1	rs925533	202534108	3.52E-27
1261	1	rs142531097	174512268	1	rs3767397	202534389	3.52E-27
1262	1	rs142531097	174512268	1	rs3767395	202536140	3.52E-27
1263	1	rs146786952	174514535	1	rs6427950	202472066	3.52E-27
1264	1	rs146786952	174514535	1	rs3753908	202511295	3.52E-27
1265	1	rs146786952	174514535	1	rs2249811	202531741	3.52E-27
1266	1	rs146786952	174514535	1	rs925532	202533880	3.52E-27
1267	1	rs146786952	174514535	1	rs925533	202534108	3.52E-27
1268	1	rs146786952	174514535	1	rs3767397	202534389	3.52E-27
1269	1	rs146786952	174514535	1	rs3767395	202536140	3.52E-27
1270	1	rs41266052	174517443	1	rs6427950	202472066	3.52E-27
1271	1	rs41266052	174517443	1	rs3753908	202511295	3.52E-27
1272	1	rs41266052	174517443	1	rs2249811	202531741	3.52E-27
1273	1	rs41266052	174517443	1	rs925532	202533880	3.52E-27
1274	1	rs41266052	174517443	1	rs925533	202534108	3.52E-27
1275	1	rs41266052	174517443	1	rs3767397	202534389	3.52E-27
1276	1	rs41266052	174517443	1	rs3767395	202536140	3.52E-27
1277	1	rs57898822	174531784	1	rs6427950	202472066	3.52E-27
1278	1	rs57898822	174531784	1	rs3753908	202511295	3.52E-27
1279	1	rs57898822	174531784	1	rs2249811	202531741	3.52E-27
1280	1	rs57898822	174531784	1	rs925532	202533880	3.52E-27
1281	1	rs57898822	174531784	1	rs925533	202534108	3.52E-27
1282	1	rs57898822	174531784	1	rs3767397	202534389	3.52E-27
1283	1	rs57898822	174531784	1	rs3767395	202536140	3.52E-27
1284	1	rs75120198	174535022	1	rs6427950	202472066	3.52E-27
1285	1	rs75120198	174535022	1	rs3753908	202511295	3.52E-27
1286	1	rs75120198	174535022	1	rs2249811	202531741	3.52E-27
1287	1	rs75120198	174535022	1	rs925532	202533880	3.52E-27
1288	1	rs75120198	174535022	1	rs925533	202534108	3.52E-27
1289	1	rs75120198	174535022	1	rs3767397	202534389	3.52E-27
1290	1	rs75120198	174535022	1	rs3767395	202536140	3.52E-27
1291	1	rs75134210	174536483	1	rs6427950	202472066	3.52E-27
1292	1	rs75134210	174536483	1	rs3753908	202511295	3.52E-27
1293	1	rs75134210	174536483	1	rs2249811	202531741	3.52E-27
1294	1	rs75134210	174536483	1	rs925532	202533880	3.52E-27
1295	1	rs75134210	174536483	1	rs925533	202534108	3.52E-27

1296	1	rs75134210	174536483	1	rs3767397	202534389	3.52E-27
1297	1	rs75134210	174536483	1	rs3767395	202536140	3.52E-27
1298	1	rs2179109	174540259	1	rs6427950	202472066	3.52E-27
1299	1	rs2179109	174540259	1	rs3753908	202511295	3.52E-27
1300	1	rs2179109	174540259	1	rs2249811	202531741	3.52E-27
1301	1	rs2179109	174540259	1	rs925532	202533880	3.52E-27
1302	1	rs2179109	174540259	1	rs925533	202534108	3.52E-27
1303	1	rs2179109	174540259	1	rs3767397	202534389	3.52E-27
1304	1	rs2179109	174540259	1	rs3767395	202536140	3.52E-27
1305	1	rs16847206	174541307	1	rs6427950	202472066	3.52E-27
1306	1	rs16847206	174541307	1	rs3753908	202511295	3.52E-27
1307	1	rs16847206	174541307	1	rs2249811	202531741	3.52E-27
1308	1	rs16847206	174541307	1	rs925532	202533880	3.52E-27
1309	1	rs16847206	174541307	1	rs925533	202534108	3.52E-27
1310	1	rs16847206	174541307	1	rs3767397	202534389	3.52E-27
1311	1	rs16847206	174541307	1	rs3767395	202536140	3.52E-27
1312	1	rs148694367	174544318	1	rs6427950	202472066	3.52E-27
1313	1	rs148694367	174544318	1	rs3753908	202511295	3.52E-27
1314	1	rs148694367	174544318	1	rs2249811	202531741	3.52E-27
1315	1	rs148694367	174544318	1	rs925532	202533880	3.52E-27
1316	1	rs148694367	174544318	1	rs925533	202534108	3.52E-27
1317	1	rs148694367	174544318	1	rs3767397	202534389	3.52E-27
1318	1	rs148694367	174544318	1	rs3767395	202536140	3.52E-27
1319	1	rs143179310	174545451	1	rs6427950	202472066	3.52E-27
1320	1	rs143179310	174545451	1	rs3753908	202511295	3.52E-27
1321	1	rs143179310	174545451	1	rs2249811	202531741	3.52E-27
1322	1	rs143179310	174545451	1	rs925532	202533880	3.52E-27
1323	1	rs143179310	174545451	1	rs925533	202534108	3.52E-27
1324	1	rs143179310	174545451	1	rs3767397	202534389	3.52E-27
1325	1	rs143179310	174545451	1	rs3767395	202536140	3.52E-27
1326	1	rs56864802	174568929	1	rs10159280	202394287	3.53E-27
1327	1	rs60240702	174569231	1	rs10159280	202394287	3.53E-27
1328	1	rs77531515	174572000	1	rs10159280	202394287	3.53E-27
1329	1	rs77025642	174572929	1	rs10159280	202394287	3.53E-27
1330	1	rs79989938	174575187	1	rs10159280	202394287	3.53E-27
1331	1	rs114289262	174587137	1	rs10159280	202394287	3.546E-27
1332	1	rs138190123	174555301	1	rs10920392	202321123	3.605E-27
1333	1	rs138190123	174555301	1	rs6658708	202322113	3.605E-27
1334	1	rs78583223	174766014	1	rs930734	202318316	3.609E-27
1335	1	rs79763699	174729671	1	rs10159280	202394287	3.701E-27
1336	1	rs139161617	174732199	1	rs10159280	202394287	3.701E-27
1337	1	rs6673649	174733562	1	rs10159280	202394287	3.701E-27
1338	1	rs75912125	174740527	1	rs10159280	202394287	3.701E-27
1339	1	rs78583223	174766014	1	rs2361453	202336314	3.979E-27
1340	1	rs59190079	174594608	1	rs1890875	202531366	4.048E-27
1341	1	rs79387712	174605087	1	rs2292489	202536848	4.197E-27
1342	1	rs59190079	174594608	1	rs10800836	202410429	4.235E-27
1343	1	rs59190079	174594608	1	rs3767425	202413840	4.235E-27
1344	1	rs59190079	174594608	1	rs3767421	202432160	4.235E-27
1345	1	rs74225911	174519824	1	rs930734	202318316	4.241E-27
1346	1	rs78583223	174766014	1	rs6427950	202472066	4.312E-27
1347	1	rs78583223	174766014	1	rs3753908	202511295	4.312E-27

1348	1	rs78583223	174766014	1	rs2249811	202531741	4.312E-27
1349	1	rs78583223	174766014	1	rs925532	202533880	4.312E-27
1350	1	rs78583223	174766014	1	rs925533	202534108	4.312E-27
1351	1	rs78583223	174766014	1	rs3767397	202534389	4.312E-27
1352	1	rs78583223	174766014	1	rs3767395	202536140	4.312E-27
1353	1	rs16847018	174419672	1	rs1890875	202531366	4.333E-27
1354	1	rs16847025	174425858	1	rs1890875	202531366	4.333E-27
1355	1	rs75368101	174447029	1	rs1890875	202531366	4.333E-27
1356	1	rs74367921	174457505	1	rs1890875	202531366	4.333E-27
1357	1	rs141587031	174458302	1	rs1890875	202531366	4.333E-27
1358	1	rs75222047	174460751	1	rs1890875	202531366	4.333E-27
1359	1	rs77684114	174461386	1	rs1890875	202531366	4.333E-27
1360	1	rs79170014	174462952	1	rs1890875	202531366	4.333E-27
1361	1	rs2901819	174467697	1	rs1890875	202531366	4.333E-27
1362	1	rs58063264	174468442	1	rs1890875	202531366	4.333E-27
1363	1	rs41397546	174476714	1	rs1890875	202531366	4.333E-27
1364	1	rs16847121	174482433	1	rs1890875	202531366	4.333E-27
1365	1	rs76886469	174488738	1	rs1890875	202531366	4.333E-27
1366	1	rs57132183	174492721	1	rs1890875	202531366	4.333E-27
1367	1	rs74651209	174501445	1	rs1890875	202531366	4.333E-27
1368	1	rs59262554	174504233	1	rs1890875	202531366	4.333E-27
1369	1	rs7547843	174507529	1	rs1890875	202531366	4.333E-27
1370	1	rs142531097	174512268	1	rs1890875	202531366	4.333E-27
1371	1	rs146786952	174514535	1	rs1890875	202531366	4.333E-27
1372	1	rs41266052	174517443	1	rs1890875	202531366	4.333E-27
1373	1	rs57898822	174531784	1	rs1890875	202531366	4.333E-27
1374	1	rs75120198	174535022	1	rs1890875	202531366	4.333E-27
1375	1	rs75134210	174536483	1	rs1890875	202531366	4.333E-27
1376	1	rs2179109	174540259	1	rs1890875	202531366	4.333E-27
1377	1	rs16847206	174541307	1	rs1890875	202531366	4.333E-27
1378	1	rs148694367	174544318	1	rs1890875	202531366	4.333E-27
1379	1	rs143179310	174545451	1	rs1890875	202531366	4.333E-27
1380	1	rs60723087	174655617	1	rs10465591	202308344	4.341E-27
1381	1	rs75062924	174664341	1	rs10465591	202308344	4.341E-27
1382	1	rs74739388	174666752	1	rs10465591	202308344	4.341E-27
1383	1	rs74225917	174686241	1	rs10465591	202308344	4.341E-27
1384	1	rs59354288	174687003	1	rs10465591	202308344	4.341E-27
1385	1	rs60665754	174687287	1	rs10465591	202308344	4.341E-27
1386	1	rs76014701	174690404	1	rs10465591	202308344	4.341E-27
1387	1	rs74365829	174690558	1	rs10465591	202308344	4.341E-27
1388	1	rs138190123	174555301	1	rs10159280	202394287	4.44E-27
1389	1	rs59190079	174594608	1	rs10920392	202321123	4.533E-27
1390	1	rs59190079	174594608	1	rs6658708	202322113	4.533E-27
1391	1	rs16847018	174419672	1	rs10800836	202410429	4.539E-27
1392	1	rs16847018	174419672	1	rs3767425	202413840	4.539E-27
1393	1	rs16847018	174419672	1	rs3767421	202432160	4.539E-27
1394	1	rs16847025	174425858	1	rs10800836	202410429	4.539E-27
1395	1	rs16847025	174425858	1	rs3767425	202413840	4.539E-27
1396	1	rs16847025	174425858	1	rs3767421	202432160	4.539E-27
1397	1	rs75368101	174447029	1	rs10800836	202410429	4.539E-27
1398	1	rs75368101	174447029	1	rs3767425	202413840	4.539E-27
1399	1	rs75368101	174447029	1	rs3767421	202432160	4.539E-27

1400	1	rs74367921	174457505	1	rs10800836	202410429	4.539E-27
1401	1	rs74367921	174457505	1	rs3767425	202413840	4.539E-27
1402	1	rs74367921	174457505	1	rs3767421	202432160	4.539E-27
1403	1	rs141587031	174458302	1	rs10800836	202410429	4.539E-27
1404	1	rs141587031	174458302	1	rs3767425	202413840	4.539E-27
1405	1	rs141587031	174458302	1	rs3767421	202432160	4.539E-27
1406	1	rs75222047	174460751	1	rs10800836	202410429	4.539E-27
1407	1	rs75222047	174460751	1	rs3767425	202413840	4.539E-27
1408	1	rs75222047	174460751	1	rs3767421	202432160	4.539E-27
1409	1	rs77684114	174461386	1	rs10800836	202410429	4.539E-27
1410	1	rs77684114	174461386	1	rs3767425	202413840	4.539E-27
1411	1	rs77684114	174461386	1	rs3767421	202432160	4.539E-27
1412	1	rs79170014	174462952	1	rs10800836	202410429	4.539E-27
1413	1	rs79170014	174462952	1	rs3767425	202413840	4.539E-27
1414	1	rs79170014	174462952	1	rs3767421	202432160	4.539E-27
1415	1	rs2901819	174467697	1	rs10800836	202410429	4.539E-27
1416	1	rs2901819	174467697	1	rs3767425	202413840	4.539E-27
1417	1	rs2901819	174467697	1	rs3767421	202432160	4.539E-27
1418	1	rs58063264	174468442	1	rs10800836	202410429	4.539E-27
1419	1	rs58063264	174468442	1	rs3767425	202413840	4.539E-27
1420	1	rs58063264	174468442	1	rs3767421	202432160	4.539E-27
1421	1	rs41397546	174476714	1	rs10800836	202410429	4.539E-27
1422	1	rs41397546	174476714	1	rs3767425	202413840	4.539E-27
1423	1	rs41397546	174476714	1	rs3767421	202432160	4.539E-27
1424	1	rs16847121	174482433	1	rs10800836	202410429	4.539E-27
1425	1	rs16847121	174482433	1	rs3767425	202413840	4.539E-27
1426	1	rs16847121	174482433	1	rs3767421	202432160	4.539E-27
1427	1	rs76886469	174488738	1	rs10800836	202410429	4.539E-27
1428	1	rs76886469	174488738	1	rs3767425	202413840	4.539E-27
1429	1	rs76886469	174488738	1	rs3767421	202432160	4.539E-27
1430	1	rs57132183	174492721	1	rs10800836	202410429	4.539E-27
1431	1	rs57132183	174492721	1	rs3767425	202413840	4.539E-27
1432	1	rs57132183	174492721	1	rs3767421	202432160	4.539E-27
1433	1	rs74651209	174501445	1	rs10800836	202410429	4.539E-27
1434	1	rs74651209	174501445	1	rs3767425	202413840	4.539E-27
1435	1	rs74651209	174501445	1	rs3767421	202432160	4.539E-27
1436	1	rs59262554	174504233	1	rs10800836	202410429	4.539E-27
1437	1	rs59262554	174504233	1	rs3767425	202413840	4.539E-27
1438	1	rs59262554	174504233	1	rs3767421	202432160	4.539E-27
1439	1	rs7547843	174507529	1	rs10800836	202410429	4.539E-27
1440	1	rs7547843	174507529	1	rs3767425	202413840	4.539E-27
1441	1	rs7547843	174507529	1	rs3767421	202432160	4.539E-27
1442	1	rs142531097	174512268	1	rs10800836	202410429	4.539E-27
1443	1	rs142531097	174512268	1	rs3767425	202413840	4.539E-27
1444	1	rs142531097	174512268	1	rs3767421	202432160	4.539E-27
1445	1	rs146786952	174514535	1	rs10800836	202410429	4.539E-27
1446	1	rs146786952	174514535	1	rs3767425	202413840	4.539E-27
1447	1	rs146786952	174514535	1	rs3767421	202432160	4.539E-27
1448	1	rs41266052	174517443	1	rs10800836	202410429	4.539E-27
1449	1	rs41266052	174517443	1	rs3767425	202413840	4.539E-27
1450	1	rs41266052	174517443	1	rs3767421	202432160	4.539E-27
1451	1	rs57898822	174531784	1	rs10800836	202410429	4.539E-27

1452	1	rs57898822	174531784	1	rs3767425	202413840	4.539E-27
1453	1	rs57898822	174531784	1	rs3767421	202432160	4.539E-27
1454	1	rs75120198	174535022	1	rs10800836	202410429	4.539E-27
1455	1	rs75120198	174535022	1	rs3767425	202413840	4.539E-27
1456	1	rs75120198	174535022	1	rs3767421	202432160	4.539E-27
1457	1	rs75134210	174536483	1	rs10800836	202410429	4.539E-27
1458	1	rs75134210	174536483	1	rs3767425	202413840	4.539E-27
1459	1	rs75134210	174536483	1	rs3767421	202432160	4.539E-27
1460	1	rs2179109	174540259	1	rs10800836	202410429	4.539E-27
1461	1	rs2179109	174540259	1	rs3767425	202413840	4.539E-27
1462	1	rs2179109	174540259	1	rs3767421	202432160	4.539E-27
1463	1	rs16847206	174541307	1	rs10800836	202410429	4.539E-27
1464	1	rs16847206	174541307	1	rs3767425	202413840	4.539E-27
1465	1	rs16847206	174541307	1	rs3767421	202432160	4.539E-27
1466	1	rs148694367	174544318	1	rs10800836	202410429	4.539E-27
1467	1	rs148694367	174544318	1	rs3767425	202413840	4.539E-27
1468	1	rs148694367	174544318	1	rs3767421	202432160	4.539E-27
1469	1	rs143179310	174545451	1	rs10800836	202410429	4.539E-27
1470	1	rs143179310	174545451	1	rs3767425	202413840	4.539E-27
1471	1	rs143179310	174545451	1	rs3767421	202432160	4.539E-27
1472	1	rs74225911	174519824	1	rs2361453	202336314	4.684E-27
1473	1	rs76616345	174766778	1	rs10465591	202308344	4.701E-27
1474	1	rs16847018	174419672	1	rs10920392	202321123	4.859E-27
1475	1	rs16847018	174419672	1	rs6658708	202322113	4.859E-27
1476	1	rs16847025	174425858	1	rs10920392	202321123	4.859E-27
1477	1	rs16847025	174425858	1	rs6658708	202322113	4.859E-27
1478	1	rs75368101	174447029	1	rs10920392	202321123	4.859E-27
1479	1	rs75368101	174447029	1	rs6658708	202322113	4.859E-27
1480	1	rs74367921	174457505	1	rs10920392	202321123	4.859E-27
1481	1	rs74367921	174457505	1	rs6658708	202322113	4.859E-27
1482	1	rs141587031	174458302	1	rs10920392	202321123	4.859E-27
1483	1	rs141587031	174458302	1	rs6658708	202322113	4.859E-27
1484	1	rs75222047	174460751	1	rs10920392	202321123	4.859E-27
1485	1	rs75222047	174460751	1	rs6658708	202322113	4.859E-27
1486	1	rs77684114	174461386	1	rs10920392	202321123	4.859E-27
1487	1	rs77684114	174461386	1	rs6658708	202322113	4.859E-27
1488	1	rs79170014	174462952	1	rs10920392	202321123	4.859E-27
1489	1	rs79170014	174462952	1	rs6658708	202322113	4.859E-27
1490	1	rs2901819	174467697	1	rs10920392	202321123	4.859E-27
1491	1	rs2901819	174467697	1	rs6658708	202322113	4.859E-27
1492	1	rs58063264	174468442	1	rs10920392	202321123	4.859E-27
1493	1	rs58063264	174468442	1	rs6658708	202322113	4.859E-27
1494	1	rs41397546	174476714	1	rs10920392	202321123	4.859E-27
1495	1	rs41397546	174476714	1	rs6658708	202322113	4.859E-27
1496	1	rs16847121	174482433	1	rs10920392	202321123	4.859E-27
1497	1	rs16847121	174482433	1	rs6658708	202322113	4.859E-27
1498	1	rs76886469	174488738	1	rs10920392	202321123	4.859E-27
1499	1	rs76886469	174488738	1	rs6658708	202322113	4.859E-27
1500	1	rs57132183	174492721	1	rs10920392	202321123	4.859E-27
1501	1	rs57132183	174492721	1	rs6658708	202322113	4.859E-27
1502	1	rs74651209	174501445	1	rs10920392	202321123	4.859E-27
1503	1	rs74651209	174501445	1	rs6658708	202322113	4.859E-27

1504	1	rs59262554	174504233	1	rs10920392	202321123	4.859E-27
1505	1	rs59262554	174504233	1	rs6658708	202322113	4.859E-27
1506	1	rs7547843	174507529	1	rs10920392	202321123	4.859E-27
1507	1	rs7547843	174507529	1	rs6658708	202322113	4.859E-27
1508	1	rs142531097	174512268	1	rs10920392	202321123	4.859E-27
1509	1	rs142531097	174512268	1	rs6658708	202322113	4.859E-27
1510	1	rs146786952	174514535	1	rs10920392	202321123	4.859E-27
1511	1	rs146786952	174514535	1	rs6658708	202322113	4.859E-27
1512	1	rs41266052	174517443	1	rs10920392	202321123	4.859E-27
1513	1	rs41266052	174517443	1	rs6658708	202322113	4.859E-27
1514	1	rs57898822	174531784	1	rs10920392	202321123	4.859E-27
1515	1	rs57898822	174531784	1	rs6658708	202322113	4.859E-27
1516	1	rs75120198	174535022	1	rs10920392	202321123	4.859E-27
1517	1	rs75120198	174535022	1	rs6658708	202322113	4.859E-27
1518	1	rs75134210	174536483	1	rs10920392	202321123	4.859E-27
1519	1	rs75134210	174536483	1	rs6658708	202322113	4.859E-27
1520	1	rs2179109	174540259	1	rs10920392	202321123	4.859E-27
1521	1	rs2179109	174540259	1	rs6658708	202322113	4.859E-27
1522	1	rs16847206	174541307	1	rs10920392	202321123	4.859E-27
1523	1	rs16847206	174541307	1	rs6658708	202322113	4.859E-27
1524	1	rs148694367	174544318	1	rs10920392	202321123	4.859E-27
1525	1	rs148694367	174544318	1	rs6658708	202322113	4.859E-27
1526	1	rs143179310	174545451	1	rs10920392	202321123	4.859E-27
1527	1	rs143179310	174545451	1	rs6658708	202322113	4.859E-27
1528	1	rs79728530	174402618	1	rs2292489	202536848	5.035E-27
1529	1	rs78199276	174706357	1	rs10465591	202308344	5.075E-27
1530	1	rs6695956	174711947	1	rs10465591	202308344	5.075E-27
1531	1	rs77727672	174718575	1	rs10465591	202308344	5.075E-27
1532	1	rs79388999	174724332	1	rs10465591	202308344	5.075E-27
1533	1	rs61329817	174724485	1	rs10465591	202308344	5.075E-27
1534	1	rs74225911	174519824	1	rs6427950	202472066	5.076E-27
1535	1	rs74225911	174519824	1	rs3753908	202511295	5.076E-27
1536	1	rs74225911	174519824	1	rs2249811	202531741	5.076E-27
1537	1	rs74225911	174519824	1	rs925532	202533880	5.076E-27
1538	1	rs74225911	174519824	1	rs925533	202534108	5.076E-27
1539	1	rs74225911	174519824	1	rs3767397	202534389	5.076E-27
1540	1	rs74225911	174519824	1	rs3767395	202536140	5.076E-27
1541	1	rs76881580	174855306	1	rs705742	202461982	5.216E-27
1542	1	rs78583223	174766014	1	rs1890875	202531366	5.299E-27
1543	1	rs78583223	174766014	1	rs10800836	202410429	5.548E-27
1544	1	rs78583223	174766014	1	rs3767425	202413840	5.548E-27
1545	1	rs78583223	174766014	1	rs3767421	202432160	5.548E-27
1546	1	rs59190079	174594608	1	rs10159280	202394287	5.58E-27
1547	1	rs78583223	174766014	1	rs10920392	202321123	5.922E-27
1548	1	rs78583223	174766014	1	rs6658708	202322113	5.922E-27
1549	1	rs16847018	174419672	1	rs10159280	202394287	5.977E-27
1550	1	rs16847025	174425858	1	rs10159280	202394287	5.977E-27
1551	1	rs75368101	174447029	1	rs10159280	202394287	5.977E-27
1552	1	rs74367921	174457505	1	rs10159280	202394287	5.977E-27
1553	1	rs141587031	174458302	1	rs10159280	202394287	5.977E-27
1554	1	rs75222047	174460751	1	rs10159280	202394287	5.977E-27
1555	1	rs77684114	174461386	1	rs10159280	202394287	5.977E-27

1556	1	rs79170014	174462952	1	rs10159280	202394287	5.977E-27
1557	1	rs2901819	174467697	1	rs10159280	202394287	5.977E-27
1558	1	rs58063264	174468442	1	rs10159280	202394287	5.977E-27
1559	1	rs41397546	174476714	1	rs10159280	202394287	5.977E-27
1560	1	rs16847121	174482433	1	rs10159280	202394287	5.977E-27
1561	1	rs76886469	174488738	1	rs10159280	202394287	5.977E-27
1562	1	rs57132183	174492721	1	rs10159280	202394287	5.977E-27
1563	1	rs74651209	174501445	1	rs10159280	202394287	5.977E-27
1564	1	rs59262554	174504233	1	rs10159280	202394287	5.977E-27
1565	1	rs7547843	174507529	1	rs10159280	202394287	5.977E-27
1566	1	rs142531097	174512268	1	rs10159280	202394287	5.977E-27
1567	1	rs146786952	174514535	1	rs10159280	202394287	5.977E-27
1568	1	rs41266052	174517443	1	rs10159280	202394287	5.977E-27
1569	1	rs57898822	174531784	1	rs10159280	202394287	5.977E-27
1570	1	rs75120198	174535022	1	rs10159280	202394287	5.977E-27
1571	1	rs75134210	174536483	1	rs10159280	202394287	5.977E-27
1572	1	rs2179109	174540259	1	rs10159280	202394287	5.977E-27
1573	1	rs16847206	174541307	1	rs10159280	202394287	5.977E-27
1574	1	rs148694367	174544318	1	rs10159280	202394287	5.977E-27
1575	1	rs143179310	174545451	1	rs10159280	202394287	5.977E-27
1576	1	rs74225911	174519824	1	rs1890875	202531366	6.248E-27
1577	1	rs80324733	174776328	1	rs10465591	202308344	6.253E-27
1578	1	rs7543502	174653701	1	rs10465591	202308344	6.342E-27
1579	1	rs74225911	174519824	1	rs10800836	202410429	6.541E-27
1580	1	rs74225911	174519824	1	rs3767425	202413840	6.541E-27
1581	1	rs74225911	174519824	1	rs3767421	202432160	6.541E-27
1582	1	rs79387712	174605087	1	rs930734	202318316	6.785E-27
1583	1	rs74225911	174519824	1	rs10920392	202321123	6.992E-27
1584	1	rs74225911	174519824	1	rs6658708	202322113	6.992E-27
1585	1	rs78583223	174766014	1	rs10159280	202394287	7.273E-27
1586	1	rs79387712	174605087	1	rs2361453	202336314	7.485E-27
1587	1	rs79387712	174605087	1	rs6427950	202472066	8.071E-27
1588	1	rs79387712	174605087	1	rs3753908	202511295	8.071E-27
1589	1	rs79387712	174605087	1	rs2249811	202531741	8.071E-27
1590	1	rs79387712	174605087	1	rs925532	202533880	8.071E-27
1591	1	rs79387712	174605087	1	rs925533	202534108	8.071E-27
1592	1	rs79387712	174605087	1	rs3767397	202534389	8.071E-27
1593	1	rs79387712	174605087	1	rs3767395	202536140	8.071E-27
1594	1	rs79728530	174402618	1	rs930734	202318316	8.074E-27
1595	1	rs74761236	174619114	1	rs2292489	202536848	8.128E-27
1596	1	rs149356780	174619156	1	rs2292489	202536848	8.128E-27
1597	1	rs146122202	174619399	1	rs2292489	202536848	8.128E-27
1598	1	rs78172621	174624110	1	rs2292489	202536848	8.128E-27
1599	1	rs74641552	174635904	1	rs2292489	202536848	8.128E-27
1600	1	rs115697417	174639688	1	rs2292489	202536848	8.128E-27
1601	1	rs76311252	174640594	1	rs2292489	202536848	8.128E-27
1602	1	rs77714297	174641617	1	rs2292489	202536848	8.128E-27
1603	1	rs75540548	174648680	1	rs2292489	202536848	8.128E-27
1604	1	rs74225911	174519824	1	rs10159280	202394287	8.6E-27
1605	1	rs56864802	174568929	1	rs10465591	202308344	8.668E-27
1606	1	rs60240702	174569231	1	rs10465591	202308344	8.668E-27
1607	1	rs77531515	174572000	1	rs10465591	202308344	8.668E-27

1608	1	rs77025642	174572929	1	rs10465591	202308344	8.668E-27
1609	1	rs79989938	174575187	1	rs10465591	202308344	8.668E-27
1610	1	rs114289262	174587137	1	rs10465591	202308344	8.72E-27
1611	1	rs79728530	174402618	1	rs2361453	202336314	8.957E-27
1612	1	rs79763699	174729671	1	rs10465591	202308344	9.037E-27
1613	1	rs139161617	174732199	1	rs10465591	202308344	9.037E-27
1614	1	rs6673649	174733562	1	rs10465591	202308344	9.037E-27
1615	1	rs75912125	174740527	1	rs10465591	202308344	9.037E-27
1616	1	rs79728530	174402618	1	rs6427950	202472066	9.701E-27
1617	1	rs79728530	174402618	1	rs3753908	202511295	9.701E-27
1618	1	rs79728530	174402618	1	rs2249811	202531741	9.701E-27
1619	1	rs79728530	174402618	1	rs925532	202533880	9.701E-27
1620	1	rs79728530	174402618	1	rs925533	202534108	9.701E-27
1621	1	rs79728530	174402618	1	rs3767397	202534389	9.701E-27
1622	1	rs79728530	174402618	1	rs3767395	202536140	9.701E-27
1623	1	rs79387712	174605087	1	rs1890875	202531366	9.932E-27
1624	1	rs79387712	174605087	1	rs10800836	202410429	1.04E-26
1625	1	rs79387712	174605087	1	rs3767425	202413840	1.04E-26
1626	1	rs79387712	174605087	1	rs3767421	202432160	1.04E-26
1627	1	rs2179108	174540727	1	rs2292489	202536848	1.058E-26
1628	1	rs138190123	174555301	1	rs10465591	202308344	1.091E-26
1629	1	rs79387712	174605087	1	rs10920392	202321123	1.114E-26
1630	1	rs79387712	174605087	1	rs6658708	202322113	1.114E-26
1631	1	rs79728530	174402618	1	rs1890875	202531366	1.192E-26
1632	1	rs79728530	174402618	1	rs10800836	202410429	1.248E-26
1633	1	rs79728530	174402618	1	rs3767425	202413840	1.248E-26
1634	1	rs79728530	174402618	1	rs3767421	202432160	1.248E-26
1635	1	rs74761236	174619114	1	rs930734	202318316	1.315E-26
1636	1	rs149356780	174619156	1	rs930734	202318316	1.315E-26
1637	1	rs146122202	174619399	1	rs930734	202318316	1.315E-26
1638	1	rs78172621	174624110	1	rs930734	202318316	1.315E-26
1639	1	rs74641552	174635904	1	rs930734	202318316	1.315E-26
1640	1	rs115697417	174639688	1	rs930734	202318316	1.315E-26
1641	1	rs76311252	174640594	1	rs930734	202318316	1.315E-26
1642	1	rs77714297	174641617	1	rs930734	202318316	1.315E-26
1643	1	rs75540548	174648680	1	rs930734	202318316	1.315E-26
1644	1	rs79728530	174402618	1	rs10920392	202321123	1.334E-26
1645	1	rs79728530	174402618	1	rs6658708	202322113	1.334E-26
1646	1	rs6703536	174613295	1	rs2292489	202536848	1.339E-26
1647	1	rs59190079	174594608	1	rs10465591	202308344	1.364E-26
1648	1	rs79387712	174605087	1	rs10159280	202394287	1.37E-26
1649	1	rs74761236	174619114	1	rs2361453	202336314	1.449E-26
1650	1	rs149356780	174619156	1	rs2361453	202336314	1.449E-26
1651	1	rs146122202	174619399	1	rs2361453	202336314	1.449E-26
1652	1	rs78172621	174624110	1	rs2361453	202336314	1.449E-26
1653	1	rs74641552	174635904	1	rs2361453	202336314	1.449E-26
1654	1	rs115697417	174639688	1	rs2361453	202336314	1.449E-26
1655	1	rs76311252	174640594	1	rs2361453	202336314	1.449E-26
1656	1	rs77714297	174641617	1	rs2361453	202336314	1.449E-26
1657	1	rs75540548	174648680	1	rs2361453	202336314	1.449E-26
1658	1	rs16847018	174419672	1	rs10465591	202308344	1.464E-26
1659	1	rs16847025	174425858	1	rs10465591	202308344	1.464E-26

1660	1	rs75368101	174447029	1	rs10465591	202308344	1.464E-26
1661	1	rs74367921	174457505	1	rs10465591	202308344	1.464E-26
1662	1	rs141587031	174458302	1	rs10465591	202308344	1.464E-26
1663	1	rs75222047	174460751	1	rs10465591	202308344	1.464E-26
1664	1	rs77684114	174461386	1	rs10465591	202308344	1.464E-26
1665	1	rs79170014	174462952	1	rs10465591	202308344	1.464E-26
1666	1	rs2901819	174467697	1	rs10465591	202308344	1.464E-26
1667	1	rs58063264	174468442	1	rs10465591	202308344	1.464E-26
1668	1	rs41397546	174476714	1	rs10465591	202308344	1.464E-26
1669	1	rs16847121	174482433	1	rs10465591	202308344	1.464E-26
1670	1	rs76886469	174488738	1	rs10465591	202308344	1.464E-26
1671	1	rs57132183	174492721	1	rs10465591	202308344	1.464E-26
1672	1	rs74651209	174501445	1	rs10465591	202308344	1.464E-26
1673	1	rs59262554	174504233	1	rs10465591	202308344	1.464E-26
1674	1	rs7547843	174507529	1	rs10465591	202308344	1.464E-26
1675	1	rs142531097	174512268	1	rs10465591	202308344	1.464E-26
1676	1	rs146786952	174514535	1	rs10465591	202308344	1.464E-26
1677	1	rs41266052	174517443	1	rs10465591	202308344	1.464E-26
1678	1	rs57898822	174531784	1	rs10465591	202308344	1.464E-26
1679	1	rs75120198	174535022	1	rs10465591	202308344	1.464E-26
1680	1	rs75134210	174536483	1	rs10465591	202308344	1.464E-26
1681	1	rs2179109	174540259	1	rs10465591	202308344	1.464E-26
1682	1	rs16847206	174541307	1	rs10465591	202308344	1.464E-26
1683	1	rs148694367	174544318	1	rs10465591	202308344	1.464E-26
1684	1	rs143179310	174545451	1	rs10465591	202308344	1.464E-26
1685	1	rs74761236	174619114	1	rs6427950	202472066	1.567E-26
1686	1	rs74761236	174619114	1	rs3753908	202511295	1.567E-26
1687	1	rs74761236	174619114	1	rs2249811	202531741	1.567E-26
1688	1	rs74761236	174619114	1	rs925532	202533880	1.567E-26
1689	1	rs74761236	174619114	1	rs925533	202534108	1.567E-26
1690	1	rs74761236	174619114	1	rs3767397	202534389	1.567E-26
1691	1	rs74761236	174619114	1	rs3767395	202536140	1.567E-26
1692	1	rs149356780	174619156	1	rs6427950	202472066	1.567E-26
1693	1	rs149356780	174619156	1	rs3753908	202511295	1.567E-26
1694	1	rs149356780	174619156	1	rs2249811	202531741	1.567E-26
1695	1	rs149356780	174619156	1	rs925532	202533880	1.567E-26
1696	1	rs149356780	174619156	1	rs925533	202534108	1.567E-26
1697	1	rs149356780	174619156	1	rs3767397	202534389	1.567E-26
1698	1	rs149356780	174619156	1	rs3767395	202536140	1.567E-26
1699	1	rs146122202	174619399	1	rs6427950	202472066	1.567E-26
1700	1	rs146122202	174619399	1	rs3753908	202511295	1.567E-26
1701	1	rs146122202	174619399	1	rs2249811	202531741	1.567E-26
1702	1	rs146122202	174619399	1	rs925532	202533880	1.567E-26
1703	1	rs146122202	174619399	1	rs925533	202534108	1.567E-26
1704	1	rs146122202	174619399	1	rs3767397	202534389	1.567E-26
1705	1	rs146122202	174619399	1	rs3767395	202536140	1.567E-26
1706	1	rs78172621	174624110	1	rs6427950	202472066	1.567E-26
1707	1	rs78172621	174624110	1	rs3753908	202511295	1.567E-26
1708	1	rs78172621	174624110	1	rs2249811	202531741	1.567E-26
1709	1	rs78172621	174624110	1	rs925532	202533880	1.567E-26
1710	1	rs78172621	174624110	1	rs925533	202534108	1.567E-26
1711	1	rs78172621	174624110	1	rs3767397	202534389	1.567E-26

1712	1	rs78172621	174624110	1	rs3767395	202536140	1.567E-26
1713	1	rs74641552	174635904	1	rs6427950	202472066	1.567E-26
1714	1	rs74641552	174635904	1	rs3753908	202511295	1.567E-26
1715	1	rs74641552	174635904	1	rs2249811	202531741	1.567E-26
1716	1	rs74641552	174635904	1	rs925532	202533880	1.567E-26
1717	1	rs74641552	174635904	1	rs925533	202534108	1.567E-26
1718	1	rs74641552	174635904	1	rs3767397	202534389	1.567E-26
1719	1	rs74641552	174635904	1	rs3767395	202536140	1.567E-26
1720	1	rs115697417	174639688	1	rs6427950	202472066	1.567E-26
1721	1	rs115697417	174639688	1	rs3753908	202511295	1.567E-26
1722	1	rs115697417	174639688	1	rs2249811	202531741	1.567E-26
1723	1	rs115697417	174639688	1	rs925532	202533880	1.567E-26
1724	1	rs115697417	174639688	1	rs925533	202534108	1.567E-26
1725	1	rs115697417	174639688	1	rs3767397	202534389	1.567E-26
1726	1	rs115697417	174639688	1	rs3767395	202536140	1.567E-26
1727	1	rs76311252	174640594	1	rs6427950	202472066	1.567E-26
1728	1	rs76311252	174640594	1	rs3753908	202511295	1.567E-26
1729	1	rs76311252	174640594	1	rs2249811	202531741	1.567E-26
1730	1	rs76311252	174640594	1	rs925532	202533880	1.567E-26
1731	1	rs76311252	174640594	1	rs925533	202534108	1.567E-26
1732	1	rs76311252	174640594	1	rs3767397	202534389	1.567E-26
1733	1	rs76311252	174640594	1	rs3767395	202536140	1.567E-26
1734	1	rs77714297	174641617	1	rs6427950	202472066	1.567E-26
1735	1	rs77714297	174641617	1	rs3753908	202511295	1.567E-26
1736	1	rs77714297	174641617	1	rs2249811	202531741	1.567E-26
1737	1	rs77714297	174641617	1	rs925532	202533880	1.567E-26
1738	1	rs77714297	174641617	1	rs925533	202534108	1.567E-26
1739	1	rs77714297	174641617	1	rs3767397	202534389	1.567E-26
1740	1	rs77714297	174641617	1	rs3767395	202536140	1.567E-26
1741	1	rs75540548	174648680	1	rs6427950	202472066	1.567E-26
1742	1	rs75540548	174648680	1	rs3753908	202511295	1.567E-26
1743	1	rs75540548	174648680	1	rs2249811	202531741	1.567E-26
1744	1	rs75540548	174648680	1	rs925532	202533880	1.567E-26
1745	1	rs75540548	174648680	1	rs925533	202534108	1.567E-26
1746	1	rs75540548	174648680	1	rs3767397	202534389	1.567E-26
1747	1	rs75540548	174648680	1	rs3767395	202536140	1.567E-26
1748	1	rs79728530	174402618	1	rs10159280	202394287	1.637E-26
1749	1	rs2179108	174540727	1	rs930734	202318316	1.651E-26
1750	1	rs78583223	174766014	1	rs10465591	202308344	1.769E-26
1751	1	rs2179108	174540727	1	rs2361453	202336314	1.804E-26
1752	1	rs146397359	174816494	1	rs6427950	202472066	1.841E-26
1753	1	rs146397359	174816494	1	rs3753908	202511295	1.841E-26
1754	1	rs146397359	174816494	1	rs2249811	202531741	1.841E-26
1755	1	rs146397359	174816494	1	rs925532	202533880	1.841E-26
1756	1	rs146397359	174816494	1	rs925533	202534108	1.841E-26
1757	1	rs146397359	174816494	1	rs3767397	202534389	1.841E-26
1758	1	rs146397359	174816494	1	rs3767395	202536140	1.841E-26
1759	1	rs2179108	174540727	1	rs6427950	202472066	1.897E-26
1760	1	rs2179108	174540727	1	rs3753908	202511295	1.897E-26
1761	1	rs2179108	174540727	1	rs2249811	202531741	1.897E-26
1762	1	rs2179108	174540727	1	rs925532	202533880	1.897E-26
1763	1	rs2179108	174540727	1	rs925533	202534108	1.897E-26

1764	1	rs2179108	174540727	1	rs3767397	202534389	1.897E-26
1765	1	rs2179108	174540727	1	rs3767395	202536140	1.897E-26
1766	1	rs74761236	174619114	1	rs1890875	202531366	1.926E-26
1767	1	rs149356780	174619156	1	rs1890875	202531366	1.926E-26
1768	1	rs146122202	174619399	1	rs1890875	202531366	1.926E-26
1769	1	rs78172621	174624110	1	rs1890875	202531366	1.926E-26
1770	1	rs74641552	174635904	1	rs1890875	202531366	1.926E-26
1771	1	rs115697417	174639688	1	rs1890875	202531366	1.926E-26
1772	1	rs76311252	174640594	1	rs1890875	202531366	1.926E-26
1773	1	rs77714297	174641617	1	rs1890875	202531366	1.926E-26
1774	1	rs75540548	174648680	1	rs1890875	202531366	1.926E-26
1775	1	rs74761236	174619114	1	rs10800836	202410429	2.019E-26
1776	1	rs74761236	174619114	1	rs3767425	202413840	2.019E-26
1777	1	rs74761236	174619114	1	rs3767421	202432160	2.019E-26
1778	1	rs149356780	174619156	1	rs10800836	202410429	2.019E-26
1779	1	rs149356780	174619156	1	rs3767425	202413840	2.019E-26
1780	1	rs149356780	174619156	1	rs3767421	202432160	2.019E-26
1781	1	rs146122202	174619399	1	rs10800836	202410429	2.019E-26
1782	1	rs146122202	174619399	1	rs3767425	202413840	2.019E-26
1783	1	rs146122202	174619399	1	rs3767421	202432160	2.019E-26
1784	1	rs78172621	174624110	1	rs10800836	202410429	2.019E-26
1785	1	rs78172621	174624110	1	rs3767425	202413840	2.019E-26
1786	1	rs78172621	174624110	1	rs3767421	202432160	2.019E-26
1787	1	rs74641552	174635904	1	rs10800836	202410429	2.019E-26
1788	1	rs74641552	174635904	1	rs3767425	202413840	2.019E-26
1789	1	rs74641552	174635904	1	rs3767421	202432160	2.019E-26
1790	1	rs115697417	174639688	1	rs10800836	202410429	2.019E-26
1791	1	rs115697417	174639688	1	rs3767425	202413840	2.019E-26
1792	1	rs115697417	174639688	1	rs3767421	202432160	2.019E-26
1793	1	rs76311252	174640594	1	rs10800836	202410429	2.019E-26
1794	1	rs76311252	174640594	1	rs3767425	202413840	2.019E-26
1795	1	rs76311252	174640594	1	rs3767421	202432160	2.019E-26
1796	1	rs77714297	174641617	1	rs10800836	202410429	2.019E-26
1797	1	rs77714297	174641617	1	rs3767425	202413840	2.019E-26
1798	1	rs77714297	174641617	1	rs3767421	202432160	2.019E-26
1799	1	rs75540548	174648680	1	rs10800836	202410429	2.019E-26
1800	1	rs75540548	174648680	1	rs3767425	202413840	2.019E-26
1801	1	rs75540548	174648680	1	rs3767421	202432160	2.019E-26
1802	1	rs74225911	174519824	1	rs10465591	202308344	2.102E-26
1803	1	rs6703536	174613295	1	rs930734	202318316	2.151E-26
1804	1	rs74761236	174619114	1	rs10920392	202321123	2.157E-26
1805	1	rs74761236	174619114	1	rs6658708	202322113	2.157E-26
1806	1	rs149356780	174619156	1	rs10920392	202321123	2.157E-26
1807	1	rs149356780	174619156	1	rs6658708	202322113	2.157E-26
1808	1	rs146122202	174619399	1	rs10920392	202321123	2.157E-26
1809	1	rs146122202	174619399	1	rs6658708	202322113	2.157E-26
1810	1	rs78172621	174624110	1	rs10920392	202321123	2.157E-26
1811	1	rs78172621	174624110	1	rs6658708	202322113	2.157E-26
1812	1	rs74641552	174635904	1	rs10920392	202321123	2.157E-26
1813	1	rs74641552	174635904	1	rs6658708	202322113	2.157E-26
1814	1	rs115697417	174639688	1	rs10920392	202321123	2.157E-26
1815	1	rs115697417	174639688	1	rs6658708	202322113	2.157E-26

1816	1	rs76311252	174640594	1	rs10920392	202321123	2.157E-26
1817	1	rs76311252	174640594	1	rs6658708	202322113	2.157E-26
1818	1	rs77714297	174641617	1	rs10920392	202321123	2.157E-26
1819	1	rs77714297	174641617	1	rs6658708	202322113	2.157E-26
1820	1	rs75540548	174648680	1	rs10920392	202321123	2.157E-26
1821	1	rs75540548	174648680	1	rs6658708	202322113	2.157E-26
1822	1	rs2179108	174540727	1	rs1890875	202531366	2.3E-26
1823	1	rs6703536	174613295	1	rs2361453	202336314	2.371E-26
1824	1	rs2179108	174540727	1	rs10800836	202410429	2.399E-26
1825	1	rs2179108	174540727	1	rs3767425	202413840	2.399E-26
1826	1	rs2179108	174540727	1	rs3767421	202432160	2.399E-26
1827	1	rs6703536	174613295	1	rs6427950	202472066	2.566E-26
1828	1	rs6703536	174613295	1	rs3753908	202511295	2.566E-26
1829	1	rs6703536	174613295	1	rs2249811	202531741	2.566E-26
1830	1	rs6703536	174613295	1	rs925532	202533880	2.566E-26
1831	1	rs6703536	174613295	1	rs925533	202534108	2.566E-26
1832	1	rs6703536	174613295	1	rs3767397	202534389	2.566E-26
1833	1	rs6703536	174613295	1	rs3767395	202536140	2.566E-26
1834	1	rs2179108	174540727	1	rs10920392	202321123	2.584E-26
1835	1	rs2179108	174540727	1	rs6658708	202322113	2.584E-26
1836	1	rs74761236	174619114	1	rs10159280	202394287	2.649E-26
1837	1	rs149356780	174619156	1	rs10159280	202394287	2.649E-26
1838	1	rs146122202	174619399	1	rs10159280	202394287	2.649E-26
1839	1	rs78172621	174624110	1	rs10159280	202394287	2.649E-26
1840	1	rs74641552	174635904	1	rs10159280	202394287	2.649E-26
1841	1	rs115697417	174639688	1	rs10159280	202394287	2.649E-26
1842	1	rs76311252	174640594	1	rs10159280	202394287	2.649E-26
1843	1	rs77714297	174641617	1	rs10159280	202394287	2.649E-26
1844	1	rs75540548	174648680	1	rs10159280	202394287	2.649E-26
1845	1	rs2179108	174540727	1	rs10159280	202394287	3.132E-26
1846	1	rs6703536	174613295	1	rs1890875	202531366	3.151E-26
1847	1	rs6703536	174613295	1	rs10800836	202410429	3.298E-26
1848	1	rs6703536	174613295	1	rs3767425	202413840	3.298E-26
1849	1	rs6703536	174613295	1	rs3767421	202432160	3.298E-26
1850	1	rs79387712	174605087	1	rs10465591	202308344	3.336E-26
1851	1	rs6703536	174613295	1	rs10920392	202321123	3.521E-26
1852	1	rs6703536	174613295	1	rs6658708	202322113	3.521E-26
1853	1	rs187157646	174643586	1	rs930734	202318316	3.724E-26
1854	1	rs12404552	202566780	3	rs6791363	85073170	3.947E-26
1855	1	rs79728530	174402618	1	rs10465591	202308344	3.989E-26
1856	1	rs187157646	174643586	1	rs2361453	202336314	4.099E-26
1857	1	rs6703536	174613295	1	rs10159280	202394287	4.32E-26
1858	1	rs187157646	174643586	1	rs2292489	202536848	4.718E-26
1859	1	rs4515855	174588920	1	rs2292489	202536848	5.783E-26
1860	1	rs187157646	174643586	1	rs10920392	202321123	6.144E-26
1861	1	rs187157646	174643586	1	rs6658708	202322113	6.144E-26
1862	1	rs7543502	174653701	1	rs705742	202461982	6.454E-26
1863	1	rs74761236	174619114	1	rs10465591	202308344	6.466E-26
1864	1	rs149356780	174619156	1	rs10465591	202308344	6.466E-26
1865	1	rs146122202	174619399	1	rs10465591	202308344	6.466E-26
1866	1	rs78172621	174624110	1	rs10465591	202308344	6.466E-26
1867	1	rs74641552	174635904	1	rs10465591	202308344	6.466E-26

1868	1	rs115697417	174639688	1	rs10465591	202308344	6.466E-26
1869	1	rs76311252	174640594	1	rs10465591	202308344	6.466E-26
1870	1	rs77714297	174641617	1	rs10465591	202308344	6.466E-26
1871	1	rs75540548	174648680	1	rs10465591	202308344	6.466E-26
1872	1	rs2179108	174540727	1	rs10465591	202308344	6.993E-26
1873	1	rs187157646	174643586	1	rs10159280	202394287	7.565E-26
1874	1	rs146397359	174816494	1	rs1890875	202531366	7.923E-26
1875	1	rs187157646	174643586	1	rs10800836	202410429	9.211E-26
1876	1	rs187157646	174643586	1	rs3767425	202413840	9.211E-26
1877	1	rs187157646	174643586	1	rs3767421	202432160	9.211E-26
1878	1	rs187157646	174643586	1	rs6427950	202472066	9.211E-26
1879	1	rs187157646	174643586	1	rs3753908	202511295	9.211E-26
1880	1	rs187157646	174643586	1	rs2249811	202531741	9.211E-26
1881	1	rs187157646	174643586	1	rs925532	202533880	9.211E-26
1882	1	rs187157646	174643586	1	rs925533	202534108	9.211E-26
1883	1	rs187157646	174643586	1	rs3767397	202534389	9.211E-26
1884	1	rs187157646	174643586	1	rs3767395	202536140	9.211E-26
1885	1	rs79893841	174622255	1	rs2292489	202536848	9.318E-26
1886	1	rs4515855	174588920	1	rs930734	202318316	9.326E-26
1887	1	rs4515855	174588920	1	rs2361453	202336314	1.013E-25
1888	1	rs6703536	174613295	1	rs10465591	202308344	1.046E-25
1889	1	rs75461537	174869593	1	rs705742	202461982	1.084E-25
1890	1	rs4515855	174588920	1	rs6427950	202472066	1.117E-25
1891	1	rs4515855	174588920	1	rs3753908	202511295	1.117E-25
1892	1	rs4515855	174588920	1	rs2249811	202531741	1.117E-25
1893	1	rs4515855	174588920	1	rs925532	202533880	1.117E-25
1894	1	rs4515855	174588920	1	rs925533	202534108	1.117E-25
1895	1	rs4515855	174588920	1	rs3767397	202534389	1.117E-25
1896	1	rs4515855	174588920	1	rs3767395	202536140	1.117E-25
1897	1	rs187157646	174643586	1	rs1890875	202531366	1.134E-25
1898	1	rs146397359	174816494	1	rs10800836	202410429	1.276E-25
1899	1	rs146397359	174816494	1	rs3767425	202413840	1.276E-25
1900	1	rs146397359	174816494	1	rs3767421	202432160	1.276E-25
1901	1	rs2362941	202550547	5	rs265973	174860699	1.303E-25
1902	1	rs4515855	174588920	1	rs1890875	202531366	1.374E-25
1903	1	rs12404552	202566780	3	rs9876378	85071278	1.422E-25
1904	1	rs4515855	174588920	1	rs10800836	202410429	1.433E-25
1905	1	rs4515855	174588920	1	rs3767425	202413840	1.433E-25
1906	1	rs4515855	174588920	1	rs3767421	202432160	1.433E-25
1907	1	rs79893841	174622255	1	rs930734	202318316	1.495E-25
1908	1	rs4515855	174588920	1	rs10920392	202321123	1.513E-25
1909	1	rs4515855	174588920	1	rs6658708	202322113	1.513E-25
1910	1	rs79893841	174622255	1	rs2361453	202336314	1.643E-25
1911	1	rs79893841	174622255	1	rs6427950	202472066	1.779E-25
1912	1	rs79893841	174622255	1	rs3753908	202511295	1.779E-25
1913	1	rs79893841	174622255	1	rs2249811	202531741	1.779E-25
1914	1	rs79893841	174622255	1	rs925532	202533880	1.779E-25
1915	1	rs79893841	174622255	1	rs925533	202534108	1.779E-25
1916	1	rs79893841	174622255	1	rs3767397	202534389	1.779E-25
1917	1	rs79893841	174622255	1	rs3767395	202536140	1.779E-25
1918	1	rs4515855	174588920	1	rs10159280	202394287	1.86E-25
1919	1	rs187157646	174643586	1	rs10465591	202308344	1.888E-25

1920	1	rs4950778	202431464	3	rs115821677	86219871	2.171E-25
1921	1	rs4950779	202542202	3	rs115821677	86219871	2.171E-25
1922	1	rs79893841	174622255	1	rs1890875	202531366	2.18E-25
1923	1	rs79893841	174622255	1	rs10800836	202410429	2.281E-25
1924	1	rs79893841	174622255	1	rs3767425	202413840	2.281E-25
1925	1	rs79893841	174622255	1	rs3767421	202432160	2.281E-25
1926	1	rs79893841	174622255	1	rs10920392	202321123	2.432E-25
1927	1	rs79893841	174622255	1	rs6658708	202322113	2.432E-25
1928	1	rs146397359	174816494	1	rs2292489	202536848	2.907E-25
1929	1	rs79893841	174622255	1	rs10159280	202394287	2.977E-25
1930	1	rs967677	202548822	4	rs6853599	3445610	3.03E-25
1931	1	rs6700434	174868328	1	rs705742	202461982	3.358E-25
1932	1	rs4515855	174588920	1	rs10465591	202308344	4.527E-25
1933	1	rs77551912	174873951	1	rs705742	202461982	8.204E-25
1934	1	rs2362941	202550547	4	rs6853599	3445610	2.257E-24
1935	1	rs967677	202548822	3	rs12632810	10824072	2.487E-24
1936	1	rs967677	202548822	3	rs12632152	10832600	2.537E-24
1937	1	rs967677	202548822	3	rs2017887	10825694	2.57E-24
1938	1	rs747640	202551071	4	rs45579537	3446284	4.112E-24
1939	1	rs146397359	174816494	1	rs10920392	202321123	5.837E-24
1940	1	rs146397359	174816494	1	rs6658708	202322113	5.837E-24
1941	1	rs182548672	202440828	3	rs73141547	85657125	8.361E-24
1942	1	rs77155802	202450864	3	rs73141547	85657125	8.361E-24
1943	1	rs146397359	174816494	1	rs10465591	202308344	9.672E-24
1944	1	rs146397359	174816494	1	rs2361453	202336314	1.049E-23
1945	1	rs76616345	174766778	1	rs705742	202461982	1.102E-23
1946	1	rs2741849	202373394	4	rs283420	100288975	1.361E-23
1947	1	rs747640	202551071	4	rs17805201	3446513	1.507E-23
1948	1	rs146397359	174816494	1	rs10159280	202394287	1.983E-23
1949	1	rs146397359	174816494	1	rs930734	202318316	2.308E-23
1950	1	rs705742	202461982	3	rs9288991	113855761	4.533E-23
1951	1	rs705742	202461982	3	rs57309847	113856802	4.533E-23
1952	1	rs705742	202461982	3	rs9859762	113857118	4.533E-23
1953	1	rs12404552	202566780	3	rs1691471	85011013	6.085E-23
1954	1	rs705742	202461982	3	rs4592996	113850489	6.213E-23
1955	1	rs705742	202461982	3	rs2654754	113855796	6.213E-23
1956	1	rs705742	202461982	3	rs9837524	113856611	6.274E-23
1957	1	rs2741849	202373394	4	rs283416	100271356	1.161E-22
1958	1	rs2741849	202373394	4	rs166892	100282765	1.161E-22
1959	1	rs2741849	202373394	4	rs1229852	100283665	1.161E-22
1960	1	rs2741849	202373394	4	rs283422	100287182	1.161E-22
1961	1	rs2741849	202373394	4	rs283421	100287996	1.161E-22
1962	1	rs2741849	202373394	4	rs283418	100292187	1.161E-22
1963	1	rs705742	202461982	3	rs12490922	113856881	1.165E-22
1964	1	rs705742	202461982	3	rs9824856	113852731	1.788E-22
1965	1	rs4950859	202552839	3	rs12632810	10824072	1.873E-22
1966	1	rs4950859	202552839	3	rs12632152	10832600	1.886E-22
1967	1	rs4950859	202552839	3	rs2017887	10825694	1.935E-22
1968	1	rs747640	202551071	3	rs12632810	10824072	2.5E-22
1969	1	rs747640	202551071	3	rs12632152	10832600	2.516E-22
1970	1	rs747640	202551071	3	rs2017887	10825694	2.582E-22
1971	1	rs6426901	165158705	1	rs930734	202318316	9.938E-22

1972	1	rs6426902	165158935	1	rs930734	202318316	9.938E-22
1973	1	rs6426901	165158705	1	rs2361453	202336314	1.155E-21
1974	1	rs6426902	165158935	1	rs2361453	202336314	1.155E-21
1975	1	rs2741849	202373394	4	rs283411	100265957	1.201E-21
1976	1	rs2741849	202373394	4	rs72681904	100245080	1.288E-21
1977	1	rs6426901	165158705	1	rs2292489	202536848	2.021E-21
1978	1	rs6426902	165158935	1	rs2292489	202536848	2.021E-21
1979	1	rs3767392	202547101	3	rs12632152	10832600	2.478E-21
1980	1	rs3767392	202547101	3	rs12632810	10824072	2.483E-21
1981	1	rs3767392	202547101	3	rs2017887	10825694	2.507E-21
1982	1	rs10753665	165154104	1	rs2361453	202336314	3.584E-21
1983	1	rs10753665	165154104	1	rs930734	202318316	5.297E-21
1984	1	rs2741849	202373394	4	rs283404	100280719	1.329E-20
1985	1	rs79728530	174402618	1	rs705742	202461982	1.964E-20
1986	1	rs3767409	202479192	4	rs113221089	3445179	3.305E-20
1987	1	rs60885521	174591547	1	rs705742	202461982	3.843E-20
1988	1	rs10753665	165154104	1	rs2292489	202536848	7.109E-20
1989	1	rs79893841	174622255	1	rs705742	202461982	1.263E-19
1990	1	rs74649124	174760589	1	rs705742	202461982	1.335E-19
1991	1	rs79258314	174760696	1	rs705742	202461982	1.335E-19
1992	1	rs78199276	174706357	1	rs705742	202461982	2.287E-18
1993	1	rs6695956	174711947	1	rs705742	202461982	2.287E-18
1994	1	rs77727672	174718575	1	rs705742	202461982	2.287E-18
1995	1	rs79388999	174724332	1	rs705742	202461982	2.287E-18
1996	1	rs61329817	174724485	1	rs705742	202461982	2.287E-18
1997	1	rs6703536	174613295	1	rs705742	202461982	3.162E-18
1998	1	rs12404552	202566780	3	rs6803322	84986088	3.414E-18
1999	1	rs74761236	174619114	1	rs705742	202461982	4.867E-18
2000	1	rs149356780	174619156	1	rs705742	202461982	4.867E-18
2001	1	rs146122202	174619399	1	rs705742	202461982	4.867E-18
2002	1	rs78172621	174624110	1	rs705742	202461982	4.867E-18
2003	1	rs74641552	174635904	1	rs705742	202461982	4.867E-18
2004	1	rs115697417	174639688	1	rs705742	202461982	4.867E-18
2005	1	rs76311252	174640594	1	rs705742	202461982	4.867E-18
2006	1	rs77714297	174641617	1	rs705742	202461982	4.867E-18
2007	1	rs75540548	174648680	1	rs705742	202461982	4.867E-18
2008	1	rs74225911	174519824	1	rs705742	202461982	1.308E-17
2009	1	rs16847018	174419672	1	rs705742	202461982	1.868E-17
2010	1	rs16847025	174425858	1	rs705742	202461982	1.868E-17
2011	1	rs75368101	174447029	1	rs705742	202461982	1.868E-17
2012	1	rs74367921	174457505	1	rs705742	202461982	1.868E-17
2013	1	rs141587031	174458302	1	rs705742	202461982	1.868E-17
2014	1	rs75222047	174460751	1	rs705742	202461982	1.868E-17
2015	1	rs77684114	174461386	1	rs705742	202461982	1.868E-17
2016	1	rs79170014	174462952	1	rs705742	202461982	1.868E-17
2017	1	rs2901819	174467697	1	rs705742	202461982	1.868E-17
2018	1	rs58063264	174468442	1	rs705742	202461982	1.868E-17
2019	1	rs41397546	174476714	1	rs705742	202461982	1.868E-17
2020	1	rs16847121	174482433	1	rs705742	202461982	1.868E-17
2021	1	rs76886469	174488738	1	rs705742	202461982	1.868E-17
2022	1	rs57132183	174492721	1	rs705742	202461982	1.868E-17
2023	1	rs74651209	174501445	1	rs705742	202461982	1.868E-17

2024	1	rs59262554	174504233	1	rs705742	202461982	1.868E-17
2025	1	rs7547843	174507529	1	rs705742	202461982	1.868E-17
2026	1	rs142531097	174512268	1	rs705742	202461982	1.868E-17
2027	1	rs146786952	174514535	1	rs705742	202461982	1.868E-17
2028	1	rs41266052	174517443	1	rs705742	202461982	1.868E-17
2029	1	rs57898822	174531784	1	rs705742	202461982	1.868E-17
2030	1	rs75120198	174535022	1	rs705742	202461982	1.868E-17
2031	1	rs75134210	174536483	1	rs705742	202461982	1.868E-17
2032	1	rs2179109	174540259	1	rs705742	202461982	1.868E-17
2033	1	rs16847206	174541307	1	rs705742	202461982	1.868E-17
2034	1	rs148694367	174544318	1	rs705742	202461982	1.868E-17
2035	1	rs143179310	174545451	1	rs705742	202461982	1.868E-17
2036	1	rs79763699	174729671	1	rs705742	202461982	1.877E-17
2037	1	rs139161617	174732199	1	rs705742	202461982	1.877E-17
2038	1	rs6673649	174733562	1	rs705742	202461982	1.877E-17
2039	1	rs75912125	174740527	1	rs705742	202461982	1.877E-17
2040	1	rs77586561	174315488	1	rs17492329	202461103	3.264E-17
2041	1	rs6692753	174737457	1	rs705742	202461982	6.343E-17
2042	1	rs12404552	202566780	3	rs6771804	84983918	6.754E-17
2043	1	rs114289262	174587137	1	rs705742	202461982	7.496E-17
2044	1	rs56864802	174568929	1	rs705742	202461982	8.596E-17
2045	1	rs60240702	174569231	1	rs705742	202461982	8.596E-17
2046	1	rs77531515	174572000	1	rs705742	202461982	8.596E-17
2047	1	rs77025642	174572929	1	rs705742	202461982	8.596E-17
2048	1	rs79989938	174575187	1	rs705742	202461982	8.596E-17
2049	1	rs80324733	174776328	1	rs705742	202461982	9.138E-17
2050	1	rs12404552	202566780	3	rs28693686	84974785	9.475E-17
2051	1	rs3753562	174923567	1	rs705742	202461982	1.192E-16
2052	1	rs138190123	174555301	1	rs705742	202461982	1.637E-16
2053	1	rs78583223	174766014	1	rs17492329	202461103	3.485E-16
2054	1	rs58762254	174780210	1	rs705742	202461982	4.83E-16
2055	1	rs61028529	174787899	1	rs705742	202461982	4.83E-16
2056	1	rs79203634	174793526	1	rs705742	202461982	4.83E-16
2057	1	rs74524401	174795281	1	rs705742	202461982	4.83E-16
2058	1	rs59190079	174594608	1	rs705742	202461982	5.032E-16
2059	1	rs187157646	174643586	1	rs705742	202461982	7.667E-16
2060	1	rs76912418	174273097	1	rs17492329	202461103	8.277E-16
2061	1	rs78583223	174766014	1	rs705742	202461982	1.027E-15
2062	1	rs57425860	174335876	1	rs17492329	202461103	1.095E-15
2063	1	rs80134637	174749551	1	rs705742	202461982	1.467E-15
2064	1	rs76358862	174297060	1	rs17492329	202461103	1.678E-15
2065	1	rs59436654	174746513	1	rs705742	202461982	1.723E-15
2066	1	rs74225904	174260843	1	rs17492329	202461103	1.844E-15
2067	1	rs77915633	174268968	1	rs17492329	202461103	1.844E-15
2068	1	rs75109372	174275765	1	rs17492329	202461103	1.844E-15
2069	1	rs58657641	174290586	1	rs17492329	202461103	1.844E-15
2070	1	rs79842710	174296950	1	rs17492329	202461103	1.844E-15
2071	1	rs59949376	174298821	1	rs17492329	202461103	1.844E-15
2072	1	rs79273006	174306403	1	rs17492329	202461103	1.844E-15
2073	1	rs7549724	174311135	1	rs17492329	202461103	1.844E-15
2074	1	rs74982851	174322037	1	rs17492329	202461103	1.844E-15
2075	1	rs74838601	174330367	1	rs17492329	202461103	1.844E-15

2076	1	rs78735014	174337971	1	rs17492329	202461103	1.844E-15
2077	1	rs58525721	174344965	1	rs17492329	202461103	1.844E-15
2078	1	rs80232010	174346619	1	rs17492329	202461103	1.844E-15
2079	1	rs75694709	174352895	1	rs17492329	202461103	1.844E-15
2080	1	rs75455128	174354080	1	rs17492329	202461103	1.844E-15
2081	1	rs78840512	174367013	1	rs17492329	202461103	1.844E-15
2082	1	rs78176042	174367254	1	rs17492329	202461103	1.844E-15
2083	1	rs79039367	174376971	1	rs17492329	202461103	1.844E-15
2084	1	rs57053120	174830490	1	rs705742	202461982	2.528E-15
2085	1	rs76828045	174847442	1	rs705742	202461982	2.934E-15
2086	1	rs147728966	174848270	1	rs705742	202461982	2.934E-15
2087	1	rs76991270	174267626	1	rs17492329	202461103	3.009E-15
2088	1	rs80143117	174256505	1	rs17492329	202461103	3.275E-15
2089	1	rs59061054	174244009	1	rs17492329	202461103	3.452E-15
2090	1	rs80196237	174049290	1	rs17492329	202461103	6.268E-15
2091	1	rs141729655	174432399	1	rs705742	202461982	1.126E-14
2092	1	rs79077452	174435344	1	rs705742	202461982	1.126E-14
2093	1	rs79728530	174402618	1	rs17492329	202461103	1.185E-14
2094	1	rs60723087	174655617	1	rs17492329	202461103	1.186E-14
2095	1	rs75062924	174664341	1	rs17492329	202461103	1.186E-14
2096	1	rs74739388	174666752	1	rs17492329	202461103	1.186E-14
2097	1	rs74225917	174686241	1	rs17492329	202461103	1.186E-14
2098	1	rs59354288	174687003	1	rs17492329	202461103	1.186E-14
2099	1	rs60665754	174687287	1	rs17492329	202461103	1.186E-14
2100	1	rs76014701	174690404	1	rs17492329	202461103	1.186E-14
2101	1	rs74365829	174690558	1	rs17492329	202461103	1.186E-14
2102	1	rs7516351	174062741	1	rs17492329	202461103	1.342E-14
2103	1	rs59259904	174071417	1	rs17492329	202461103	1.342E-14
2104	1	rs58911640	174078545	1	rs17492329	202461103	1.342E-14
2105	1	rs80065868	174084151	1	rs17492329	202461103	1.342E-14
2106	1	rs74634566	174084903	1	rs17492329	202461103	1.342E-14
2107	1	rs4515855	174588920	1	rs705742	202461982	1.702E-14
2108	1	rs7543502	174653701	1	rs17492329	202461103	2.163E-14
2109	1	rs4284300	174463049	1	rs12404552	202566780	2.601E-14
2110	1	rs79387712	174605087	1	rs705742	202461982	2.603E-14
2111	1	rs79763699	174729671	1	rs17492329	202461103	2.65E-14
2112	1	rs139161617	174732199	1	rs17492329	202461103	2.65E-14
2113	1	rs6673649	174733562	1	rs17492329	202461103	2.65E-14
2114	1	rs75912125	174740527	1	rs17492329	202461103	2.65E-14
2115	1	rs78199276	174706357	1	rs17492329	202461103	2.733E-14
2116	1	rs6695956	174711947	1	rs17492329	202461103	2.733E-14
2117	1	rs77727672	174718575	1	rs17492329	202461103	2.733E-14
2118	1	rs79388999	174724332	1	rs17492329	202461103	2.733E-14
2119	1	rs61329817	174724485	1	rs17492329	202461103	2.733E-14
